# Identification of miRNAs and their targets by high-throughput sequencing and degradome analysis in cytoplasmic male-sterile line NJCMS1A and its maintainer NJCMS1B of soybean

**DOI:** 10.1186/s12864-015-2352-0

**Published:** 2016-01-05

**Authors:** Xianlong Ding, Jiajia Li, Hao Zhang, Tingting He, Shaohuai Han, Yanwei Li, Shouping Yang, Junyi Gai

**Affiliations:** Soybean Research Institute, National Center for Soybean Improvement, MOA Key Laboratory of Biology and Genetic Improvement of Soybean (General), State Key Laboratory of Crop Genetics and Germplasm Enhancement, Nanjing Agricultural University, Nanjing, Jiangsu 210095 China

**Keywords:** Soybean (*Glycine max* (L.) Merr.), Cytoplasmic male sterility, MicroRNA, High-throughput sequencing, Degradome analysis

## Abstract

**Background:**

Cytoplasmic male sterility (CMS) provides crucial breeding materials that facilitate hybrid seed production in various crops, and thus plays an important role in the study of hybrid vigor (heterosis), in plants. However, the CMS regulatory network in soybean remains unclear. MicroRNAs (miRNAs) play crucial roles in flower and pollen development by targeting genes that regulate their expression in plants. To identify the miRNAs and their targets that exist in the soybean CMS line NJCMS1A and its maintainer NJCMS1B, high-throughput sequencing and degradome analysis were conducted in this study.

**Results:**

Two small RNA libraries were constructed from the flower buds of the soybean CMS line NJCMS1A and its maintainer NJCMS1B. A total of 105 new miRNAs present on the other arm of known pre-miRNAs, 23 new miRNA members, 158 novel miRNAs and 160 high-confidence soybean miRNAs were identified using high-throughput sequencing. Among the identified miRNAs, 101 differentially expressed miRNAs with greater than two-fold changes between NJCMS1A and NJCMS1B were discovered. The different expression levels of selected miRNAs were confirmed by stem-loop quantitative real-time PCR. A degradome analysis showed that 856 targets were predicted to be targeted by 296 miRNAs, including a squamosa promoter-binding protein-like transcription factor family protein, a pentatricopeptide repeat-containing protein, and an auxin response factor, which were previously shown to be involved in floral organ or anther development in plants. Additionally, some targets, including a MADS-box transcription factor, NADP-dependent isocitrate dehydrogenase and NADH-ubiquinone oxidoreductase 24 kDa subunit, were identified, and they may have some relationship with the programmed cell death, reactive oxygen species accumulation and energy deficiencies, which might lead to soybean male sterility.

**Conclusions:**

The present study is the first to use deep sequencing technology to identify miRNAs and their targets in the flower buds of the soybean CMS line NJCMS1A and its maintainer NJCMS1B. The results revealed that the miRNAs might participate in flower and pollen development, which could facilitate our understanding of the molecular mechanisms behind CMS in soybean.

**Electronic supplementary material:**

The online version of this article (doi:10.1186/s12864-015-2352-0) contains supplementary material, which is available to authorized users.

## Background

In higher plants, cytoplasmic male sterility (CMS) is a natural phenomenon resulting from maternal inheritance, mainly by mitochondrial genes and nuclear gene interactions, which lead to pollen abortion but normal pistil development [1–3]. At present, ~200 species of plants are known to exhibit CMS [4], which has been widely used in crops, especially in rice heterosis utilization and crop breeding. Many studies have reported that CMS-related genes are located in the mitochondrial genome, and that its F_1_ hybrid fertility could be recovered by a restorer gene or genes originating in the nuclear genome [5]. In 1985, CMS in soybean (*Glycine max* (L.) Merr.) was first reported by Davis [6], and further characterized by Sun et al. [7]. At present, a variety of soybean CMS lines, such as NJCMS1A [8, 9] and NJCMS2A [10], have been developed. With the construction of three lines, CMS, maintainer and restorer, of soybean, many soybean hybrids have been successfully cultivated.

MicroRNAs (miRNAs) are a class of small, endogenous ~21-nt non-coding RNAs. In plants, they are processed from single-stranded RNA precursors that can form stem-loop regions [11, 12]. These miRNAs play a critical role in nearly all biological processes: development, differentiation, and biotic and abiotic stress responses in higher plants, via the degradation or translational inhibition of target mRNAs at transcriptional and post-transcriptional levels [13]. miRNAs have been discovered using three basic approaches: direct cloning, forward genetics and bioinformatics predictions with experimental validation [11]. In recent years, degradome sequencing has been used to identify global targets of miRNAs in *Arabidopsis thaliana* and soybean [14, 15].

To date, hundreds of miRNAs have been isolated by direct cloning or deep sequencing in soybean [16–22], but the number of miRNAs known in soybean is still very small and considerably lower than in *Arabidopsis thaliana* or rice. Additionally, in the past several years, the miRNAs associated with CMS, genetic male sterility or male sterility in plants have been studied, and the results showed that the miRNAs helped regulate male sterility and fertility restoration [23–28]. However, as a widely planted oil crop worldwide, the roles of miRNAs in soybean CMS remain largely unknown. Thus, the identification of miRNAs and the elucidation of their functions in pollen development will help us understand the molecular mechanisms of soybean CMS. Here, we examined and compared the expression profiles of miRNAs in the flower buds of a soybean CMS line NJCMS1A and its maintainer NJCMS1B, using high-throughput sequencing. We also applied degradome sequencing to identify genes targeted by identified miRNAs and analyzed their possible functions. The results may indicate that there were some interactions between miRNAs and their targets, therefore flower bud or pollen development could be influenced by the biogenesis of miRNAs in CMS.

## Results

### Analysis of small RNA library data sets from the flower buds of NJCMS1A and NJCMS1B

**Fig. 1:**
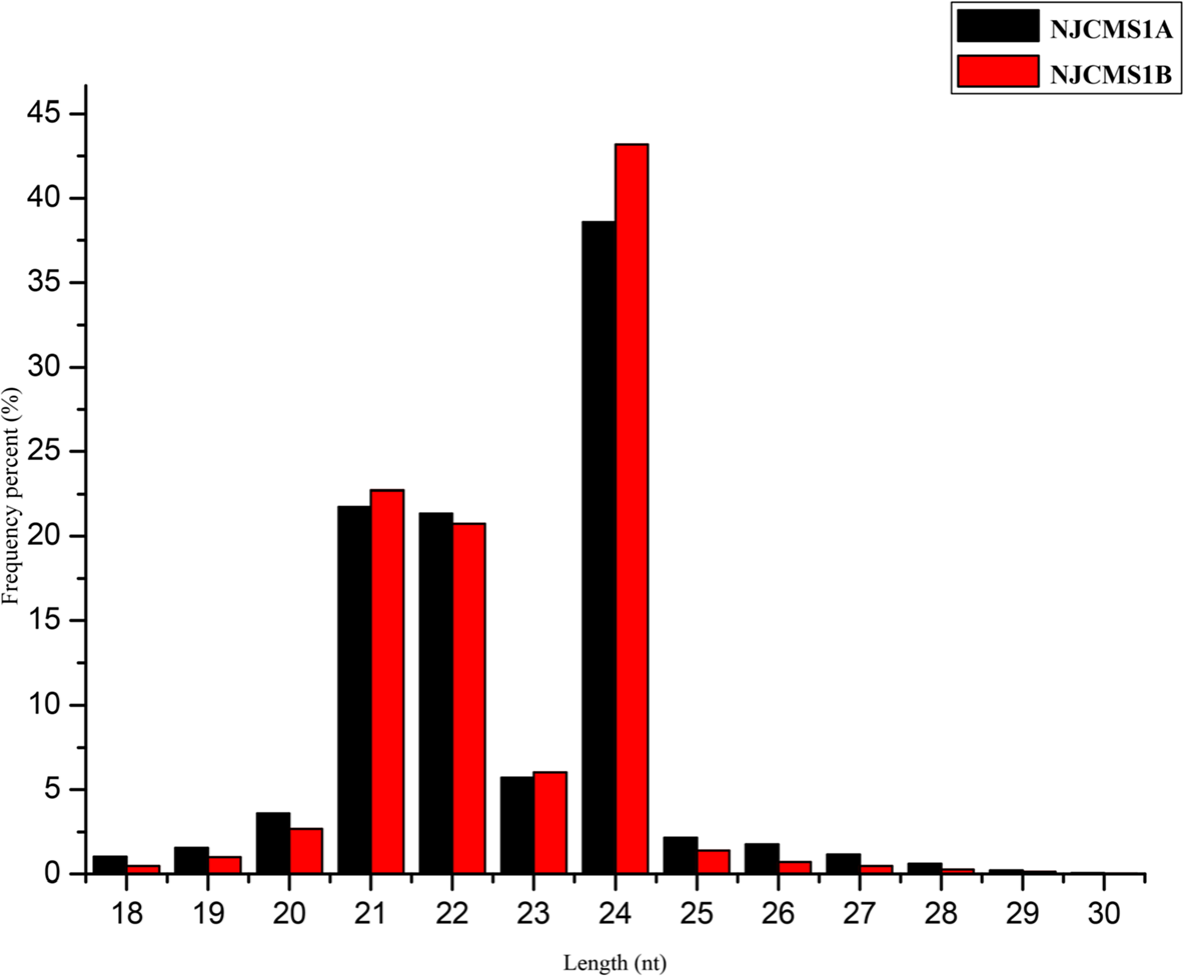
Length distribution of small RNAs found in flower buds from NJCMS1A and NJCMS1B

Two independent small RNA libraries were constructed using RNAs from the flower buds of CMS line NJCMS1A and its maintainer NJCMS1B. A total of 12 104 688 and 12 096 511 raw reads from the two lines, respectively, were generated by high-throughput sequencing (Table 1). After the removal of adaptor contaminants, oversized insertions, low-quality reads, poly A tags and small tags < 18-nt, 11 974 839 and 12 058 427 clean reads, respectively, were obtained with lengths that range from 18–30-nt, and the mappable small RNA sequences were 9 824 678 and 9 758 494, respectively (Table 1). Then, the datasets were used to remove other non-coding RNAs and the degraded fragments of mRNAs (Additional file 1: Table S1). The majority of the small RNAs in the two libraries were 21–24 nt, and the most abundant small RNAs were 24 nt, followed by 21 and 22 nts (Fig. 1), which is within the typical small RNA length distribution for soybean tissues, such as roots, nodules, flowers, developing seeds and cotyledons [19, 22, 29].

**Table 1:** Analysis of small RNA sequences and degradome sequences from NJCMS1A and NJCMS1B

Type			small RNA-Seq		Degradome-Seq	
			NJCMS 1A	NJCMS 1B	NJCMS 1A	NJCMS 1B
Statistical Information	Total tags		12104688 (100.00 %)	12096551 (100.00 %)	12351703 (100.00 %)	12671179 (100.00 %)
	Clean tags		11974839 (98.93 %)	12058427 (99.68 %)	12288476 (99.49 %)	12613736 (99.55 %)
Genome Mapping Statistics	Total Count of Clean Tags	Total tags	11974839 (100.00 %)	12058427 (100.00 %)	12288476 (100.00 %)	12613736 (100.00 %)
		Mapping to genome	9824678 (82.04 %)	9758494 (80.93 %)	10954689 (89.15 %)	11289967 (89.51 %)
	Unique Count of Clean Tags	Total tags	3668753 (100.00 %)	4122100 (100.00 %)	6332697 (100.00 %)	5445882 (100.00 %)
		Mapping to genome	2687857 (73.26 %)	3142047 (76.22 %)	5500905 (86.87 %)	4663560 (85.63 %)

### Identification of known miRNAs

All mappable small RNA sequences were compared with the known soybean miRNAs in the miRBase database (miRBase 21.0, http://www.mirbase.org/). In miRBase 21.0, 573 soybean pre-miRNAs correspond to 639 mature miRNAs. In the present study, 503 pre-miRNAs corresponding to 560 mature miRNAs, were detected that belonged to 222 families (Additional file 1: Table S2). The length of most known miRNAs was 21 nt, followed by 24 and 22 nts (Additional file 2: Figure S1). Among these miRNA families, MIR156 had 24 members, followed by MIR166 with 21 members, but most families had fewer than 10 members (Additional file 1: Table S3, Additional file 2: Figure S2). Interestingly, numerous miRNAs, having additional nucleotides in the 5′ or 3′ termini when compared with the recorded mature miRNAs in miRBase, were detected and could be classified as isomiRNAs. This type of miRNA was previously reported in soybean after high-throughput sequencing [30]. A total of 205 isomiRNAs were discovered in our study and their respective mature miRNAs can be found in Additional file 1: Table S2, with “D” tags.

### Conservation of known miRNAs

Most miRNAs (52.68 %) are conserved among different plant species, and play important and conserved functions in plant development, including flower and pollen development. In contrast, non-conserved miRNAs may play roles in more species-specific characteristics in plant development [31]. In the present study, the known miRNAs belonged to 222 families and 43 of them were conserved in other species (including 12 leguminous-specific miRNA families), while the rest were non-conserved soybean-specific miRNA families (Table 2). Zhang et al. [31] demonstrated that the miRNAs found in more than 10 different plant species were considered highly conserved. Similarly, the miRNA families classified as moderately, lowly and non-conserved miRNAs were found in five to nine, two to four, and only one plant species, respectively [31]. In this study, 27 highly conserved, four moderately conserved and 12 lowly conserved miRNA families were found, in addition, there were 179 non-conserved miRNA families (Additional file 1: Table S4).

**Table 2:** Summary of miRNAs, and their families, found in the flower buds of NJCMS1A and NJCMS1B

Type	Conserved miRNAs		Non-conserved miRNAs		Total
	Found in other plant species	Only found in legume species	With miRNA family information in miRBase 21.0	With no miRNA family information in miRBase 21.0	
No. of known miRNAs	262	33	108	157	560
No. of novel miRNA-3p/miRNA-5p	72	6	15	12	105
No. of new miRNAs members	18	0	2	3	23
No. of novel miRNAs	0	0	0	158	158
No. of known miRNAs families	31	12	36	143	222
No. of novel miRNA-3p/miRNA-5p families	21	4	8	12	45
No. of new miRNAs members families	5	0	1	3	9
No. of novel miRNAs families	0	0	0	111	111

### Novel miRNAs on the other arm of known pre-miRNAs

**Fig. 2:**
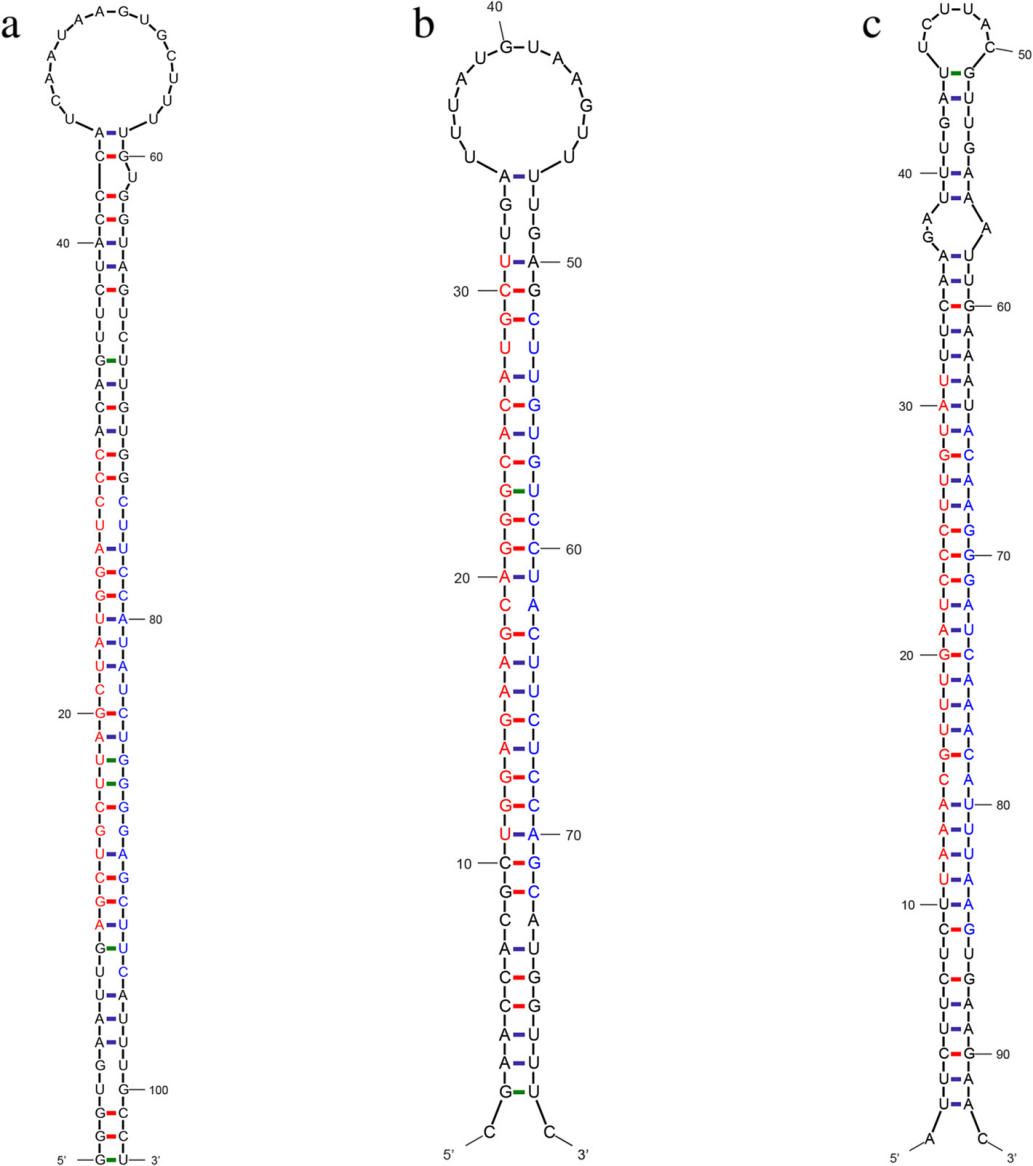
Predicted secondary structures of novel miRNAs from flower buds of NJCMS1A and NJCMS1B*.* **a** gma-miR159d; **b** N-gma-miR164r; and **c** novel_mir_051. Red font represents miRNA-5p, and blue font represents miRNA-3p

Sometimes, the miRNA-3p and miRNA-5p are simultaneously present on the two arms of the pre-miRNA secondary structures, and are considered strong evidence for the existence of these miRNAs in plant. Four criteria described in Methods were used to ensure the accuracy of the results. Through high-throughput sequencing, 105 novel miRNAs on the other arm of known pre-miRNAs were identified (Additional file 1: Table S5), which belonged to 45 families and had not been identified previously (Table 2). Five known miRNAs (gma-miR4378a, gma-miR4407, gma-miR4416c, gma-miR9747 and gma-miR9763) were not obtained in this study, but their miRNAs on the other arm of pre-miRNAs were identified, which were named gma-miR4378a-3p, gma-miR4407-5p, gma-miR4416c-3p, gma-miR9747-5p and gma-miR9763-3p, respectively. The predicted secondary structure of gma-miR159d was selected as an example to be shown in Fig. 2a, and the others are shown in Additional file 2: Figure S3.

### Identification of newly conserved soybean miRNA families and new miRNA members

All unannotated small RNA sequences that matched soybean genome sequences were mapped to known plant miRNAs in miRBase to identify newly conserved miRNA families and new miRNA members. If the small RNAs matched known plant miRNAs with no more than four mismatches, they were classified as candidate conserved miRNAs and new miRNA members [32]. Four characteristics of miRNAs, described in Methods, were used to screen the candidate conserved miRNAs and new miRNA members [20]. Unfortunately, no new conserved miRNA families were found in the present study. However, 23 new miRNA members, belonging to nine known miRNA families (five conserved and four non-conserved miRNA families; Table 2), were discovered in the two small RNA libraries (Table 3). These 23 miRNAs have not been reported as gma-miRNAs in miRBase before but were homologous to known plant miRNAs. The length of most miRNAs was 21 nt, with only one miRNA having a length of 22 nt, which conformed to a common characteristic of plant miRNAs. For N-gma-miR164r, N-gma-miR399i and N-gma-miR399k, both miRNA-3p and miRNA-5p were simultaneously found on the two arms of their pre-miRNAs. Of seven new miRNA members that belonged to MIR164, six shared the same mature sequence, and they had only one base different from gma-miR164r-5p. However, their precursors came from different loci of the soybean genome. A similar result was found for the MIR399 and MIR4380 families (Table 3). The precursor sequences of N-gma-miR164l and N-gma-miR164n, as well as N-gma-miR164m and N-gma-miR164p, were highly similar to each other (Table 3). These types of miRNAs were termed sub-members [25]. The secondary structures of precursors to N-gma-miR164r were selected as the example to be shown in Fig. 2b, and the others are shown in Additional file 2: Figure S4.

**Table 3:** Identification of new miRNA members of known miRNA families

Index	miRNA_name	miRNA_sequence	Length (nt)	MFE (kcal/mol/nt)	MFEI	NJCMS1A_ count	NJCMS1B_ count	3p/5p^a)^
1	N-gma-miR164l	TGGAGAAGGGGAGCACGTGCA	21	−0.53	0.98	12	8	5p
2	N-gma-miR164m	TGGAGAAGGGGAGCACGTGCA	21	−0.52	0.97	12	8	5p
3	N-gma-miR164n	TGGAGAAGGGGAGCACGTGCA	21	−0.54	0.97	12	8	5p
4	N-gma-miR164o	TGGAGAAGGGGAGCACGTGCA	21	−0.53	1.15	12	8	5p
5	N-gma-miR164p	TGGAGAAGGGGAGCACGTGCA	21	−0.48	0.90	12	8	5p
6	N-gma-miR164q	TGGAGAAGGGGAGCACGTGCA	21	−0.49	1.07	12	8	5p
7	N-gma-miR164r-5p	TGGAGAAGCAGGGCACATGCT	21	−0.57	1.20	78	35	5p
8	N-gma-miR164r-3p	CTTGTGTCCTACTTCTCCAGC	21	−0.57	1.20	4	0	5p
9	N-gma-miR156ac	CTGACAGAAGATAGAGAGCAC	21	−0.40	0.86	85	69	5p
10	N-gma-miR395n	CTGAAGTGTTTGGGGGAGCTT	21	−0.41	0.91	5	6	3p
11	N-gma-miR399i-5p	GAGCAATTCTCCTTTGGCAGA	21	−0.48	1.15	2	0	5p
12	N-gma-miR399i-3p	TGCCAAAGGAGAATTGTCCTG	21	−0.48	1.15	6	0	3p
13	N-gma-miR399j	TGCCAAAGGAGATTTGCCCTG	21	−0.44	0.98	0	9	3p
14	N-gma-miR399k-5p	GAGCAAATCTCCATTGGCAGT	21	−0.45	1.01	0	5	5p
15	N-gma-miR399k-3p	TGCCAAAGGAGATTTGCCCTG	21	−0.45	1.01	0	9	3p
16	N-gma-miR399l	TGCCAAAGGAGAGCTGCCCTG	21	−0.43	1.07	97	53	3p
17	N-gma-miR399m	TGCCAAAGGAGAGCTGCCCTG	21	−0.45	1.17	97	53	3p
18	N-gma-miR530f	TGCATTTGCACCTGCGCTTTG	21	−0.47	0.96	8	0	5p
19	N-gma-miR4380c	TGGTTCATACGGATTGTTGAT	21	−0.47	1.12	5	0	3p
20	N-gma-miR4380d	TGGTTCATACGGATTGTTGAT	21	−0.33	1.28	5	0	3p
21	N-gma-miR4348d	TGTCAAACTTGCAAGATGATA	21	−0.45	1.46	0	10	5p
22	N-gma-miR5673b	TGGAATCTCGCGGAAGACATC	21	−0.61	1.27	5	0	3p
23	N-gma-miR5786b	CGTCGCAGGATAGAGGGCACTG	22	−0.39	0.94	0	8	5p

*N* new. a) Arm of this mature miRNA

### Identification of novel miRNAs

To predict novel miRNAs in soybean, we used MIREAP (https://source-forge.net/projects/ mireap/) to explore the secondary structures, Dicer cleavage sites and minimum free energies (MFEs) of the unannotated small RNA sequences that could be mapped to the soybean genome. The unannotated small RNAs that map to the soybean genome, but not to known plant miRNAs, were classified as candidate novel miRNAs. Four criteria described in Methods were used to increase the predictive accuracy. A total of 158 new miRNAs, belonging to 111 novel families (Table 2), were identified in this study. Among them, 20 novel miRNAs on both arms of the pre-miRNAs were identified. The lengths of the mature miRNAs ranged from 20 to 23 nt, and most of them were 21 nt. The precursors of these novel miRNAs were identified by MIREAP and varied from 66 to 354 nt in length, with MFE values ranging from −20.7 to −181.1 kcal/mol (−0.23 to −0.86 kcal/mol/nt). The minimal folding energy indices (MFEI) ranged from 0.85 to 2.14, with 85.53 % of them being greater than 0.97 and the average being 1.32, which is consistent with miRNA. Most of the new mature miRNA sequences (73.58 %) presented a uracil (U) as the first nucleotide. This is in agreement with previous results for soybean [16]. Like new miRNA members, some of the novel miRNAs, such as novel_mir_018a to novel_mir_018f, shared the same mature sequences but had different precursors that came from different loci. The novel-MIR15 had two sub-members (novel_mir_015a and novel_mir_015b). Unlike known miRNAs, the expression levels of the novel miRNAs were very low. The information for all of the novel miRNAs is summarized in Additional file 1: Table S6. The secondary structure for the precursor of novel_mir_51 was used as an example in Fig. 2c, and the others are shown in Additional file 2: Figure S5.

### High-confidence miRNAs in soybean

Until now, no soybean miRNAs had been annotated as high confidence in miRBase 21.0 (http://www.mirbase.org/cgi-bin/mirna_summary.pl?org=gma). To be annotated as a high-confidence miRNA, in addition to the four characteristics of miRNAs described in Methods, a locus must meet the following criteria: first, at least 10 reads map to each of the two mature sequences (miRNA-5p and miRNA-3p), or at least five reads map to each arm and at least 100 reads are mapped in total (miRBase 21.0); and second, at least 50 % of the reads mapping to each arm of the hairpin precursor must have the same 5′ end [33]. Through a combined analysis of known miRNAs with their new 5p or 3p miRNAs, 156 known miRNAs and four novel miRNAs could be annotated as high-confidence miRNAs, representing 23.46 % and 2.53 % of known miRNAs (including new 5p or 3p miRNAs) and novel miRNAs in this study, respectively (Additional file 1: Table S7). The secondary structures for precursors of high-confidence miRNAs are shown in Additional file 2: Figure S6.

### Differential expression profiling of miRNAs and validation by qRT-PCR

Among the identified miRNAs, 101 differentially expressed miRNAs with greater than two-fold relative changes (*P* < 0.05) between NJCMS1A and NJCMS1B were identified by high-throughput sequencing. Compared with NJCMS1B, 35 miRNAs were up-regulated with fold changes > 2 (such as gma-miR1518), and 66 were down-regulated with fold changes < −2 (such as gma-miR1512b) in NJCMS1A. Unlike known miRNAs, many novel miRNAs, including novel_mir_014 and novel_mir_039, were only expressed in NJCMS1A or NJCMS1B, respectively. All information regarding the differential expression profiling of miRNAs can be found in Additional file 1: Table S8.

**Fig. 3:**
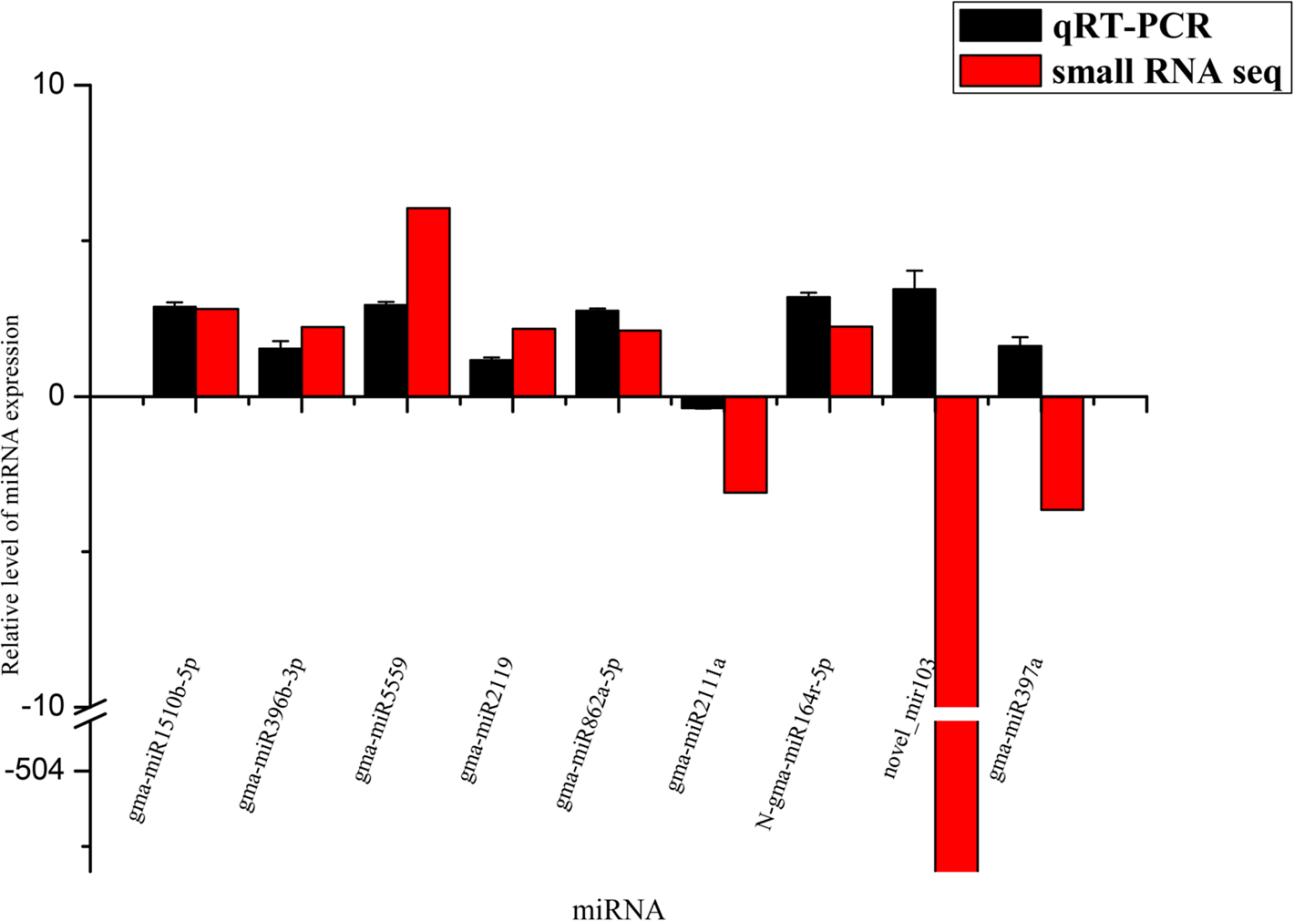
Expression levels of selected miRNA in NJCMS1A and NJCMS1B. The y-axis indicated the miRNA relative expression level generated from qRT-PCR analysis and high-throughput sequencing. The results were obtained from three biological replicates, and the error bars indicated the standard error of the mean of 2^–ΔΔCt^, with NJCMS1B as a control

The stem-loop qRT-PCR was conducted to validate and measure the expression profiles of the nine selected differentially expressed miRNAs. As shown in Fig. 3, seven of them were consistent with the sequencing reads. However, novel_mir103 and gma-miR397a were found to be up-regulated in NJCMS1A, but were enriched in NJCMS1B based on the high-throughput sequencing analysis. The trend was not consistent with that of deep sequencing, possibly because of the low expression levels in the two samples and the difference in sensitivity between qRT-PCR and high-throughput sequencing technology.

### Identification of miRNA target genes in NJCMS1A and NJCMS1B using a degradome analysis

**Fig. 4:**
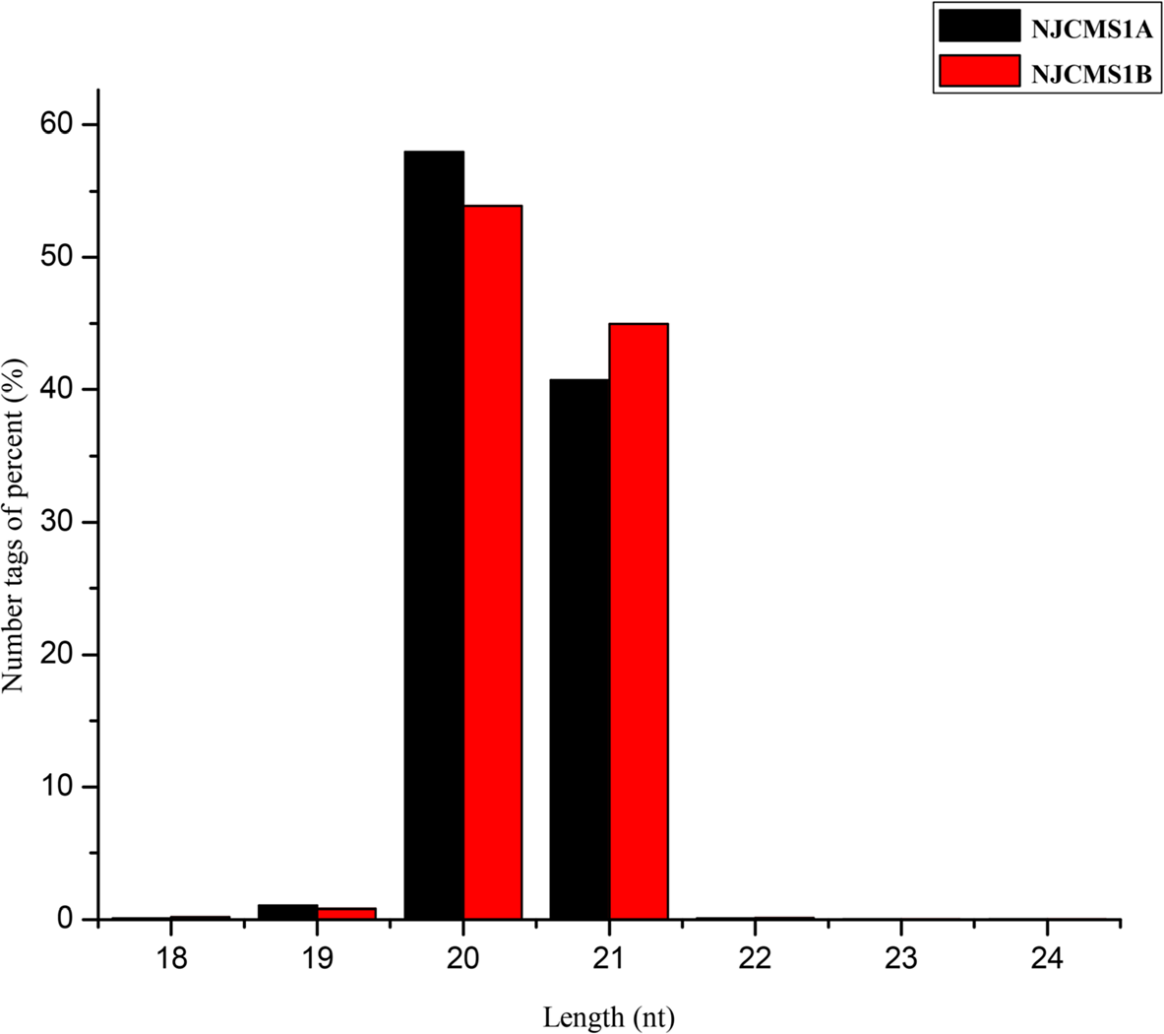
Length distributions of degradome library clean reads in flower buds of NJCMS1A and NJCMS1B

Two independent degradome libraries derived from flower buds of NJCMS1A and NJCMS1B were constructed and sequenced. A total of 12 351 703 and 12 671 179 raw reads were generated from the NJCMS1A and NJCMS1B libraries, respectively (Table 1). After removing the adaptor sequences and/or low-quality reads present in the raw reads, 12 288 476 and 12 613 736 clean reads in the NJCMS1A and NJCMS1B libraries, respectively, were obtained, of which 10 954 689 (89.15 %) and 11 289 967 (89.51 %) were perfectly matched to the soybean genome (Table 1). Moreover, 98.69 % of the clean reads in NJCMS1A and 98.82 % of the clean reads in NJCMS1B were 20 and 21 nts in length, which matched the length distribution peak of the degradome fragments (Fig. 4).

**Fig. 5:**
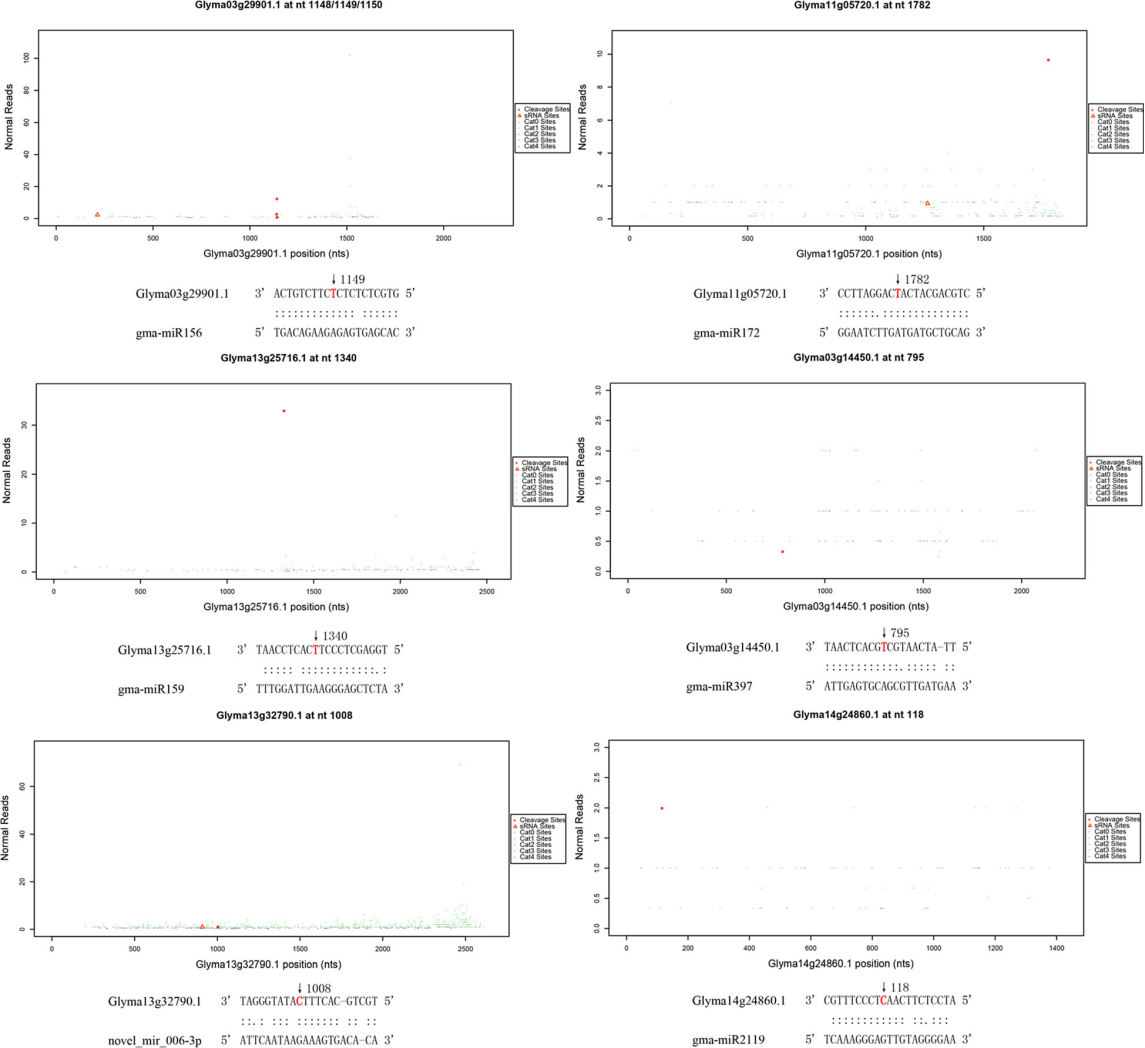
Target plots (*t-plots*) of identified miRNA targets in Fig. 6. The X axis indicated the site position of target cDNA, the Y axis indicated the normal abundance of raw tags. The red circle indicated the precited cleavage site, and the red triangle indicated that the site might combine with miRNA but p≧0.05. The bottom of each t-plot showed the corresponding miRNA:mRNA alignment. The red colored nucleotide on the target transcript indicated the cleavage site and the number next to the arrow in the alignment between the miRNA and the target was the cDNA position coressponds to the detected cleavage site

Based on the distribution of splice sites near raw sequence tags, the splice sites could be divided into five categories: 0 to 4 (Additional file 1: Table S9 and Fig. 5). In total, 856 targets were predicted to be cleaved by 296 miRNAs. Furthermore, 28 novel miRNAs were shown to cleave 81 targets (Additional file 1: Table S10). The targets of all of the miRNAs are listed in Additional file 1: Table S9. These targets included a squamosa promoter-binding protein-like transcription factor family protein, pentatricopeptide repeat-containing (PPR) protein, MADS-box protein, GAMYB protein, auxin response factor, no apical meristem protein, homeobox-leucine zipper protein, nuclear transcription factor Y, transcription factor CCAAT, GRAS family transcription factor, transcription factor APETALA2, TCP transcription factor, F-box family protein, growth-regulating factor, ubiquitin-conjugating enzyme family protein, zinc knuckle (CCHC-type) family protein and basic helix-loop-helix DNA-binding superfamily protein, having essential roles in gene regulation. Some of the targets, such as laccase (LAC) and heat shock 70 kDa protein (Hsp70) etc., may be related to CMS or pollen development. Unfortunately, the target genes of most identified miRNAs could not be detected in the present degradome analysis. In addition, some target genes have unknown functions (Additional file 1: Table S9).

All of the identified miRNA targets were analyzed by gene ontology (GO, http://www.geneontology.org/) to assess the targets’ putative functions and uncover the roles of miRNAs in regulating pollen development. As shown in Additional file 2: Figure S7, the targets were classified into 19 categories in biological processes, followed by 10 and eight categories in cellular components and molecular functions, respectively. Greater percentages of these targets were considered to be involved in metabolic (33.76 %), cellular (32.01 %), single-organism (14.02 %), response to stimulus (12.38 %) and developmental (8.64 %) processes. Among cellular component categories, cell (32.36 %) and cell part (32.36 %) were the main functional groups. For molecular function, the binding (34.58 %) and catalytic activity (24.42 %) terms contained the largest numbers of targets. The results may indicate the importance of these miRNAs in gene regulation during pollen development in soybean.

### Expression of miRNA targets in NJCMS1A and NJCMS1B

**Fig. 6:**
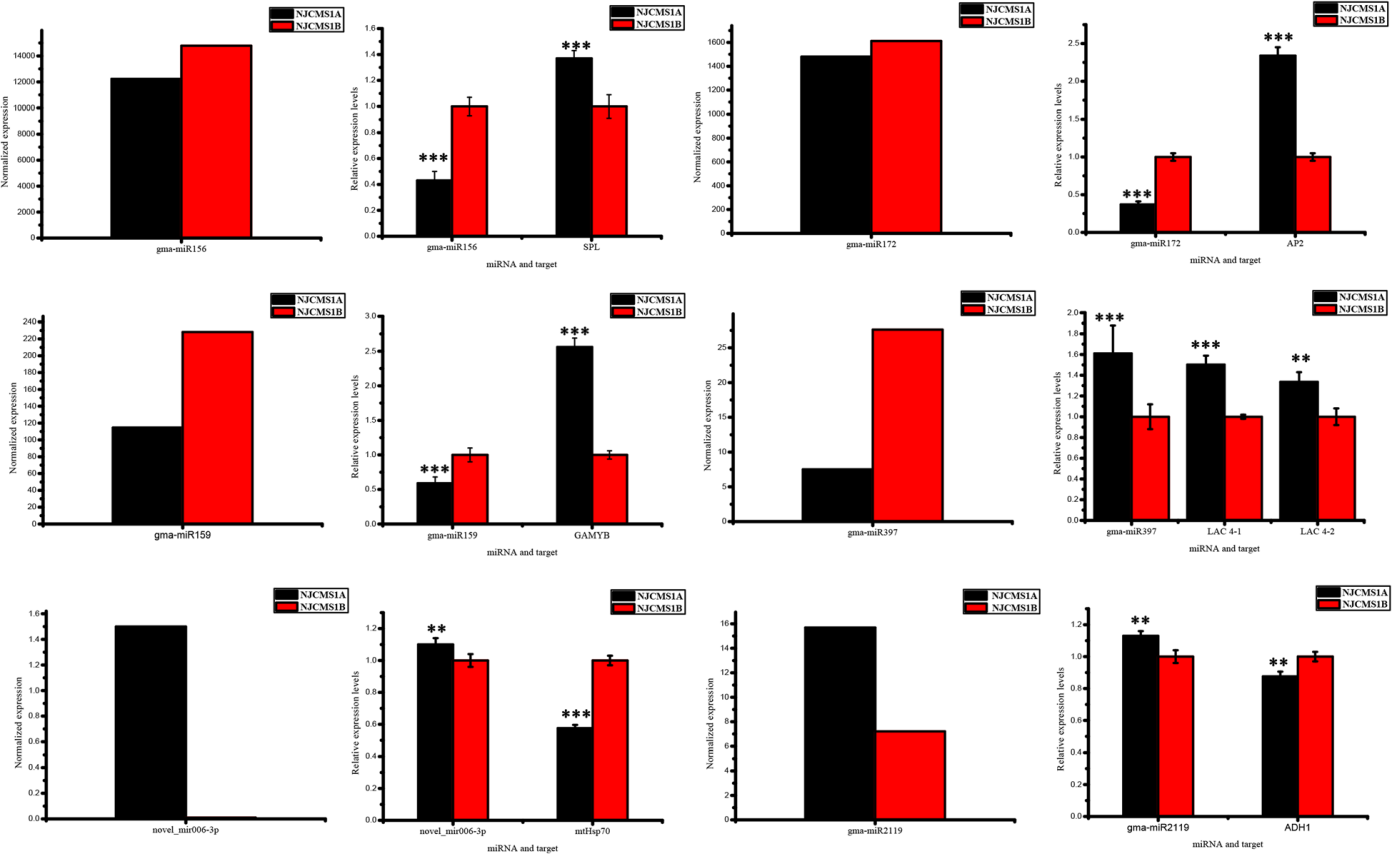
Expression levels of some miRNAs and their target in discussion between NJCMS1A and NJCMS1B. The y-axis indicated the total normalized expression of miRNAs generated from high-throughput sequencing which have the same target. The relative expression levels of miRNA and target were obtained from three biological replicates by qRT-PCR, and the error bars indicated the standard error of the mean of 2^–ΔΔCt^, with NJCMS1B as a control. Expressions that significantly differed (*P* < 0.05) according to Student’s *t*-test were labeled as: “**” and “***” representing *P* < 0.01 and *P* < 0.001, respectively, which indicated extremely significantly differences between NJCMS1A and NJCMS1B. SPL, squamosa promoter-binding protein-like; AP2, APETALA2; LAC 4–1, laccase-4-like1; LAC 4–2, laccase-4-like2; mtHsp70, heat shock 70 kDa protein, mitochondrial; ADH1, alcohol dehydrogenase 1

To test if any inverse relationship between target expression and the level of corresponding miRNA existed, the expression patterns, based on qRT-PCR, of 14 miRNA targets discussed below were studied. As expected, the expression levels of squamosa promoter-binding protein-like, GAMYB, APETALA2, LACs were higher in NJCMS1A than in NJCMS1B, and those of the other 10 targets such as MADS-box protein etc. were lower in NJCMS1A than in NJCMS1B, which were in contrast with their corresponding miRNAs (Fig. 6 and Additional file 2: Figure S8).

## Discussion

### Diverse miRNAs and their characteristics in flower buds of NJCMS1A and NJCMS1B

Small RNAs have been identified as pivotal regulators during anther and pollen development in plants [34–36]. However, no existing studies have reported the relationship between miRNAs and pollen in soybean. To identify miRNAs linked to the regulation of pollen development in soybean, two small RNA libraries were constructed with the flower buds of a CMS line NJCMS1A and its maintainer NJCMS1B. Based on high-throughput sequencing, we identified a much wider range of small RNAs, 18–30 nt in length (Fig. 1), with 24 nt being the most abundant, followed by 21 and then 22 nt. The results were consistent with previous reports in various other species, including *Arabidopsis thaliana* [37], *Citrus sativus* [38], *Medicago truncatula* [39], *Oryza sativa* [40] and *Zea mays* [41], suggesting that the most abundant small RNA length is 24 nt. Most known miRNAs had the canonical 21 nt length (Additional file 2: Figure S1), suggesting they were Dicer-like1 cleavage products.

The identification of miRNAs has previously been reported in male-sterile plants, including maize, rice, *Brassica campestris* ssp*. chinensis*, *Brassica juncea* and cotton [23–28]. The present study is the first to report the comprehensive identification of miRNAs and their targets using deep sequencing of flower buds between CMS and maintainer lines in soybean. Among the 560 known miRNAs, 205 had additional nucleotides in the 5′ or 3′ terminus, which was caused by an imprecise or alternative cleavage by Dicer during pre-miRNA processing [42]. According to miRBase release 21.0, 135 mature miRNAs (31.62 %, 427 mature sequences in total) were annotated as high confidence in *Arabidopsis thaliana*, and all of those miRNAs were reported to have their miRNA-5p or miRNA-3p pairing accumulated. However, until now, no miRNAs were annotated as high confidence in soybean. Thus, the identification of novel miRNAs on the other arm of known pre-miRNAs increased the confidence of these miRNAs. With the criteria described by Kozomara et al. [33] and miRBase 21.0, 156 known miRNAs and four novel miRNAs (Additional file 1: Table S7) could be annotated as high-confidence miRNAs.

Considering that some miRNAs are widespread, while others are distributed in limited plant species, all of the known miRNAs have been classified into four categories: “highly conserved”, “moderately conserved”, “lowly conserved” and “non-conserved”. In our study, a high proportion of non-conserved miRNA families (80.63 %) were identified, and only 27 highly conserved miRNA families were identified (Additional file 1: Table S4). Meanwhile, 158 novel miRNAs, which had not been reported in soybean before, were detected. Novel miRNAs are considered to be young miRNAs that evolved recently, and are expressed weakly. They are likely to be species-specific miRNAs, which are classified as non-conserved miRNAs, as reported from other plants, such as *Arabidopsis thaliana* [37]. Unlike conserved miRNAs, only a small percent of soybean novel miRNAs (37.74 %) were encoded by multiple loci, which was in accordance with the previous results [43].

Previous studies have demonstrated that many miRNAs exist in male-sterile plants. Shen et al. [23] isolated seven novel miRNA families and one known miRNA from a mixture of anthers from a maize CMS line and its maintainer. In 2013, 100 known miRNAs and 81 novel miRNAs of pollen miRNAs from a maize S type CMS line and its fertility-restored line were identified by Yu et al. [27], with nine of the known miRNAs having significant expression differences between the two lines. In the same year, Yang et al. [26] identified 197 known and 78 new candidate miRNAs during the reproductive development of *Brassica juncea*, and discovered 47 differentially expressed miRNAs between the CMS and its maintainer. Based on high-throughput sequencing, Wei et al. [28] compared the expression patterns of miRNAs during cotton anther development between the genetic male-sterile mutant and its wild type. A total of 16 conserved miRNA families were identified; six of them were significantly differentially expressed [28]. Recently, Jiang et al. [25] constructed two small RNA libraries from the flower buds of male-sterile and male fertile lines in *Brassica campestris* ssp. *chinensis*, and 18 differentially expressed miRNAs with more than two-fold relative changes were identified. In our study, 560 known miRNAs, 105 novel miRNA-3p/miRNA-5p of known pre-miRNAs, 23 new miRNA members and 159 novel miRNAs (Table 2) in the soybean flower buds of the NJCMS1A and NJCMS1B were identified. We speculate that these miRNAs may participate in the complex network regulating the pollen development process of soybean.

### Degradome analysis of the interactions of miRNAs and their targets

miRNAs regulate genes by mediating gene expression at the post-transcriptional level in plants mainly through mRNA cleavage at certain sites [44]. Currently, bioinformatics-based calculations are commonly used to predict miRNA targets. In recent years, modified 5′ RACE has been frequently used to demonstrate miRNA targets in pollen development [36]. The confirmation procedures were convincing but not effective in identifying a number of candidate targets. Fortunately, a high-throughput method combining 5′ RACE with next-generation sequencing technology was developed, which has become the new method for identifying targets [45].

With our degradome analysis, 859 targets were chosen and predicted to be cleaved by 297 miRNAs (Additional file 1: Table S9). Among the identified targets of miRNAs, some were previously shown to be involved in floral organ or anther development, such as squamosa promoter-binding protein-like [33], APETALA [46], GAMYB or GAMYB-like genes [47], GRAS gene family [48], and auxin response factor [49–51]. The expression levels of squamosa promoter-binding protein-like, APETALA2, GAMYB or GAMYB-like genes and their miRNAs were compared by qRT-PCR (Fig. 6), which showed the inverse relationship between target gene expression and the level of corresponding miRNA. A set of PPR proteins are targeted by gma-miR156, gma-miR160, gma-miR319a-5p, gma-miR4413, gma-miR5674, gma-miR5770 and N-gma-miR164r-3p, which are a group of RNA-binding proteins classified as one of the most important restorer gene families in plants [3]. The transcription of PPR proteins is likely to be inhibited in pollen, so we speculated that these types of miRNAs have correlations with restorer genes that lead to fertility restoration. Some of the targets have not been shown to be associated with pollen development as miRNA targets, such as homeobox-leucine zipper protein and no apical meristem protein. Chen et al. [3] indicated that several models, such as the MADS-box transcription factor in the retrograde regulation model, programmed cell death (PCD) and reactive oxygen species (ROS) in the aberrant PCD model, and ATP and NADH in the energy deficiency model etc. involved mechanisms that may lead to male sterility in plants.

### Targets of miRNAs with retrograde regulation and PCD

Three targets of gma-miR156b and gma-miR156f are members of the MADS-box transcription factor family, which participate in reproductive developmental control and flower development in plants [52, 53]. MADS-box genes are differentially expressed during early germination of the male gametophyte in *Nicotiana tabacum* [54] and are distinct in the male organ primordia of *Arabidopsis thaliana* [55]. In maize, their products accumulate in apoptotic bodies during anther dehiscence [56]. Also, MADS-box genes may be in accord with CMS, because in carrot, mitochondria affect the expression of MADS-box genes homologous to GLOBOSA and DEFICIENS, which led to stamens being replaced by carpels, becoming the “carpeloid” CMS type [57]. Yang et al. [58] found that MADS-box genes were associated with cytoplasmic homeosis in CMS stem mustards, by specifically inhibiting the mitochondrial electron transport chain of *Brassica juncea*. Recently, Huang et al. [59] found a MADS-box transcription factor in soybean using a microarray analysis, and its constitutive expression in tobacco caused sterility because of shortened and curly stalks, and the failure of pollen release from the anthers. However, it was only targeted by gma-miR156b and gma-miR156f in NJCMS1A and was down-regulated (Additional file 2: Figure S8). Even so, we speculated it was highly expressed during the pollen development stage, which would lead to pollen abortion. We may be able to control the expression of gma-miR156b and gma-miR156f to regulate male sterility, therefore the relationship between gma-miR156b and gma-miR156f, and male sterility of the soybean CMS line, needs further research.

The degradome analysis showed that LAC was targeted by gma-miR397a and gma-miR397b-5p. Interestingly, LAC was only targeted by gma-miR397 in NJCMS1B, and the LAC expression level was higher in NJCMS1A than in NJCMS1B (Fig. 6), which may have been caused by the lower expression of gma-miR397 (Additional file 1: Table S8-2) and the negative regulation of LAC expression in NJCMS1A. Some studies have indicated that LAC participates in brassinosteroid-regulated plant growth and that overexpressed OsLAC could lead to a semi-sterile phenotype [60]. Brassinosteroids are essential for male fertility in plants, and mutations in brassinosteroid biosynthesis lead to reduced pollen levels, viability, and release efficiencies [61]. Thus, we speculated that gma-miR397 and LAC have some relationship with CMS. However, qRT-PCR showed that gma-miR397 was more highly expressed in NJCMS1A (Figs. 3 and 4), which was inconsistent with the sequencing results and probably related to the anther’s developmental period. In maize, miR397 was more highly expressed in the tetrad stage and then rapidly declined in the mononuclear stage [23]. Thus, the relationship of miR397 and LAC with CMS requires further studies.

Novel_mir_006-3p was a novel miRNA found to target the mitochondrial heat shock 70 kDa protein (mtHsp70), which influences heat- and H_2_O_2_-induced PCD in plants [62]. Plant PCD, triggered by the retrograde signals from the release of cytochrome c from mitochondria into the cytosol and the overproduction of ROS, controls cellular degeneration of the tapetum and leads to male sterility [59]. mtHsp70 functions as a motor and is required for polypeptide translocation across the mitochondrial inner membrane subsequent to protein folding reactions in the matrix [63]. In animals, Hsp70-2 participates in synaptonemal complex functions during meiosis in male germ cells and results in male infertility in mice [64]. The results of Chen et al. [65] indicate that Hsp70 has a close relationship with the CMS of sorghum. A lack of Hsp70 expression leads to functional anomalies and decreases the numbers of mitochondria, which can eventually lead to pollen sterility [65]. The novel_mir_006-3p was only found in CMS, and mtHsp70 was down-regulated in NJCMS1A (Fig. 6). However, whether opposed expression levels of novel_mir_006-3p and its target gene in the CMS anthers leads to a significant increase in PCD remains to be studied. In addition, a heat shock cognate 70 kDa protein-like gene that was only targeted by gma-miR393a showed a down-regulated expression level in NJCMS1A (Additional file 2: Figure S8), which may also be related to male sterility.

### Targets of miRNAs involved in responses to oxyradical stress

NADP-dependent isocitrate dehydrogenase (NADP-ICDH) and 6-phosphogluconate dehydrogenase were targeted by gma-miR162a, gma-miR162b and gma-miR162c, and gma-miR399d, gma-miR399e, gma-miR399f and gma-miR399g, respectively. Both are NADPH-generating dehydrogenases in plants, and NADPH is a key cofactor in the cellular redox homeostasis and necessary in the metabolism of ROS [66]. In *Arabidopsis thaliana*, NADP-ICDH activity is regulated by molecules involved in ROS, including H_2_O_2_ [67]. These two types of miRNAs had no significant differences in expression levels between the NJCMS1A and NJCMS1B. Surprisingly, they were only targeted in NJCMS1A and the qRT-PCR results show that NADP-ICDH and 6-phosphogluconate dehydrogenase were down-regulated (Additional file 2: Figure S8). The decreased NADPH level regulated by miRNA in the NJCMS1A may lead to a transient oxidative burst and significant ROS accumulation. However, more studies are needed to understand the relationship between miRNA targeting and ROS that may lead to male sterility in soybean.

Alcohol dehydrogenase 1 (adh1) was the only target of gma-miR2119 which encoding an important enzyme during the conversion of acetaldehyde into ethanol, and regenerating NAD^+^ in the process [68]. In higher plants, the adh gene expression increases dramatically in response to hypoxia [68]. Garabagi et al. [68] showed that the adh1 expressed in the plants appears to be restricted to immature pollen grains. Hajós-Novák et al.’s results revealed that reduced ADH enzyme activity in the pollen grains would affect the gametophyte [69]. Both sequencing and qRT-PCR indicated that gma-miR2119 expression was up-regulated in NJCMS1A. In contrast, adh1 expression was down-regulated in NJCMS1A (Fig. 6). This may indicate that gma-miR2119 and adh1 are related to pollen development, and thus further research is needed.

### Targets of miRNAs in energy metabolism

NADH-ubiquinone oxidoreductase 24 kDa subunit was identified as the target of gma-miR156b and gma-miR156f, and has a regulatory role in the complex I function in the electron transport chain, which transfers electrons from NADH to ubiquinone through the intramolecular redox centers (FMN and Fe-S centers) in mitochondria [70]. Dihydrolipoyl dehydrogenase (E3) is an important part of the pyruvate dehydrogenase complex, and it was the target of gma-miR169t. Selinski et al. [71] demonstrated that the ATP/NAD(P)H ratio (by malate valves and NAD^+^ biosynthesis) contributes to satisfying the energy demand during pollen development. In this process, the pyruvate dehydrogenase complex is the critical pathway that supports energy generation for pollen and pollen tube growth [71].

The mitochondrial-like ATP synthase subunit beta was targeted by gma-miR1521a. It is one part of the F-ATPases (F1F0-ATPases), which are located in mitochondrial plasma membranes and are the prime producers of ATP, using the proton gradient generated by oxidative phosphorylation [72, 73]. One of the targets of gma-miR172c, gma-miR172d and gma-miR172e was the mitochondrial transcription termination factor family protein (mTERFs; homolog of AT4G09620). It was predominantly encoded by the nuclear genome and imported into mitochondria after protein synthesis to play important roles in regulating the organellar transcription machinery [74]. In *Arabidopsis thaliana*, mutated mTERF15 led to defective mitochondrial functioning and significantly disturbed normal plant vegetative to reproductive growth [74]. These represent core parts of ATPases or mitochondria that produce ATP for pollen development and miRNA biogenesis [26]. These types of genes were only targeted by miRNA (or the target site matched the filter *P* < 0.05) in NJCMS1A, indicating that there is a close connection between ATP and CMS. qRT-PCR showed that these targets were down-regulated in NJCMS1A (Additional file 2: Figure S8). However, whether an insufficient supply of ATP may lead to male sterility still needs to be researched. The identification of miRNAs and their targets indicates that there are relationships between miRNAs and pollen development. In addition, some miRNA interactions and target gene expressions may only function during a certain period. Thus, in the future we will research the expression of some important miRNAs and their genes in a specific time period to understand the underlying mechanisms that lead to male sterility in the CMS line of soybean.

## Conclusion

In this study, a large number of miRNAs were identified during pollen development in the soybean CMS line NJCMS1A and its maintainer NJCMS1B, by deep sequencing, including 105 novel miRNAs on the other arm of known pre-miRNAs, 23 new miRNA members of known miRNAs, 158 novel miRNAs and 160 high-confidence miRNAs. Additionally, a degradome analysis was used to identify the targets of the discovered miRNAs. Through the expression of miRNAs associated with their targets, we found that there were interactions between miRNAs and their targets. These results revealed that the miRNAs might participate in flower and pollen development, which would provide valuable clues for exploring miRNA-mediated regulatory networks in soybean CMS lines.

## Methods

### Plant materials, sample collection and total RNA extraction

The soybean cytoplasmic male-sterile line NJCMS1A was developed through consecutive backcross procedures with the cultivar N8855 as donor parent and N2899 (designated as NJCMS1B afterwards) as recurrent parent [8, 9, 75, 76]. NJCMS1A and NJCMS1B were planted in the summer of 2013 at Jiangpu Experimental Station, National Center for Soybean Improvement, Nanjing Agricultural University, Nanjing, Jiangsu, China. The male-sterile plants were identified through three kinds of methods including the dehiscence of anthers, germination rate of pollens, and performance of plants at maturity. Cytological observation showed that the male abortion of NJCMS1A occurred mainly at the early binucleate pollen stage [77]. Because it is very difficult to judge the precise development stage of pollen from the appearance of the flower buds in soybean, so during the flowering period, the flower buds of different sizes up to and including the abortion stage were collected and mixed from NJCMS1A and NJCMS1B plants respectively,, then immediately frozen in liquid nitrogen and stored at −80 °C for further use. Total RNA from the flower buds of NJCMS1A and NJCMS1B was extracted using Trizol Reagent (Invitrogen, Carlsbad, CA, USA) according to the manufacturer’s protocol.

### Small RNA sequencing library construction

Two small RNA libraries were constructed and sequenced by the Beijing Genomics Institute (BGI, Shenzhen, Guangdong Province, China) using the Illumina HiSeq 2000 System (TruSeq SBS KIT-HS V3, Illumina, Santa Clara, CA, USA). The general process is as follows: first, polyacrylamide gel electrophoresis (PAGE) purification of the RNA bands corresponding to 18–30-nt size fractionation was performed to recover small RNAs; second, ligation of the 5p and 3p adapters to the RNA occurred in two steps, each followed by PAGE purification; third, RT-PCR amplification was performed to generate a cDNA colony template library and then purified by PAGE; and fourth, the template was analyzed using an Agilent 2100 Bioanalyzer (Agilent RNA 6000 Nano Kit) and qPCR was performed to produce a qualified library for sequencing.

### Bioinformatics analysis of miRNA identification

After sequencing, raw sequencing reads were processed into clean reads by filtering out adaptor contaminants, oversized insertions, low-quality reads, poly A tags and small tags < 18 nt. The small RNA tags were mapped to the soybean genome (ftp://ftp.jgi-psf.org/pub/compgen /phytozome/v9.0/Gmax/assembly/Gmax_189.fa.gz) using SOAP to analyze their expression and distribution on the genome. All small RNA sequences were used to query the RNA sequences for deposited repeats (RepeatMasker output) in GenBank (NCBI GenBank). Rfam (V 11.0) was used to separate the small RNAs that matched rRNA, scRNA, snoRNA, snRNA, tRNA and remove the degraded mRNA fragments (ftp://ftp.jgi-psf.org/pub/compgen/phytozome/v9.0/Gmax/annotation/Gmax_189_gene.gff3.gz) in the small RNA tags that aligned to exons and introns. Next, to identify conserved miRNAs, clean reads were compared with known miRNAs of soybean deposited at miRBase 21.0 (http://www.mirbase.org/). Finally, the other sequences that did not map to known miRNAs and other kinds of small RNA were referred to as unannotated sequences for new miRNA member identification and novel miRNA prediction.

We identified potentially novel miRNAs using MIREAP (https://source-forge.net/projects/ mireap/), and the secondary structure was predicted by the mfold Web server (http://mfold.rna.albany.edu/?q=mfold/RNA-Folding-Form) [78]. For novel miRNAs, the number of mature miRNAs with predicted hairpins must be no fewer than five in the alignment results (if the miRNA-3p and miRNA-5p are simultaneously on the two arms of pre-miRNA secondary structures, then one of the mature miRNAs must be no fewer than five). The criteria used for selecting miRNAs must meet the following characteristics based on Meyers et al. [32], Zhang et al. [79], and Kozomara et al. [33]: (1) The candidate miRNA-5p and miRNA-3p are derived from opposite stem-arms with minimal matched nucleotide pairs exceeding 16 nt and with maximal size differences up to 4 nt; (2) The most abundant reads from each arm of the precursor must pair in the mature miRNA duplex with a 2-nt, 3′ overhang; (3) The number of asymmetric bulges within the miRNA-5p/miRNA-3p duplex must be fewer than one, and the size of the asymmetric bulges must be fewer than two bases; (4) The candidate miRNA precursor must have high negative MFE and MFEI, with MFE < −0.2 kcal/mol/nt and MFEI > 0.85. Then, the sequence is most likely a miRNA.

The frequency of miRNAs in the two libraries was normalized to the expression of transcript per million (TPM; normalized expression = Actual miRNA count × 1 000 000/Total clean read counts). Following normalization, if one of the miRNA gene expressions between the two samples was zero, then it was revised to 0.01, and if the miRNA gene expression of two samples was less than 1, owing to their too low expression levels, then they were not included in the differential expression analysis. The differential expressions of miRNAs were calculated using fold changes and *P*-values from the normalized expression with the selection threshold of |Log_2_FC (Fold Change)| ≥ | ± 1| and *P*-value < 0.05, respectively.

### Degradome library construction, data analysis and target identification

Two degradome libraries were constructed according to the methods by Addo-Quage et al. and German et al. [14, 80]. In brief, poly-A-containing mRNA was purified from total RNA, and then a 5′ RNA adaptor containing a *Mme*I recognition site was ligated to the 5′-phosphate of the poly-A RNA. Subsequently, reverse transcription was performed to generate first-strand cDNA, followed by digestion with *Mme*I and ligation to a 3′ adaptor. Finally, the ligation products were amplified using PCR, gel-purified and subjected to deep sequencing on the Illumina HiSeq 2000 (TruSeq SBS KIT-HS V3, Illumina; BGI, Shenzhen, Guangdong Province, China).

Data filtering was performed to remove adaptor sequences and/or low-quality reads present in the raw reads, resulting in clean reads. This was followed by the collection of 20–21-nt sequences of high quality for subsequent analysis. The degradome tags were mapped to the soybean genome using SOAP and then annotated with rRNA, scRNA, snoRNA, snRNA and tRNA from Rfam and GenBank to separate matched tags from unannotated tags. Degraded sequences with over 70 % single bases in the clean reads were identified as poly-N, which were not used to predict subsequent degradation sites. The statistics of cleaved sites and T-plots were processed by CleaveLand 3.0 (http://axtell-lab-psu.weebly.com/cleaveland.html), and PAREsnip (http://srna-workbench.cmp.uea.ac.uk/tools/paresnip/) was used for the prediction of miRNA targets and hypothesis testing.

The tags mapped to cDNA_sense were used to predict cleavage sites. Categorization of degradome peaks was performed as follows: The height of the degradome peak at each occupied transcript position was placed into one of five possible categories, described below. Category 0: > 1 raw read at the position, abundance at the position is equal to the maximum on the transcript, and there is only one maximum on the transcript; Category 1: > 1 raw read at the position, abundance at the position is equal to the maximum on the transcript, and there is more than one maximum position on the transcript; Category 2: > 1 raw read at the position, abundance at the position is less than maximum but higher than the median for the transcript; Category 3: > 1 raw read at the position, abundance at the position is equal to or less than the median for the transcript; and Category 4: only 1 raw read at the position.

Alignments with scores up to 4.5, where G:U pairs scored 0.5, and no mismatches were found at the site between the 10^th^ and 11^th^ nucleotides of the corresponding miRNAs, were considered potential targets.

### Quantitative real-time PCR validation

Stem-loop qRT-PCR and qRT-PCR were carried out to validate differential expressional levels of miRNAs and miRNA targets, respectively [81]. All primers (Additional file 1: Table S11) were designed based on the mature miRNA and mRNA sequences, and synthesized commercially (Invitrogen, Shanghai, China). According to the procedures provided by the iScript Select cDNA Synthesis Kit (containing GSP enhancer solution, BIO-RAD, USA), 1 μg of total RNA was reverse-transcribed by iScript reverse transcriptase using stem-loop primers. A qRT-PCR analysis was carried out using iTaq Universal SYBR Green Supermix (BIO-RAD, USA) on the Bio-Rad CFX96 machine (CFX96 Touch, BIO-RAD, USA). All reactions were run with three biological replicates, and gma-miR1520d was used as the internal control gene [82]. The miRNA relative expression levels were quantified using the 2^–ΔΔCt^ method. Student’s *t*-test was performed to compare mRNA differences in expression between NJCMS1A and NJCMS1B. The means of 2^–ΔΔCt^ were considered significantly different at *P* < 0.05.

### Online data deposition

All of the small RNA-seq and degradome-seq data were submitted to the National Center for Biotechnology Information (NCBI) under the accession number PRJNA304685.

## Additional files

Additional file 1:
**Table S1.** Summary of small RNA annotations from NJCMS1A and NJCMS1B. **Table S2.** Known miRNAs identified in NJCMS1A and NJCMS1B. **Table S3.** Family member distribution in conserved miRNA families. **Table S4.** Summary of miRNA families found in NJCMS1A and NJCMS1B. **Table S5.** Novel miRNAs on the other arm of known pre-miRNAs. **Table S6.** Novel miRNAs identified in NJCMS1A and NJCMS1B. **Table S7-1.** High-confidence known miRNAs identified in NJCMS1A and NJCMS1B. **Table S7-2.** High-confidence novel miRNAs identified in NJCMS1A and NJCMS1B. **Table S8-1.** The up-regulated miRNAs identified in NJCMS1A and NJCMS1B. **Table S8-2.** The down-regulated miRNAs identified in NJCMS1A and NJCMS1B. **Table S9.** The targets of miRNAs identified in NJCMS1A and NJCMS1B. **Table S10.** Targets of novel miRNAs in NJCMS1A and NJCMS1B. **Table S11.** Primers used in this study. (ZIP 637 kb) Additional file 2:
**Figure S1.** Known miRNA distribution in NJCMS1A and NJCMS1B. **Figure S2.** Family member distribution in conserved miRNA families in NJCMS1A and NJCMS1B. **Figure S3.** Predicted secondary structures of novel miRNAs on the other arm of known pre-miRNAs. **Figure S4.** Predicted secondary structures of new miRNA members. **Figure S5.** Predicted secondary structures of novel miRNAs. **Figure S6.** Predicted secondary structures of high-confidence miRNAs. **Figure S7.** GO analysis of miRNA targets. **Figure S8.** Expression levels of miRNA targets in NJCMS1A and NJCMS1B. (ZIP 4771 kb)

## Abbreviations

CMS: Cytoplasmic male sterility
miRNA: microRNA
MFE: Minimum free energies
MFEI: Minimal folding energy indices
qRT-PCR: Quantitative real-time PCR
SPL: Squamosa promoter-binding protein-like
AP2: APETALA2
PPR: Pentatricopeptide repeat-containing
Hsp70: Heat shock 70 kDa protein
LAC: Laccase
GO: Gene Ontology
PCD: Programmed cell death
ROS: Reactive oxygen species
mtHsp70: Heat shock 70 kDa protein, mitochondrial
NADP-ICDH: NADP-dependent isocitrate dehydrogenase
ADH: Alcohol dehydrogenase
mTERFs: Mitochondrial transcription termination factor family protein
