# Supplementary material for: Identification of miRNAs and their targets by high-throughput sequencing and degradome analysis in cytoplasmic male-sterile line NJCMS1A and its maintainer NJCMS1B of soybean
Source: BMC Genomics. 2016 Jan 5;17:24. doi: 10.1186/s12864-015-2352-0 (PMC4700598; doi:10.1186/s12864-015-2352-0)
Supplement: Additional file 2: — Figure S1. Known miRNA distribution in NJCMS1A and NJCMS1B. Figure S2. Family member distribution in conserved miRNA families in NJCMS1A and NJCMS1B. Figure S3. Predicted secondary structures of novel miRNAs on the other arm of known pre-miRNAs. Figure S4. Predicted secondary structures of new miRNA members. Figure S5. Predicted secondary structures of novel miRNAs. Figure S6. Predicted secondary structures of high-confidence miRNAs. Figure S7. GO analysis of miRNA targets. Figure S8. Expression levels of miRNA targets in NJCMS1A and NJCMS1B. (ZIP 4771 kb) [file 12864_2015_2352_MOESM2_ESM.zip › Figure S4.pdf]

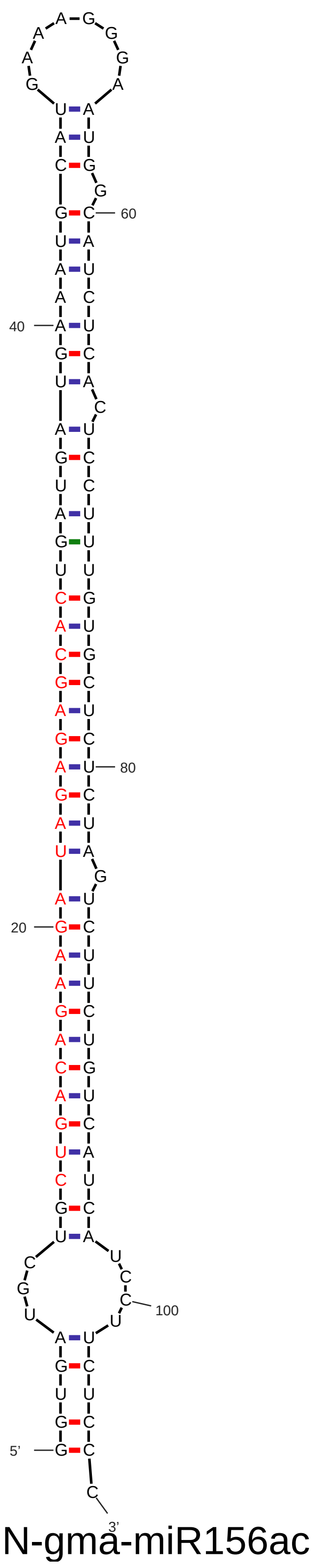

N-gma-miR156ac

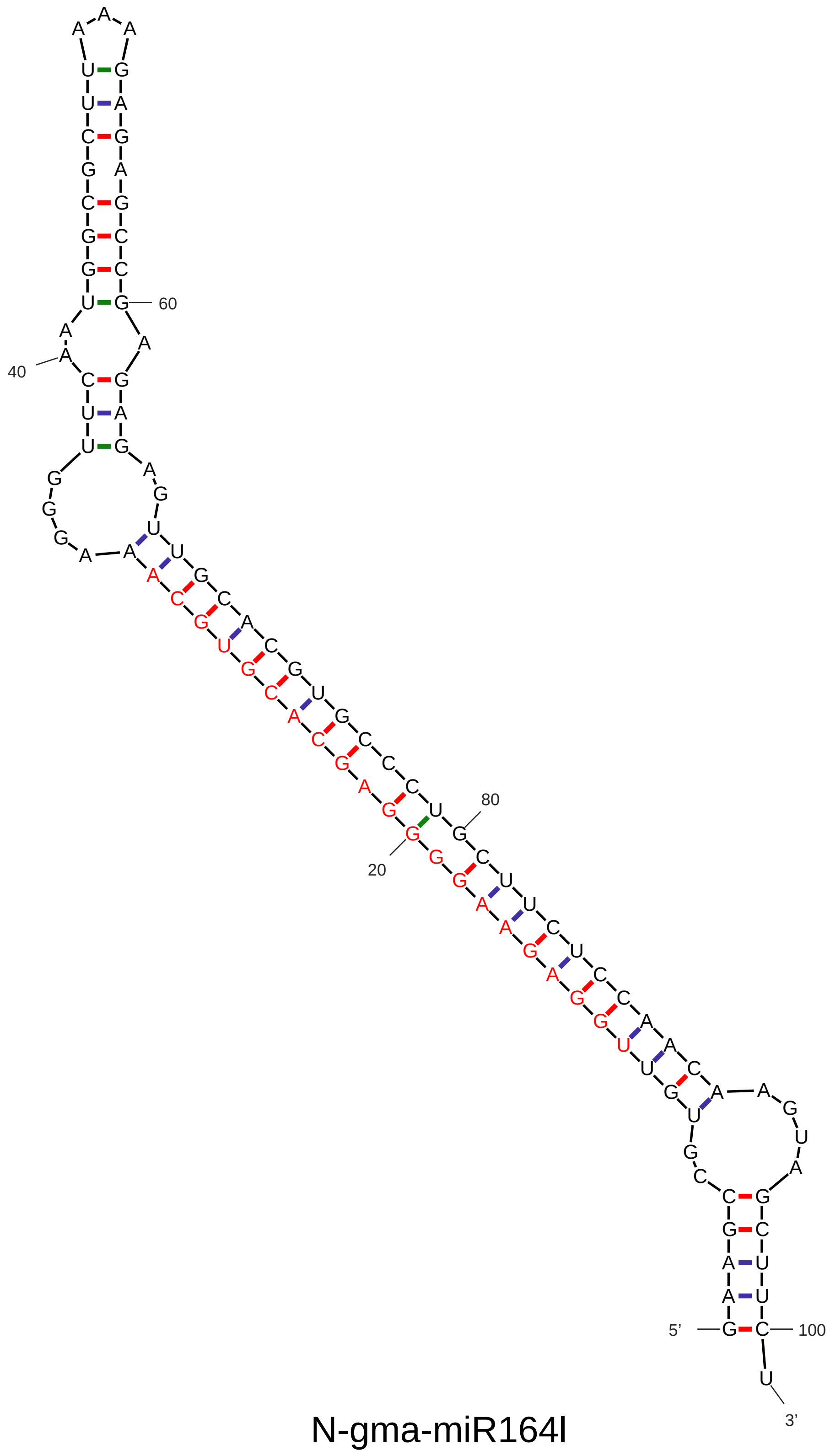

N-gma-miR164l

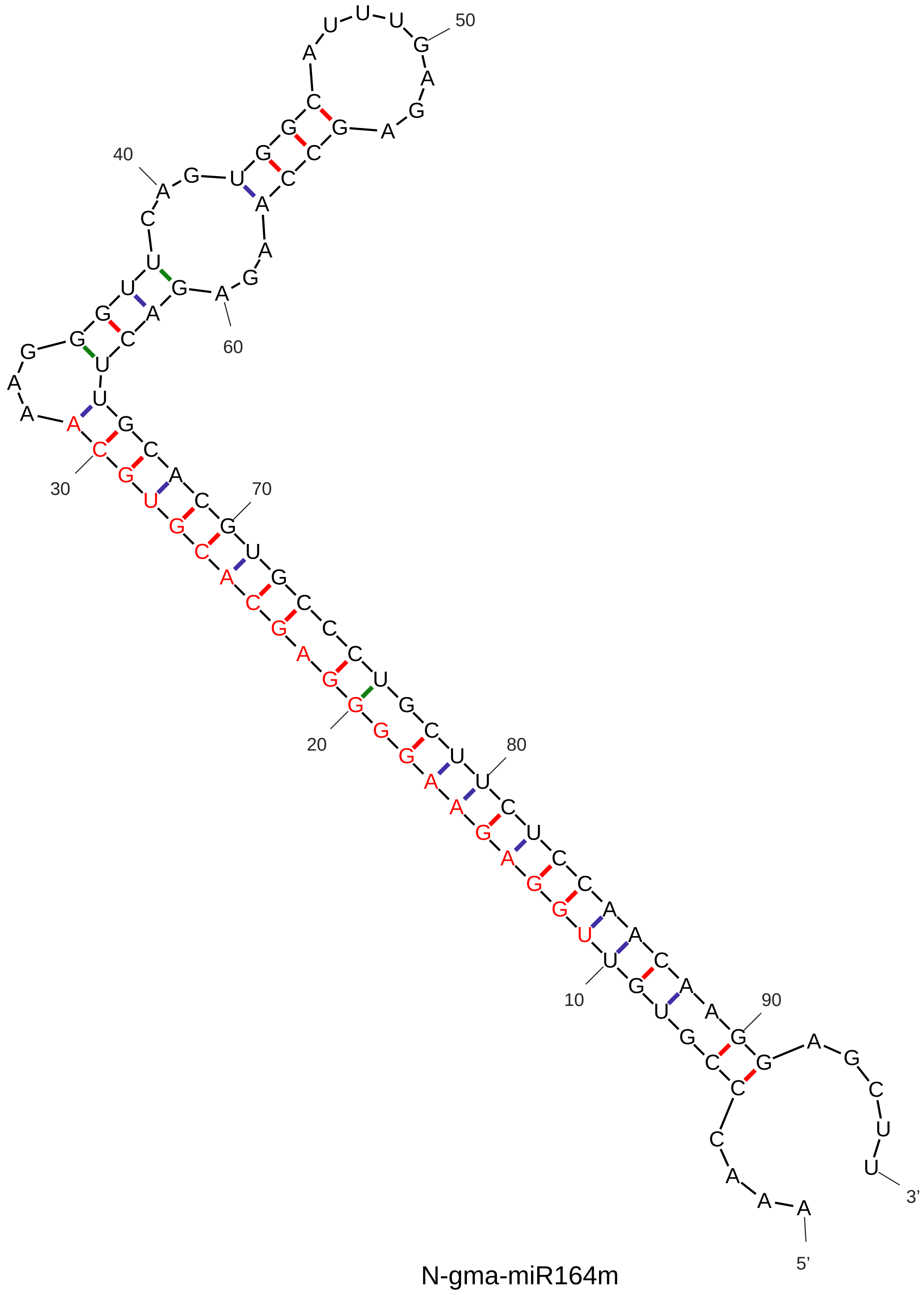

N-gma-miR164m

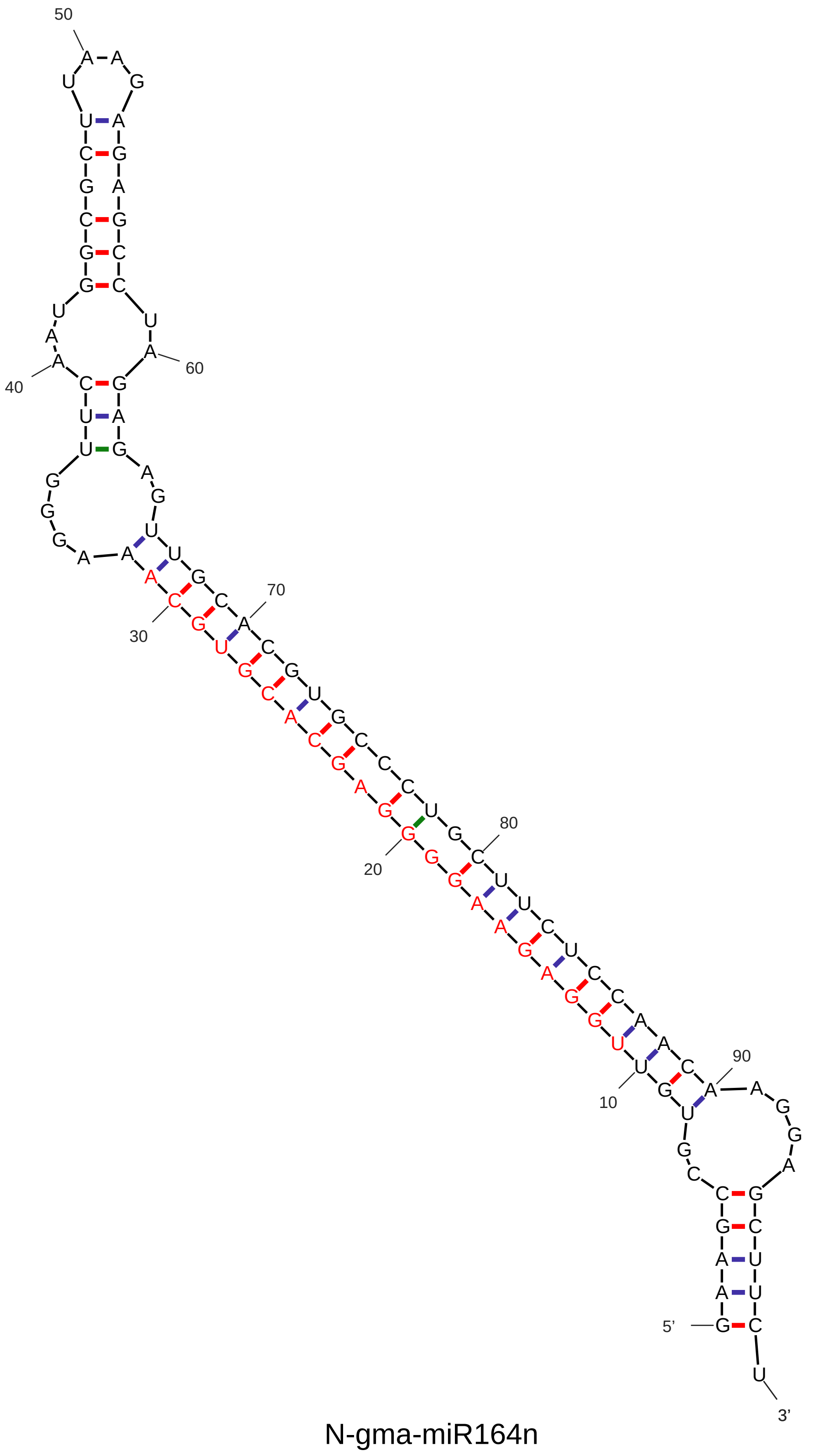

N-gma-miR164n

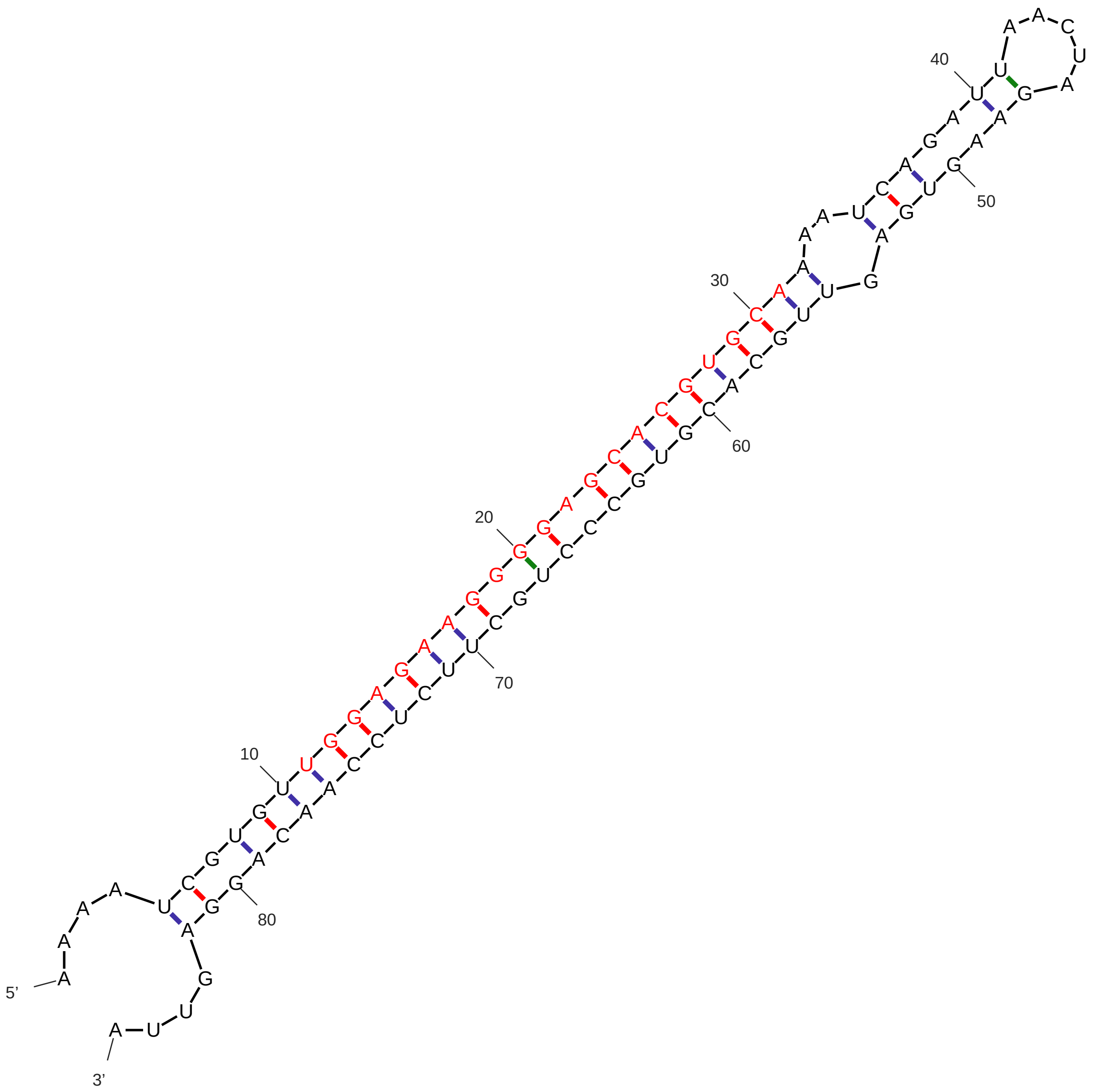

N-gma-miR164o

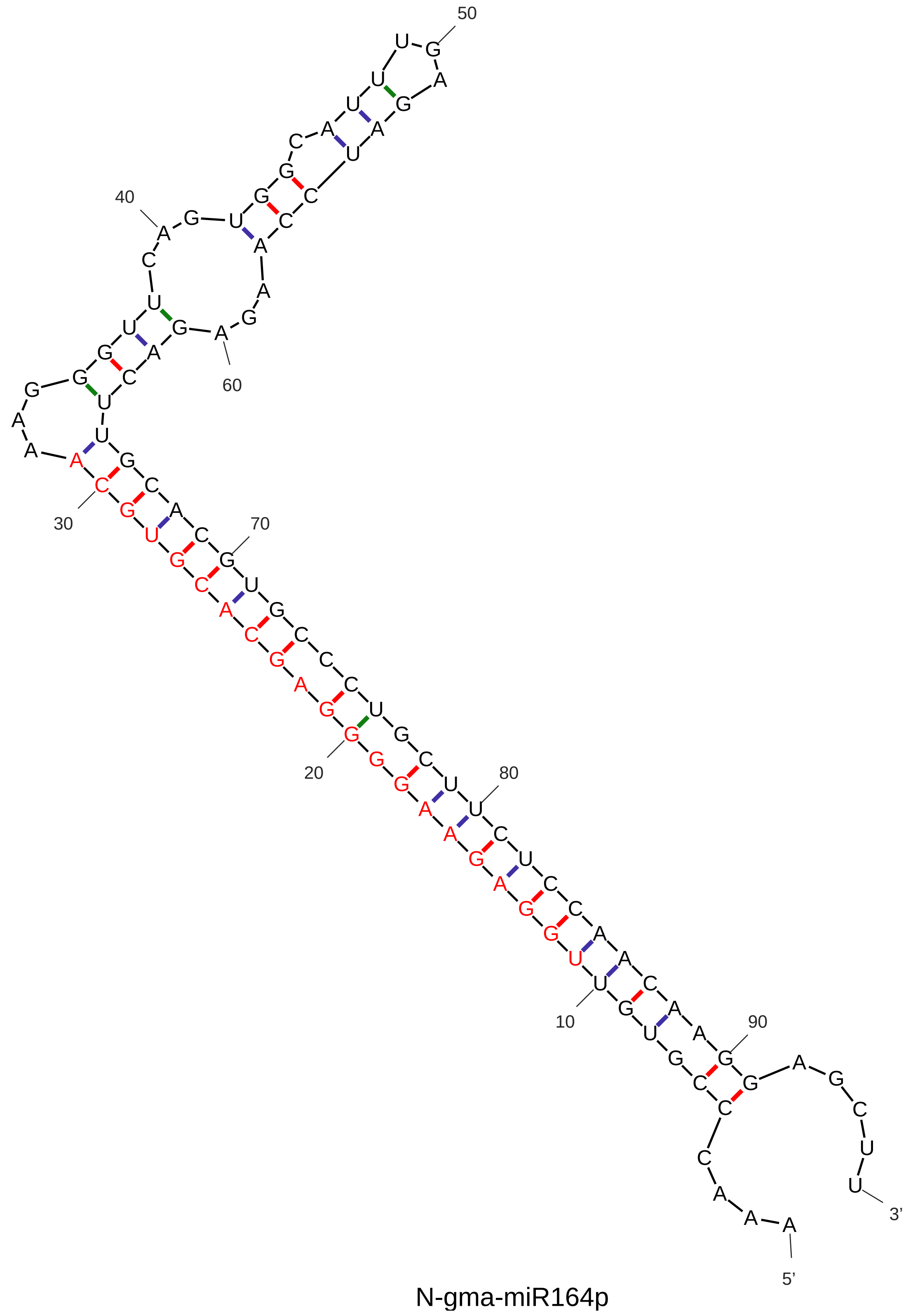

N-gma-miR164p

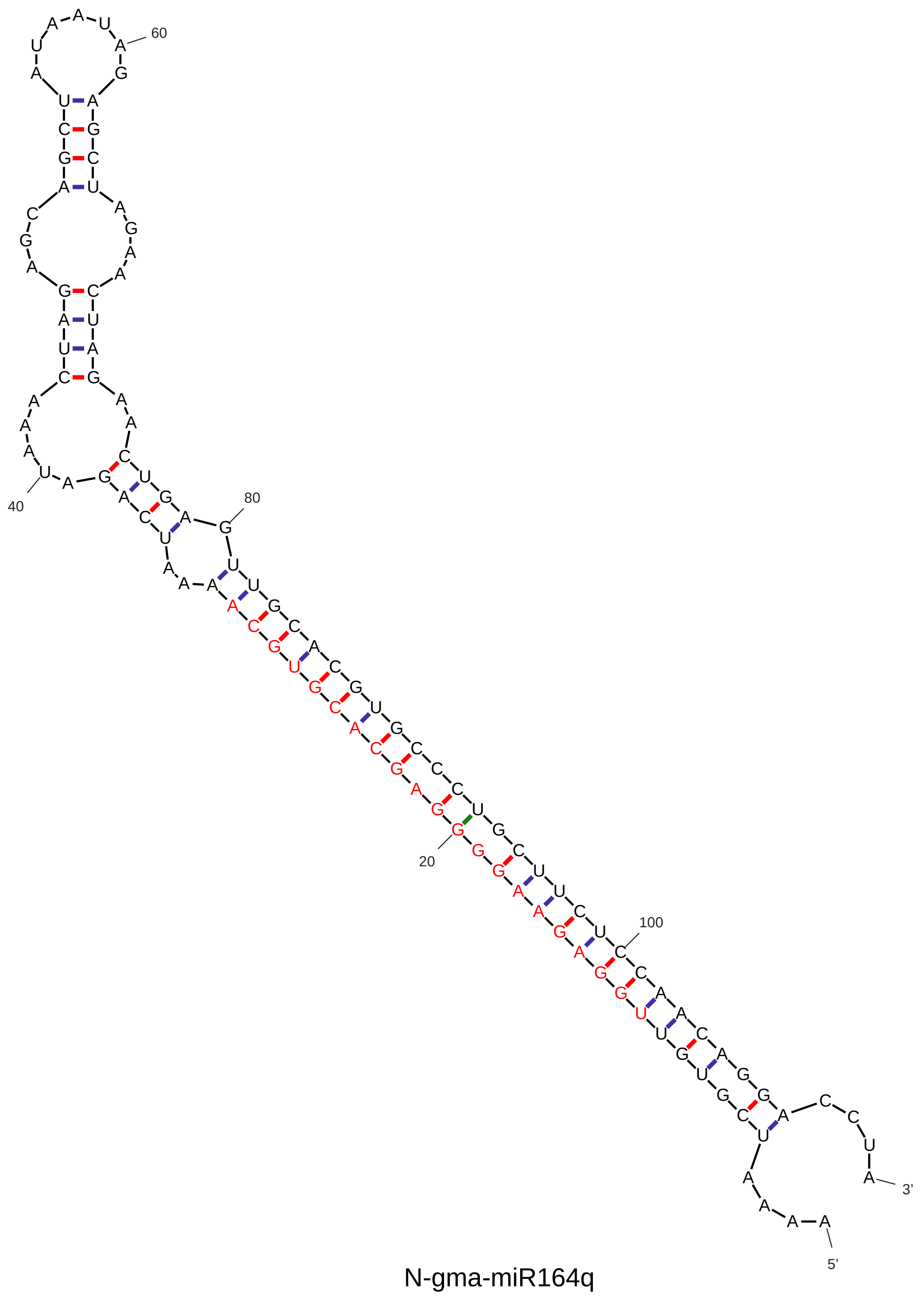

N-gma-miR164q

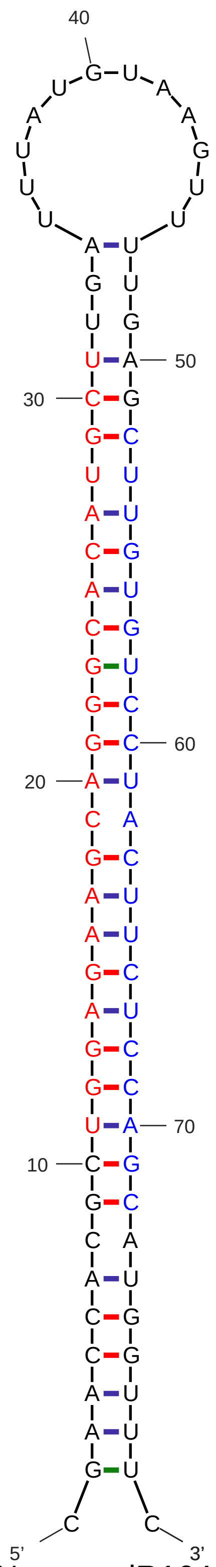

N-gma-miR164r

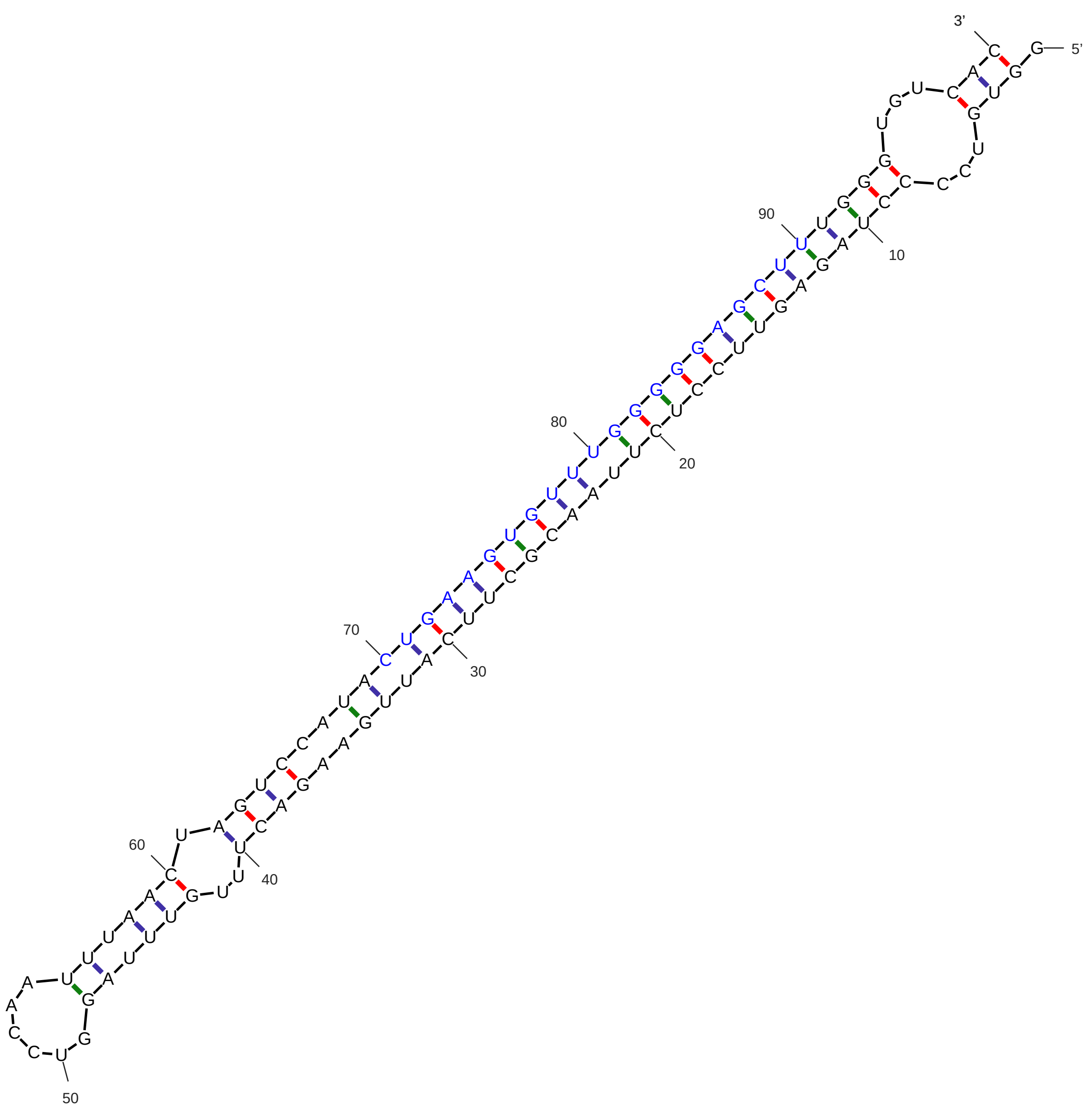

N-gma-miR395n

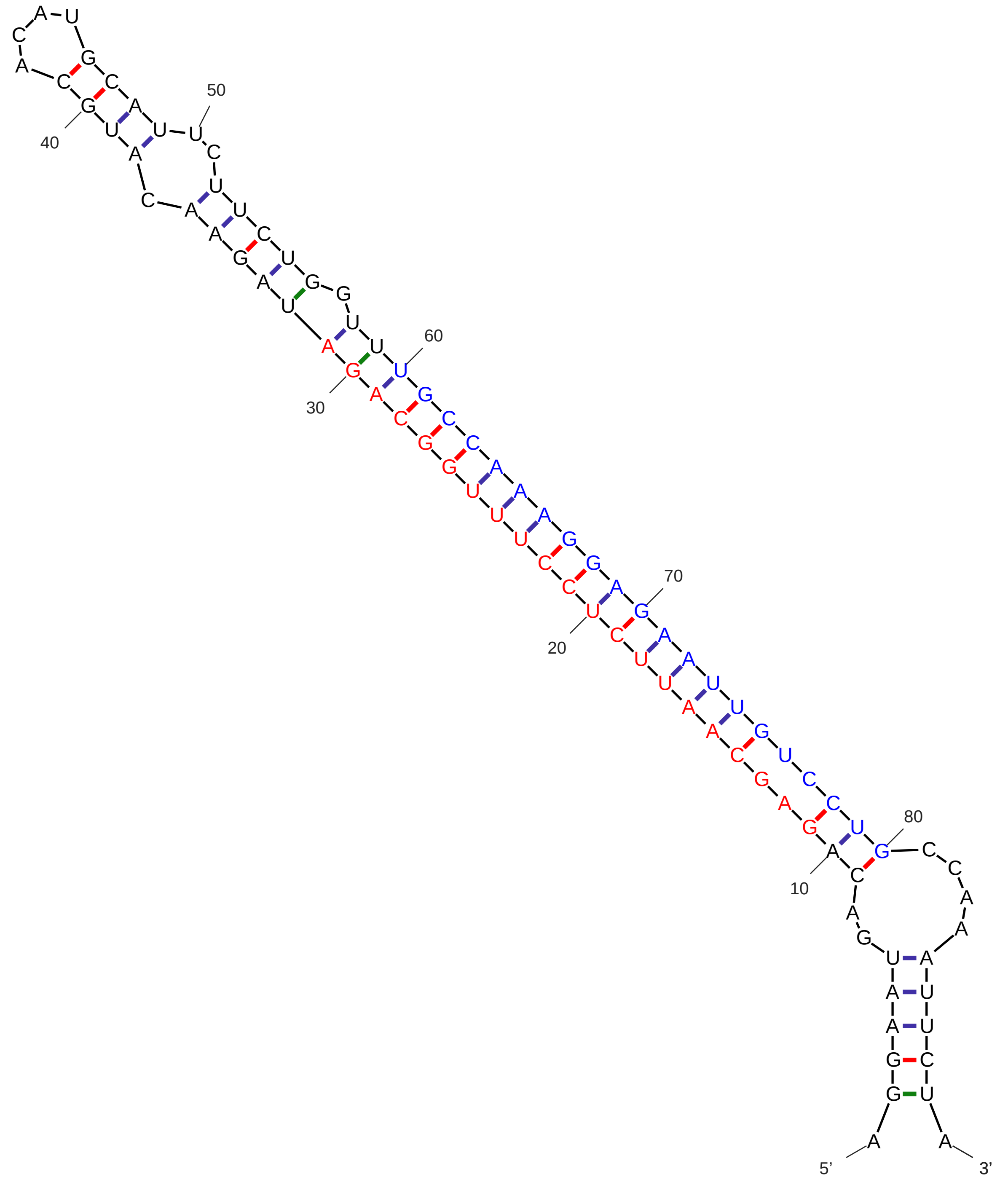

N-gma-miR399i

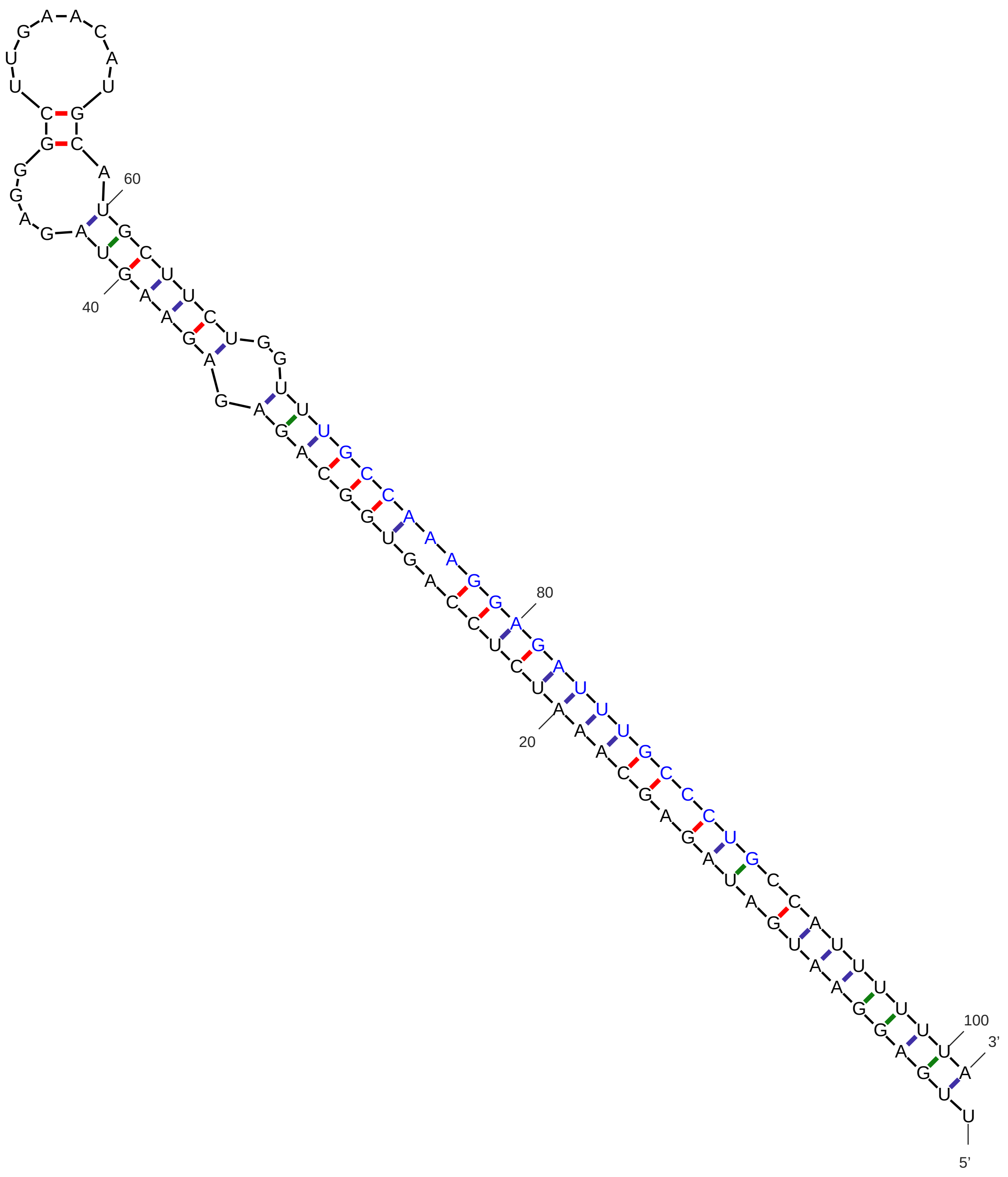

N-gma-miR399j

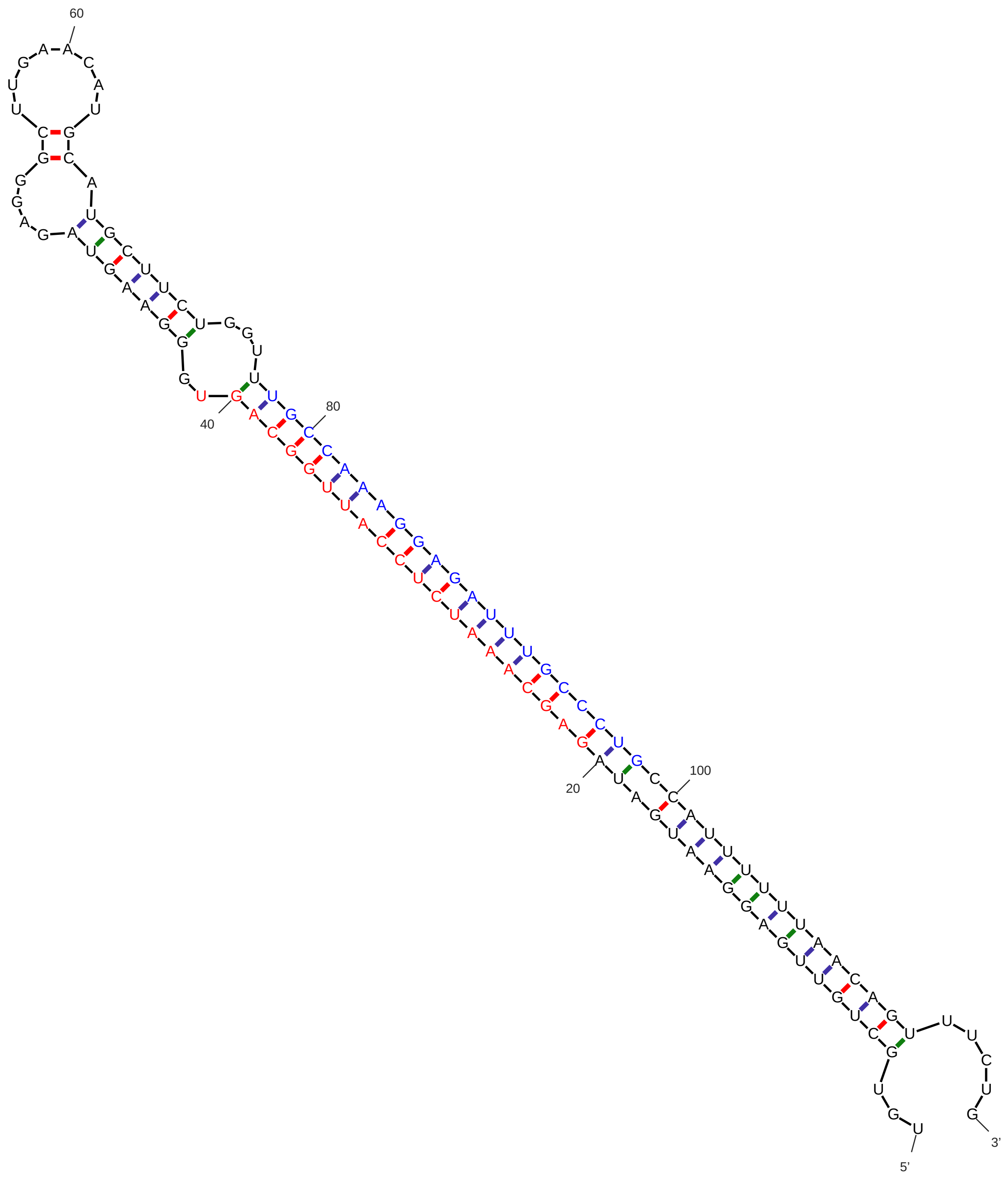

N-gma-miR399k

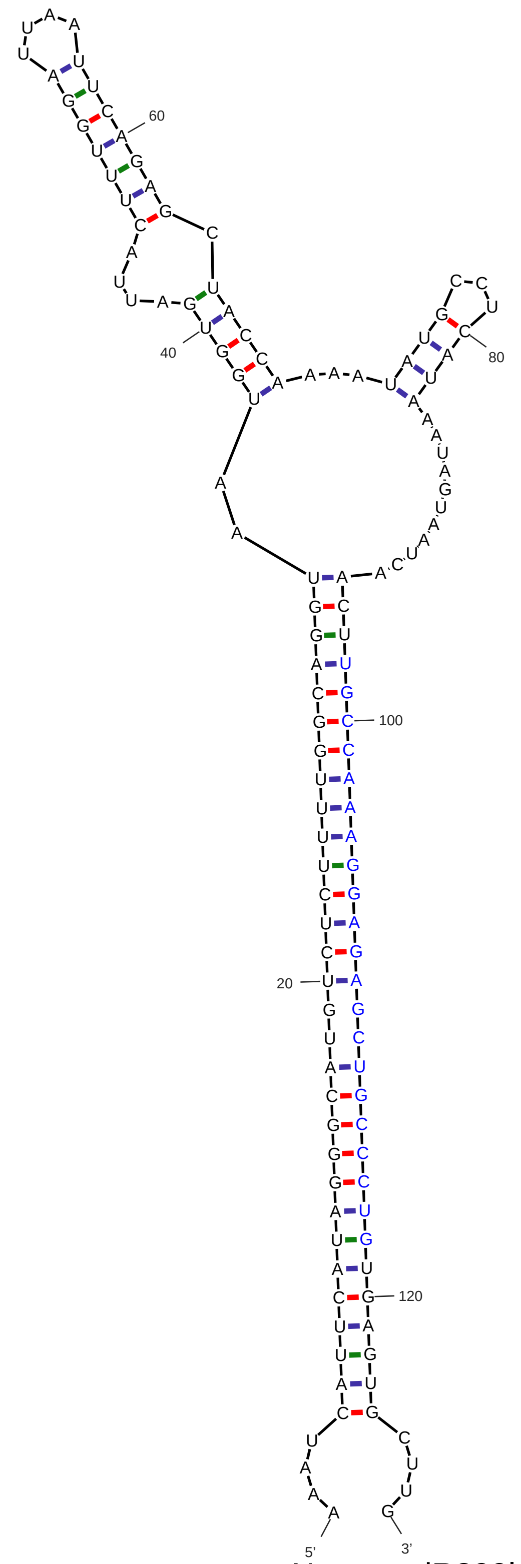

N-gma-miR399l

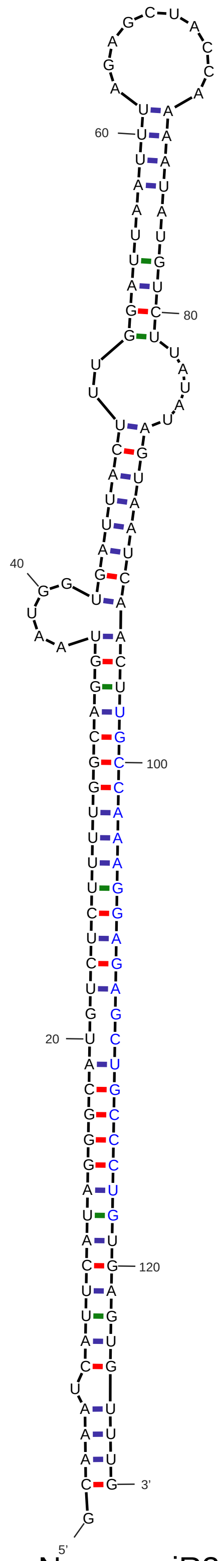

N-gma-miR399m

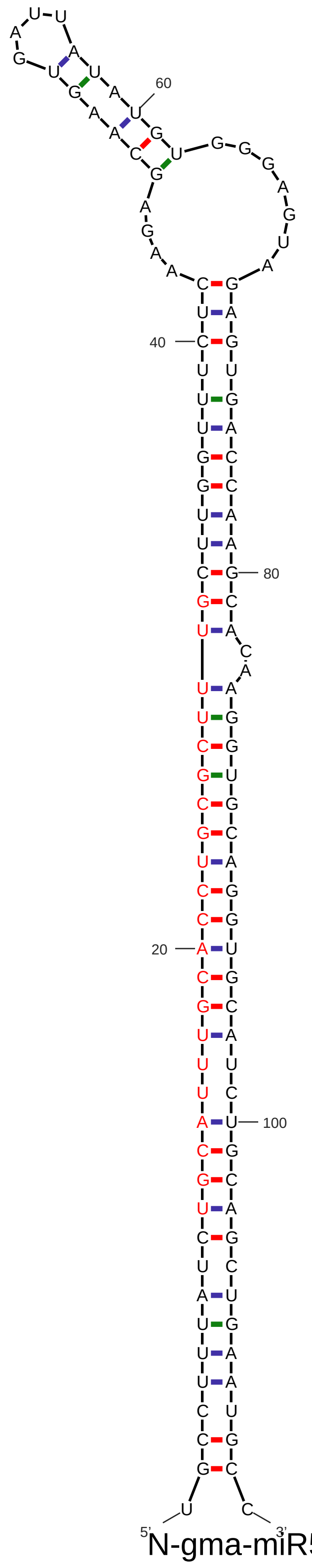

N-gma-miR530f

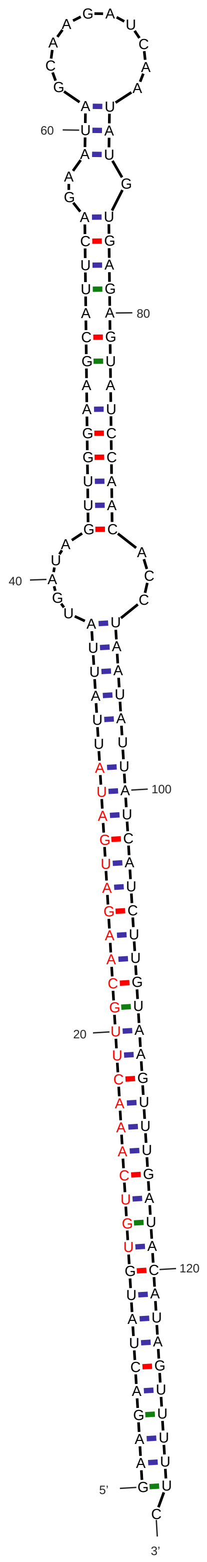

N-gma-miR4348d

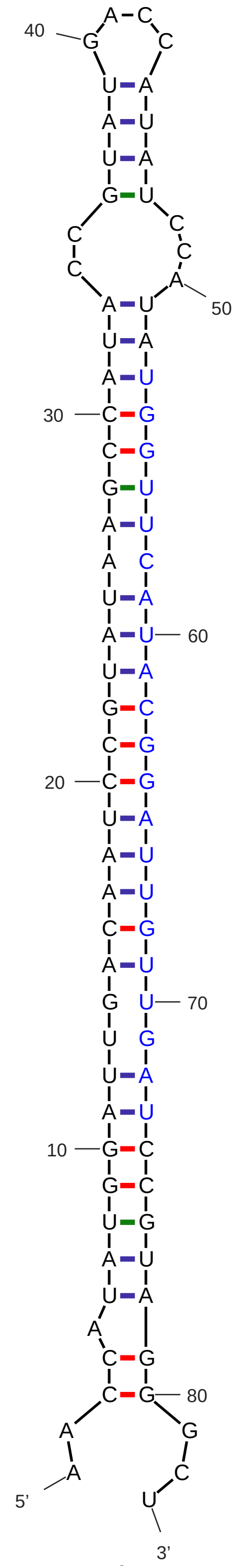

N-gma-miR4380c

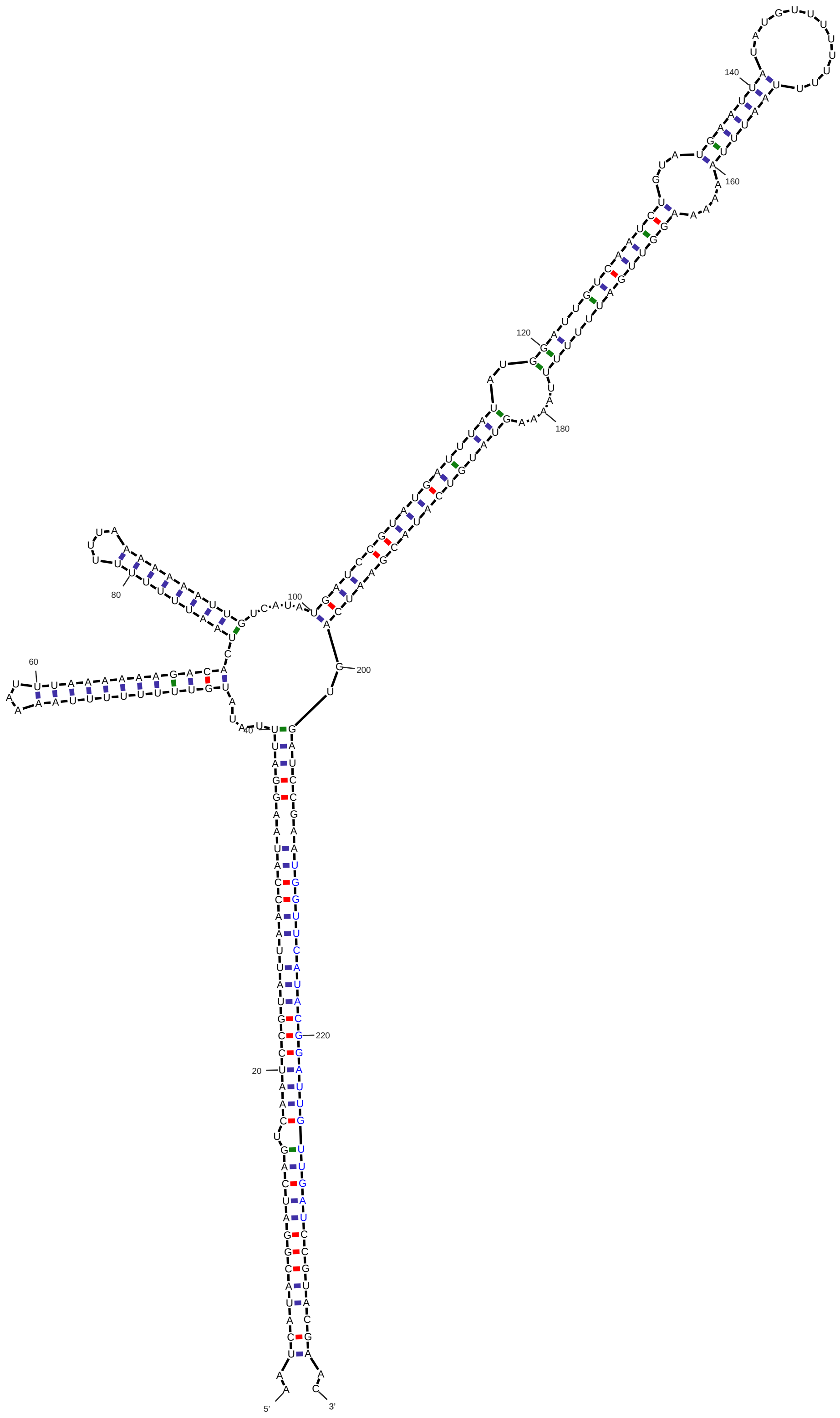

N-gma-miR4380d

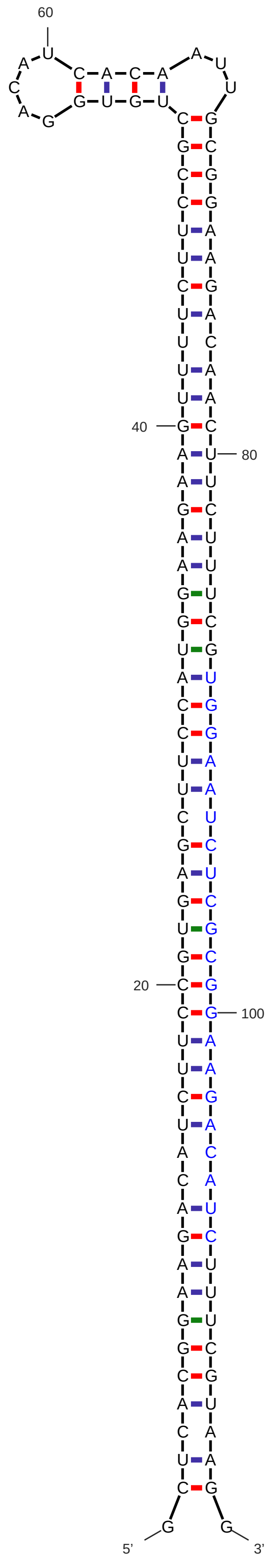

N-gma-miR5673b

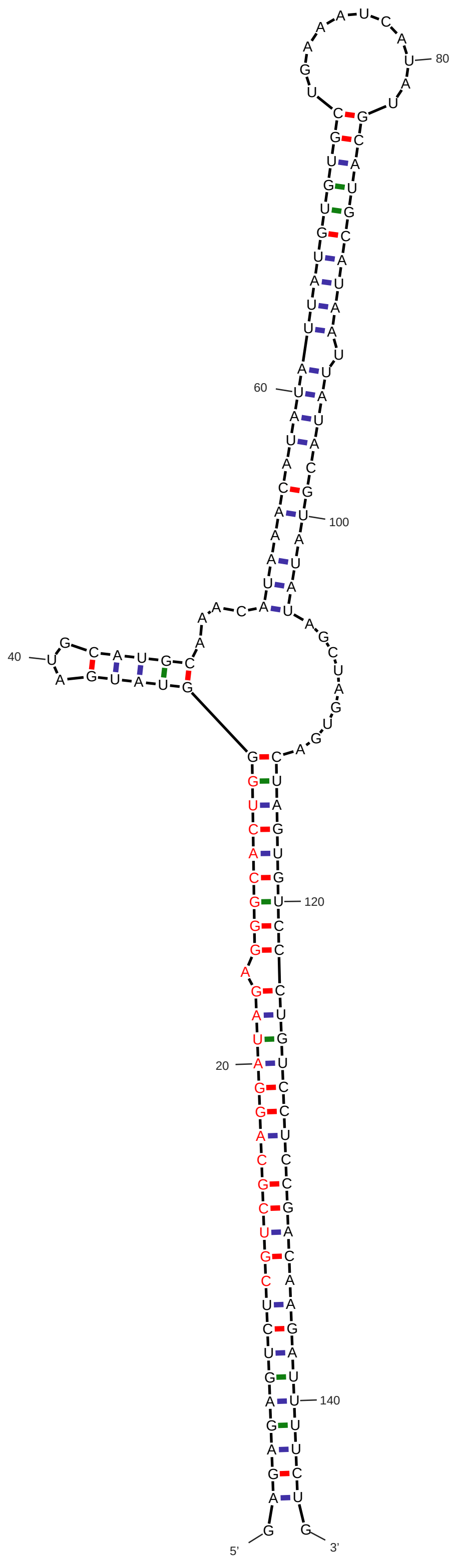

N-gma-miR5786b
