# Supplementary material for: Identification of miRNAs and their targets by high-throughput sequencing and degradome analysis in cytoplasmic male-sterile line NJCMS1A and its maintainer NJCMS1B of soybean
Source: BMC Genomics. 2016 Jan 5;17:24. doi: 10.1186/s12864-015-2352-0 (PMC4700598; doi:10.1186/s12864-015-2352-0)
Supplement: Additional file 2: — Figure S1. Known miRNA distribution in NJCMS1A and NJCMS1B. Figure S2. Family member distribution in conserved miRNA families in NJCMS1A and NJCMS1B. Figure S3. Predicted secondary structures of novel miRNAs on the other arm of known pre-miRNAs. Figure S4. Predicted secondary structures of new miRNA members. Figure S5. Predicted secondary structures of novel miRNAs. Figure S6. Predicted secondary structures of high-confidence miRNAs. Figure S7. GO analysis of miRNA targets. Figure S8. Expression levels of miRNA targets in NJCMS1A and NJCMS1B. (ZIP 4771 kb) [file Figure S6.pdf › Figure S6.pdf]

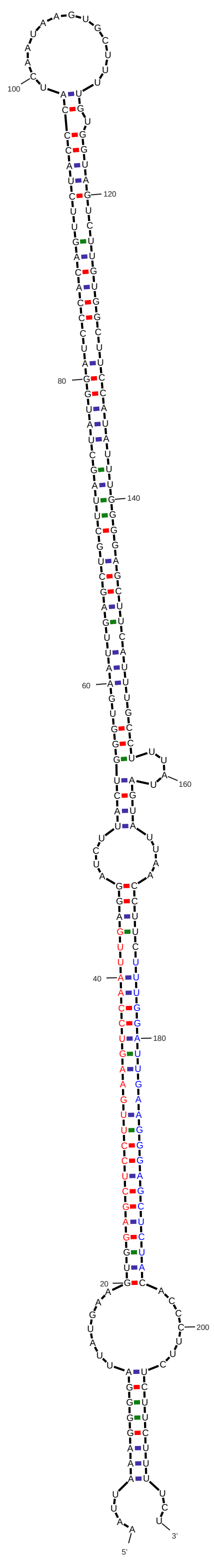

gma-miR159a

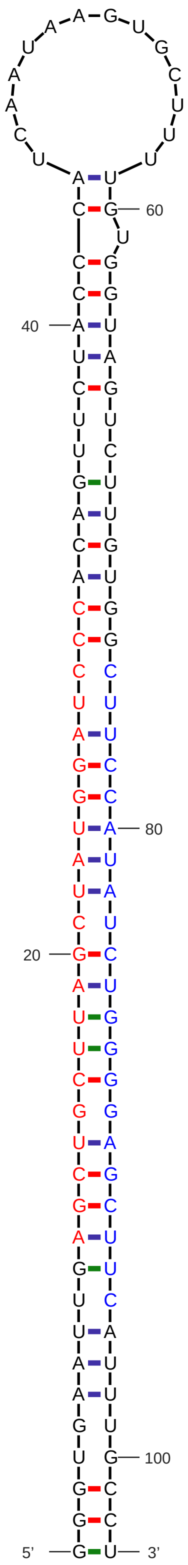

gma-miR159d

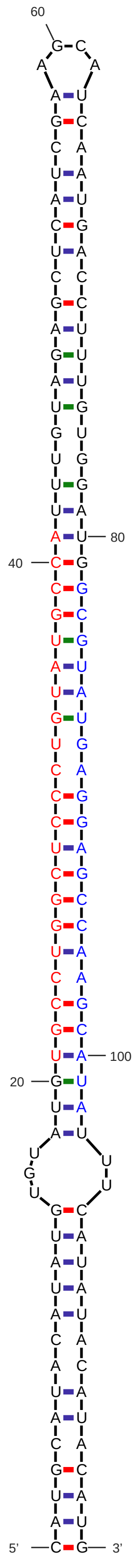

gma-miR160a

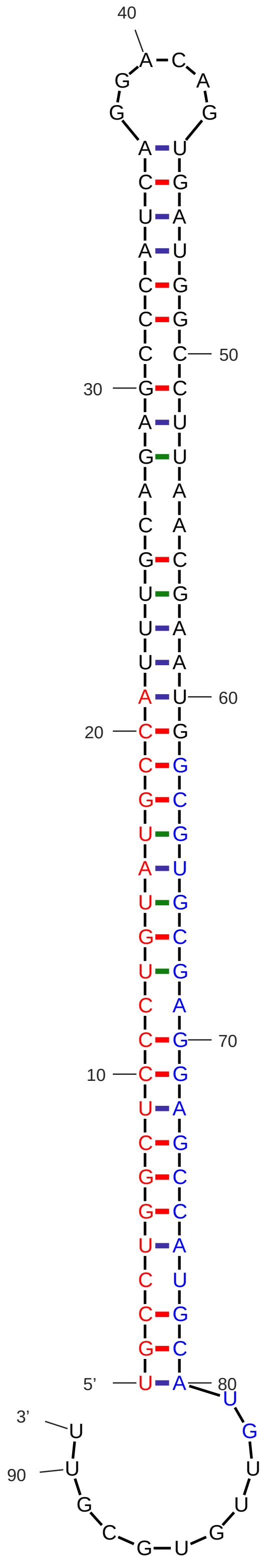

gma-miR160c

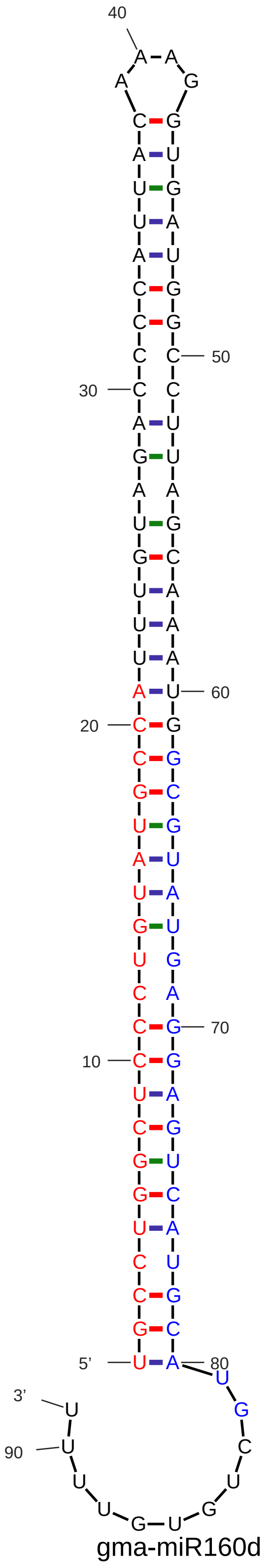

gma-miR160d

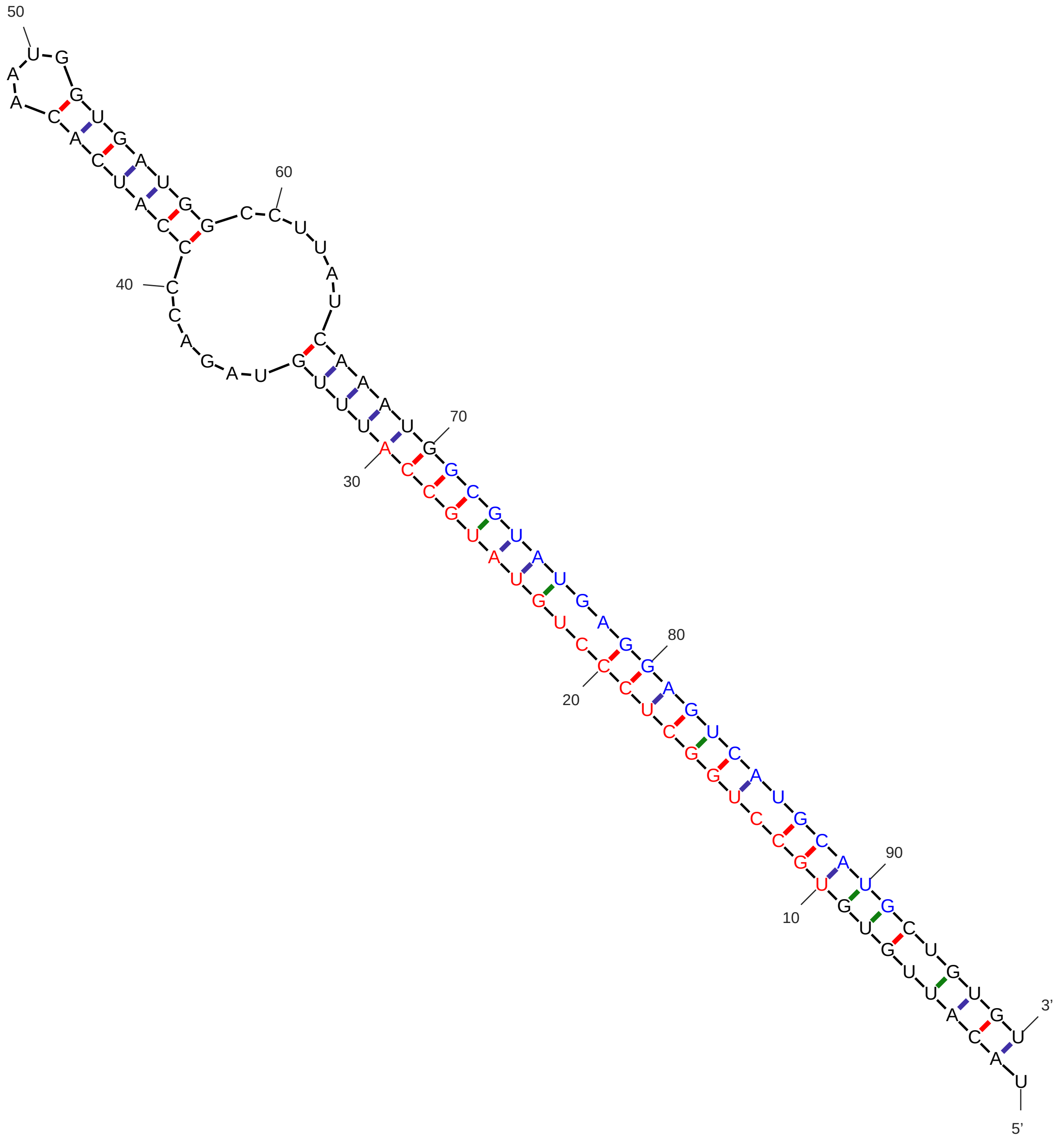

gma-miR160f

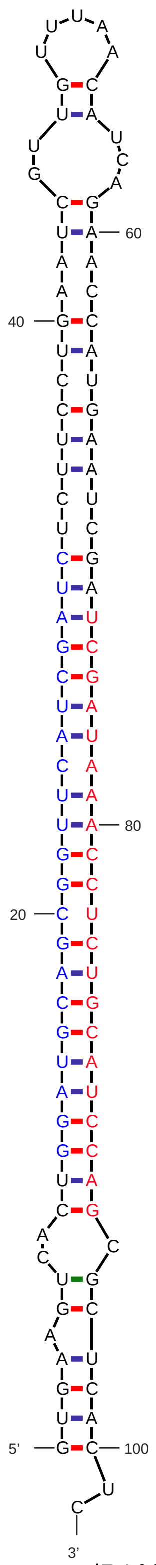

gma-miR162a

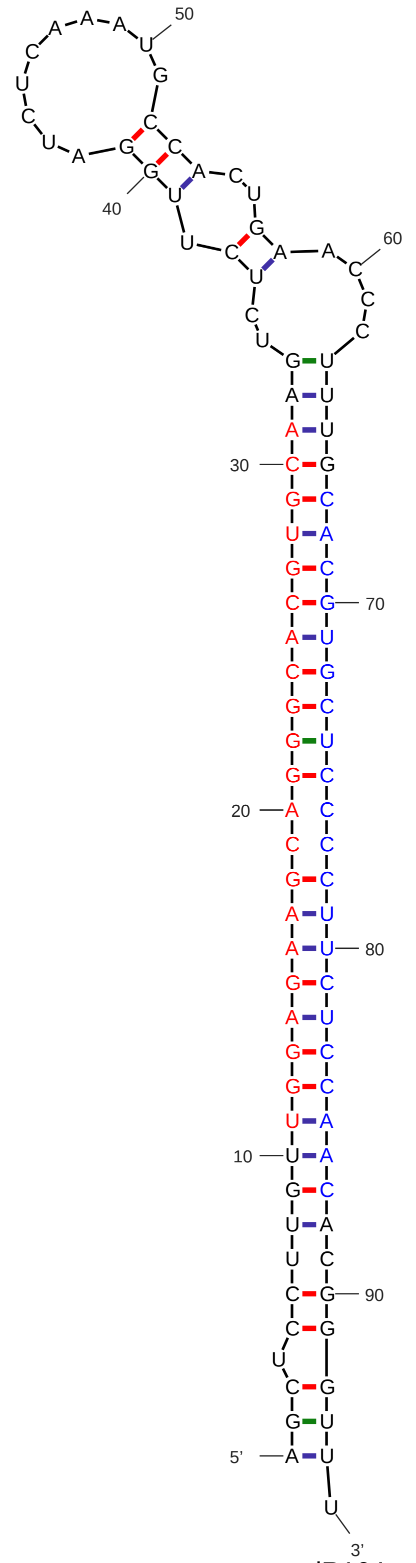

gma-miR164a

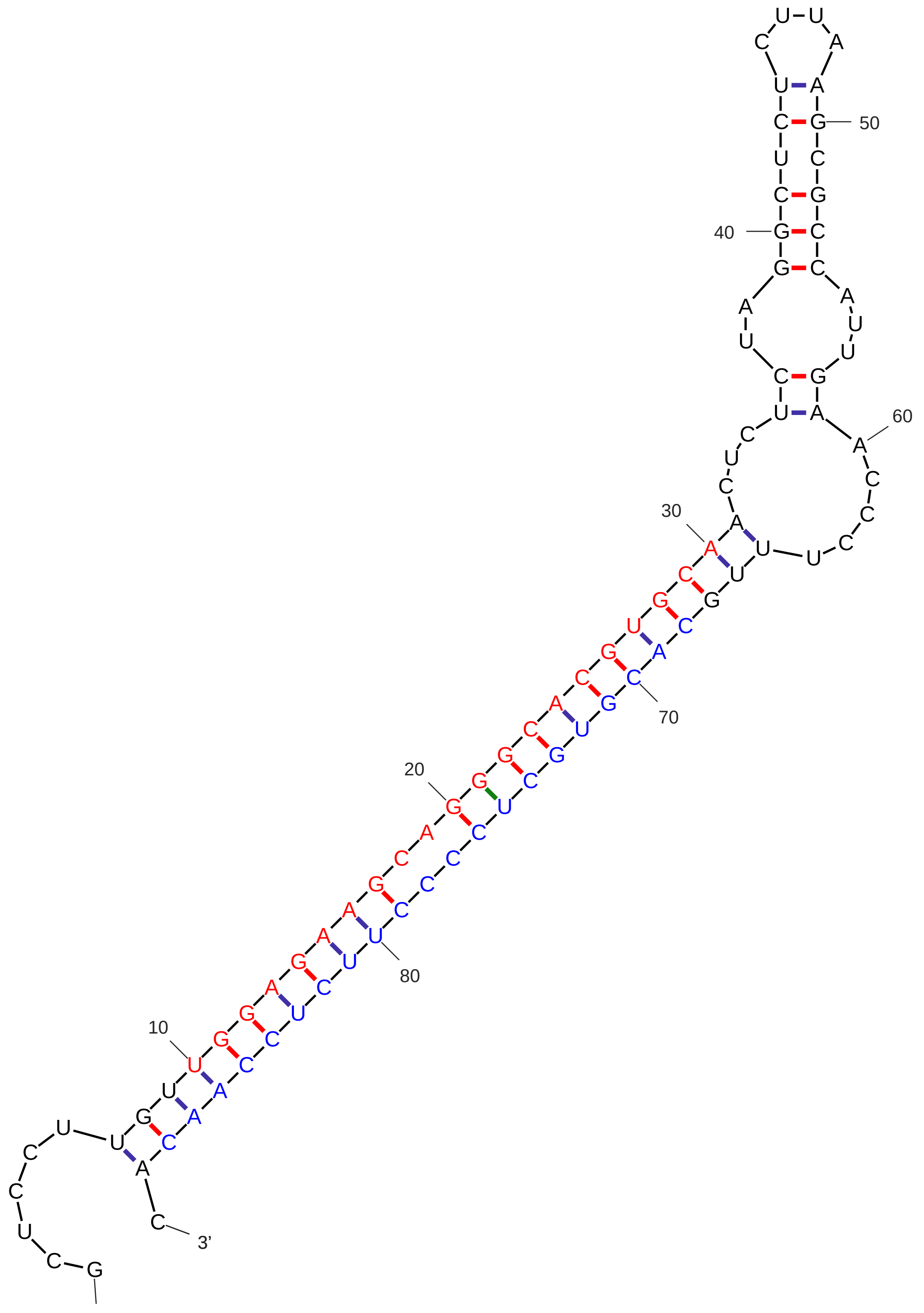

gma-miR164b

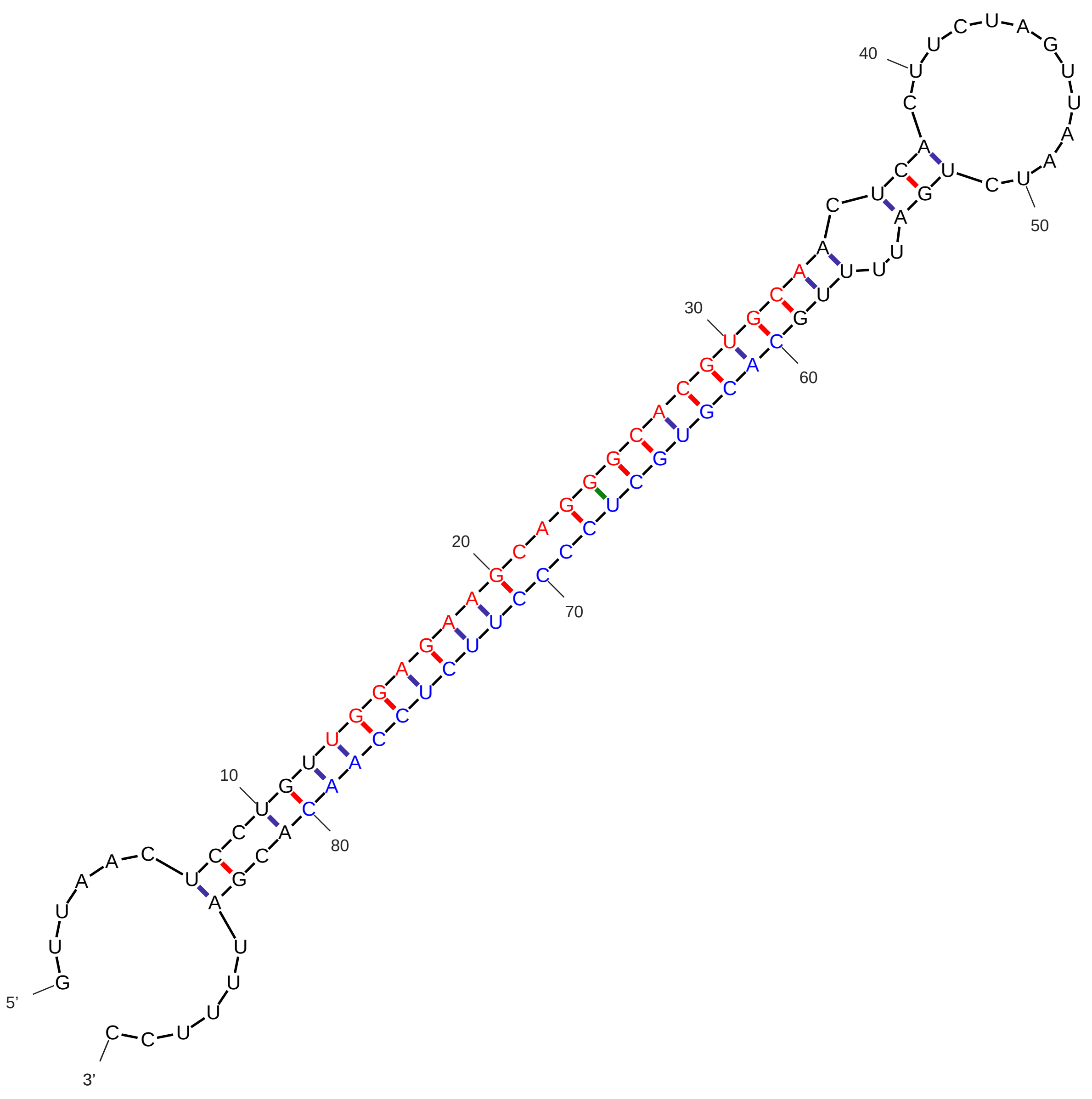

gma-miR164d

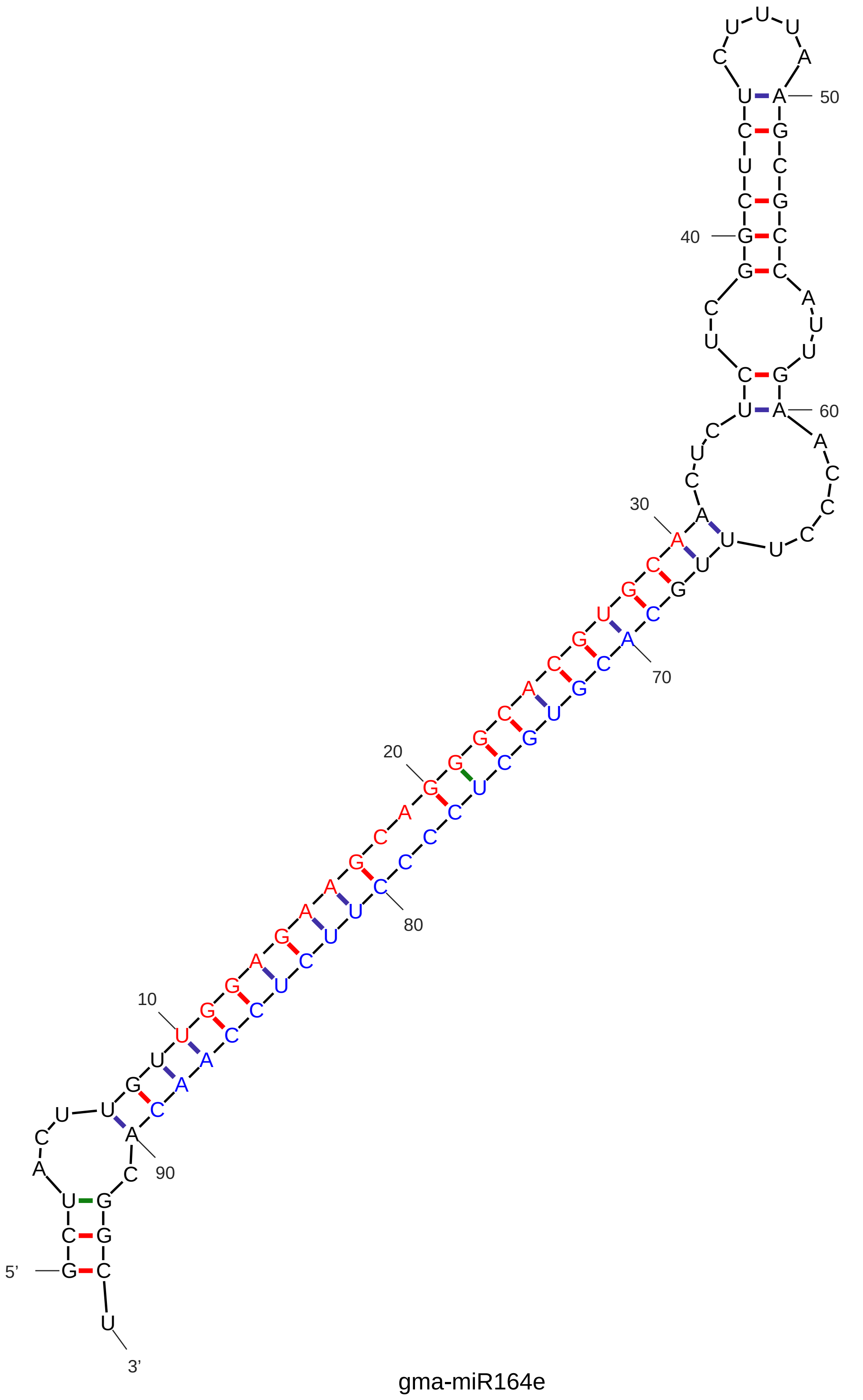

gma-miR164e

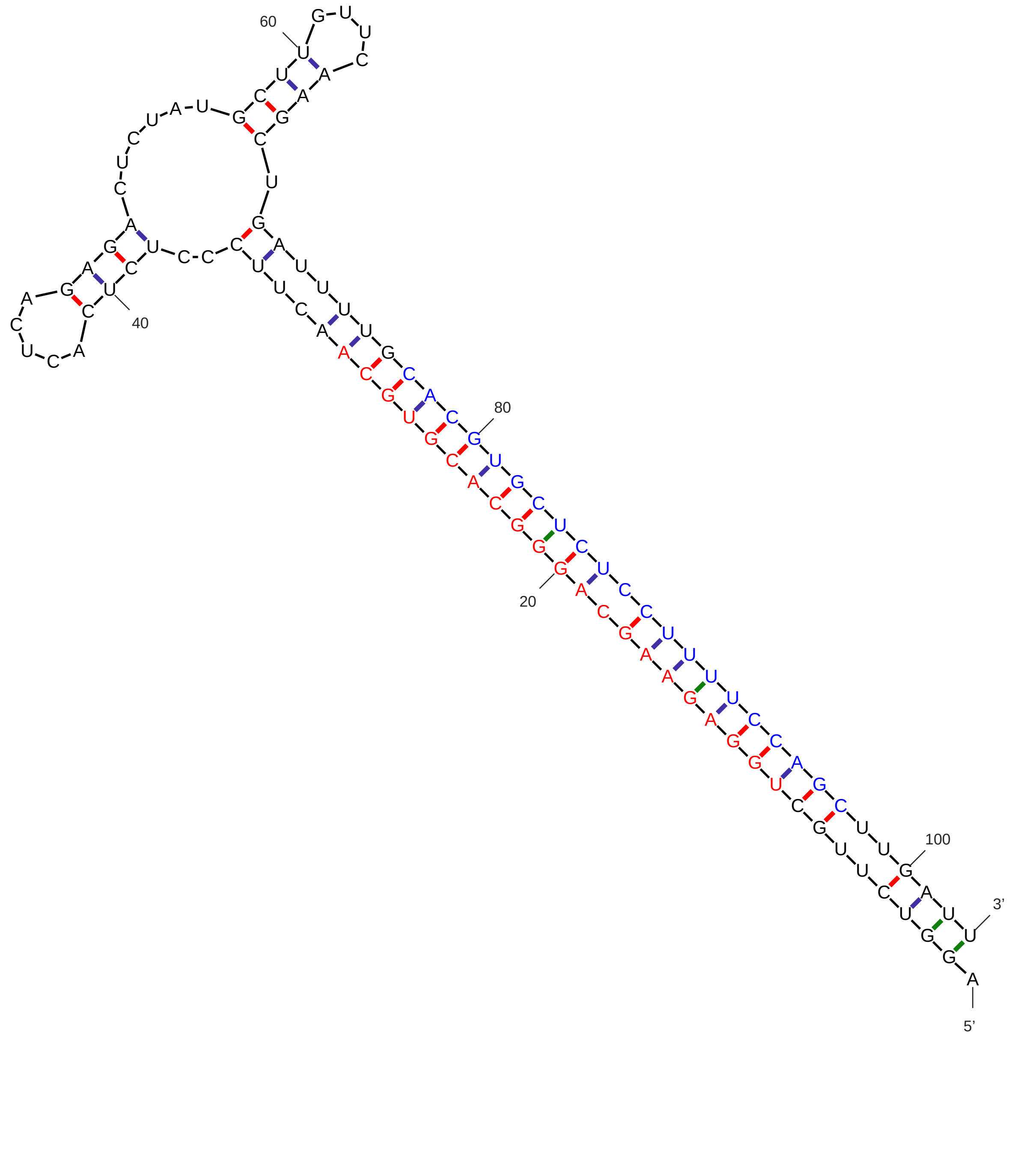

gma-miR164f

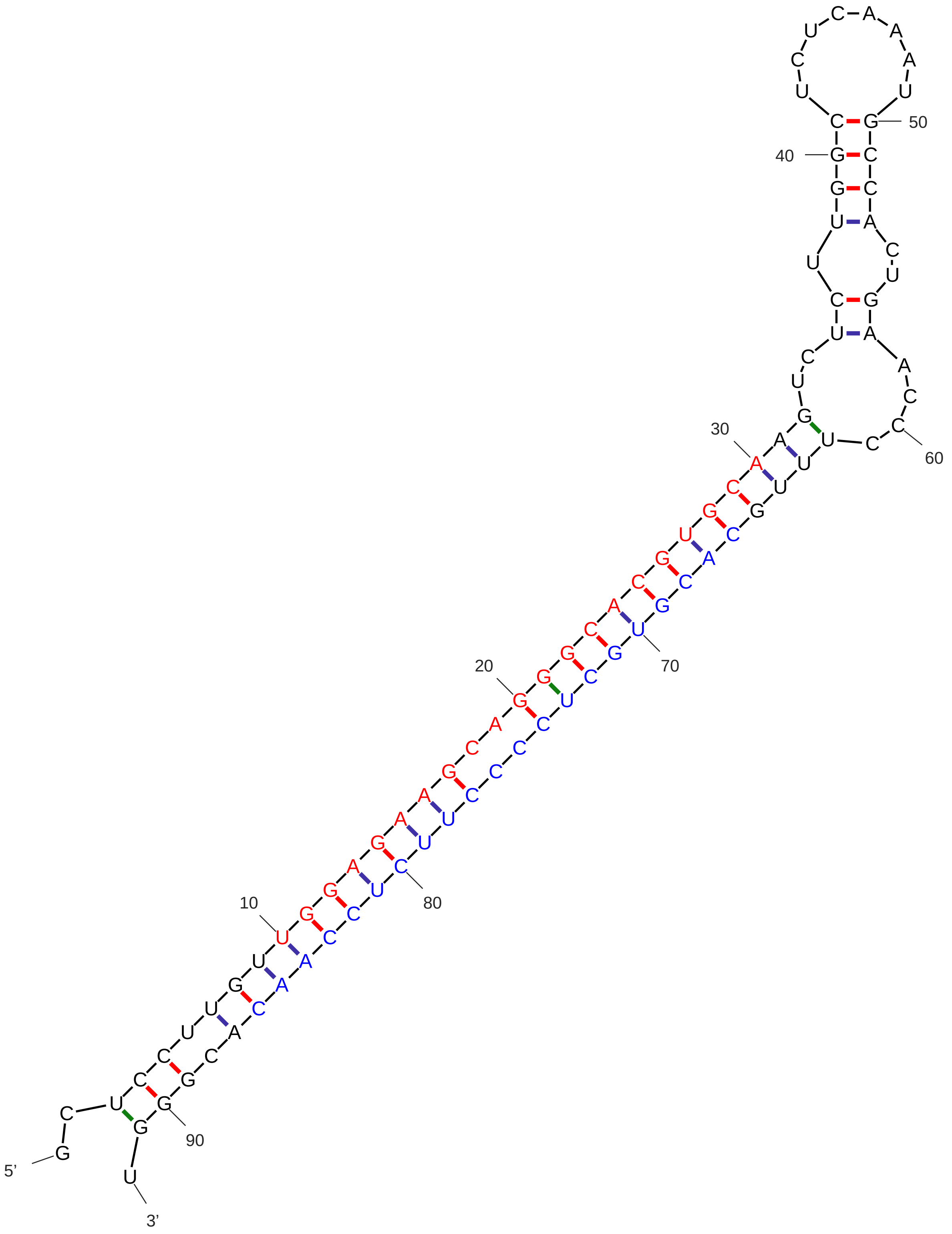

gma-miR164g

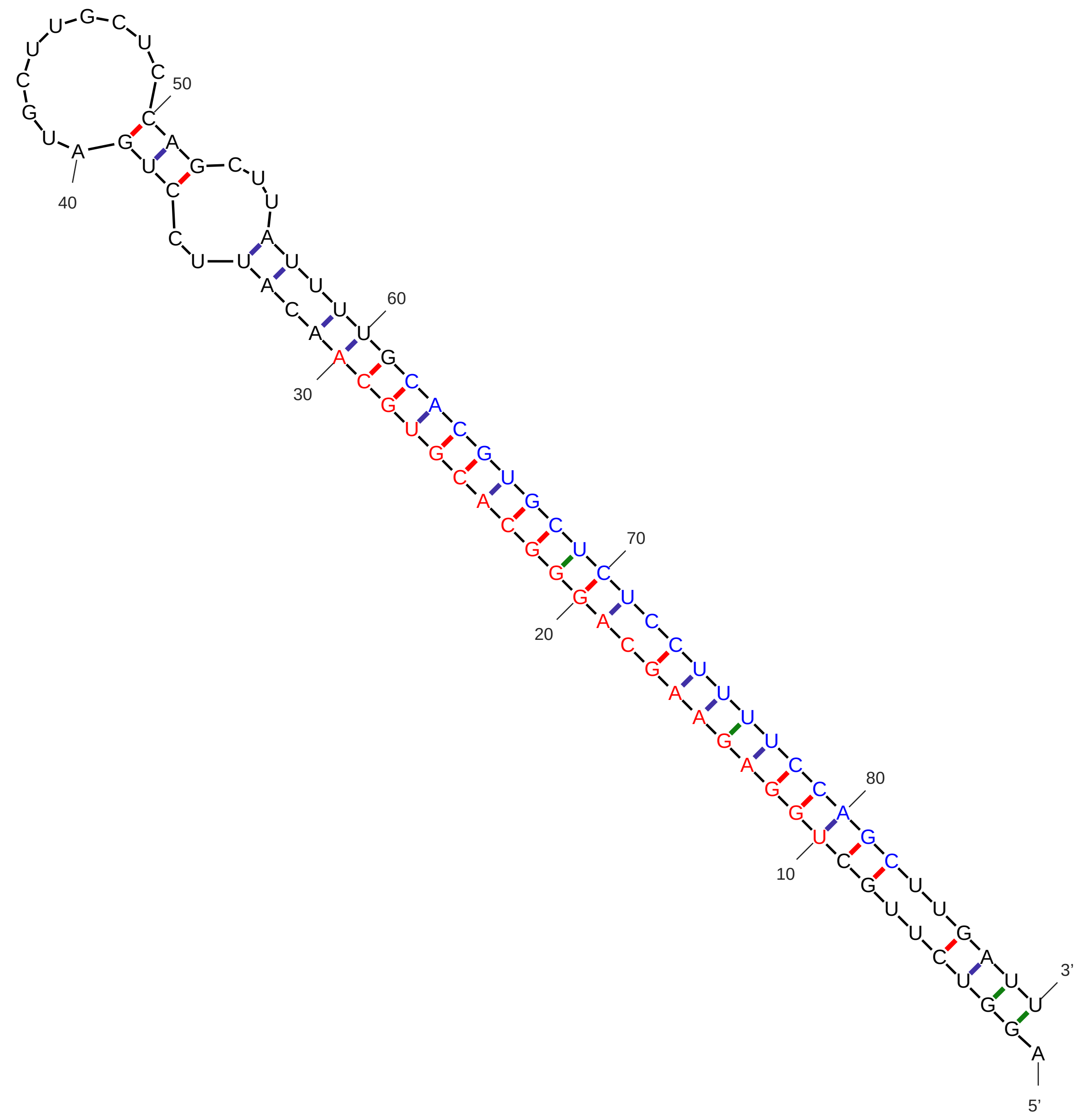

gma-miR164j

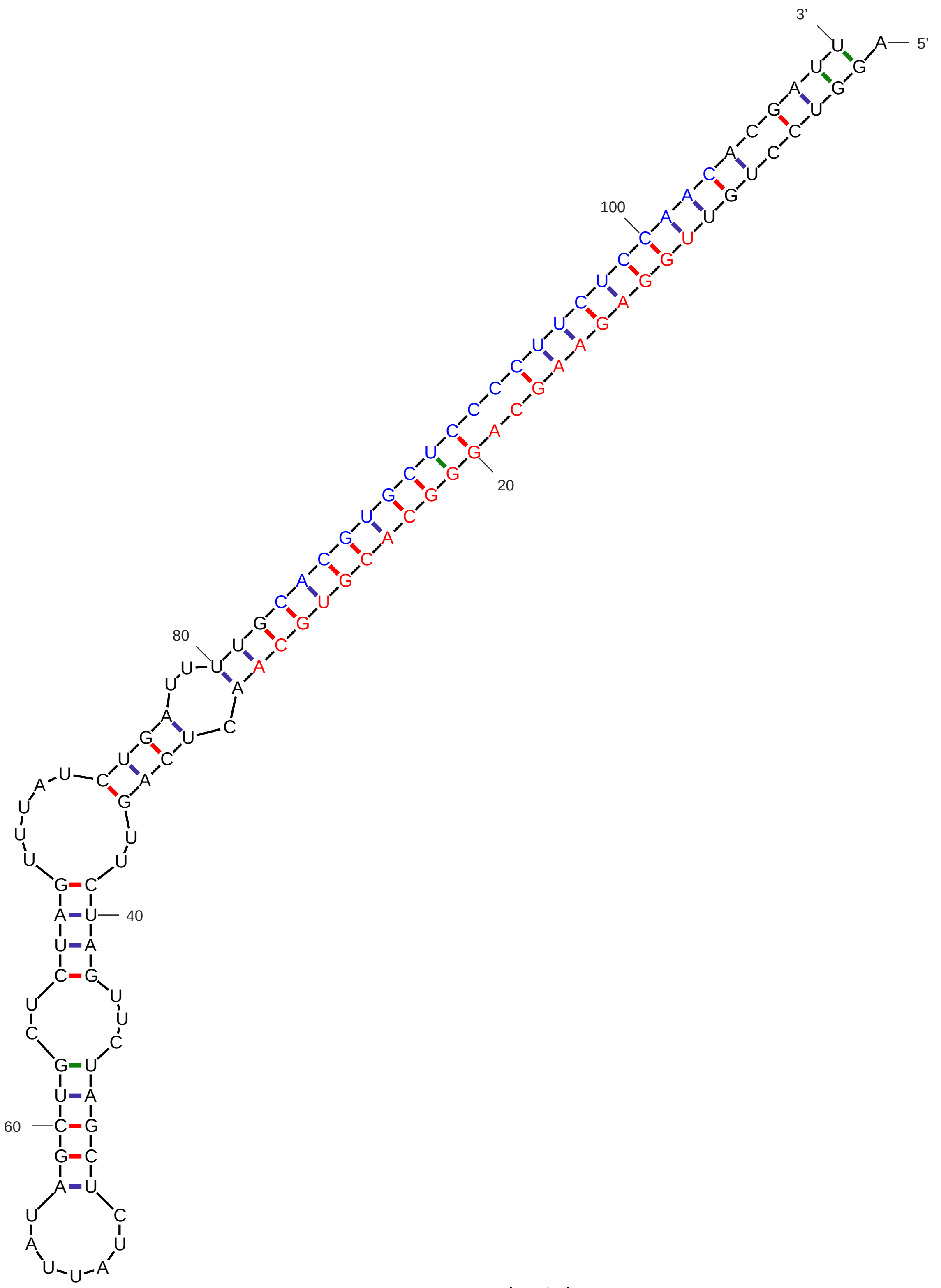

gma-miR164k

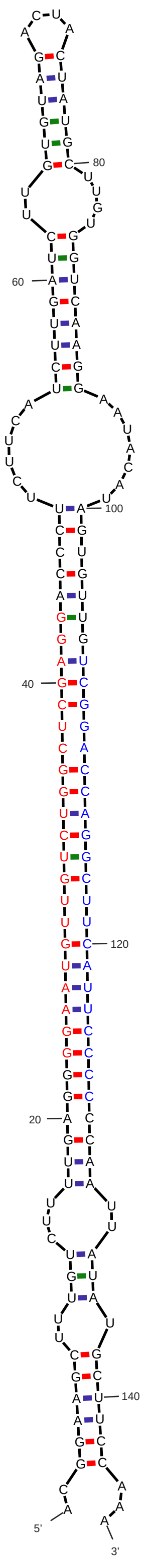

gma-miR166a

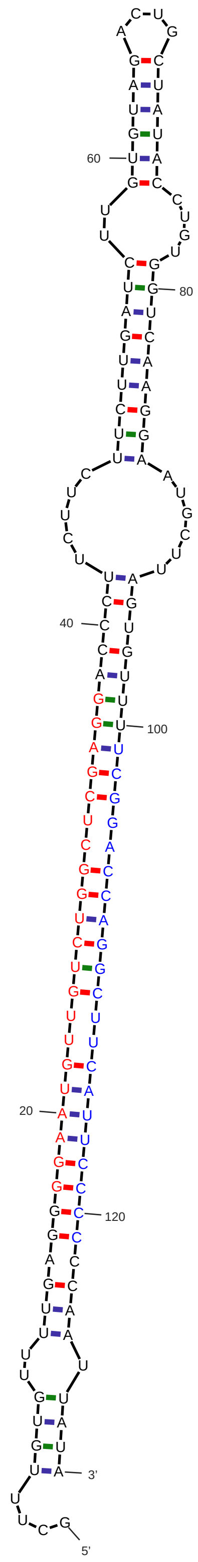

gma-miR166c

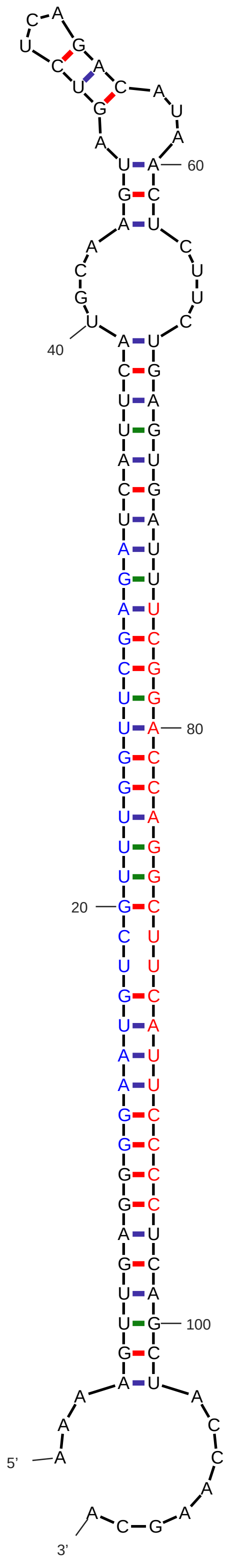

gma-miR166g

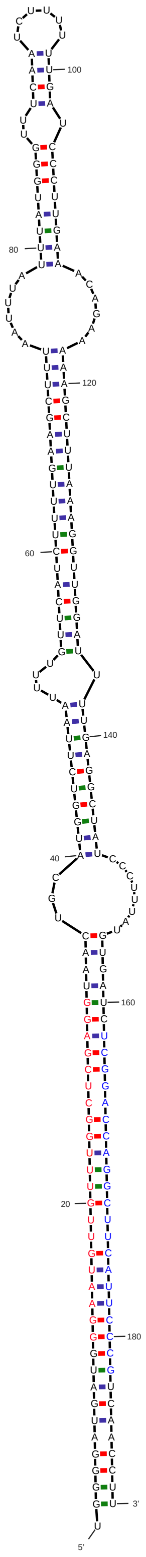

gma-miR166h

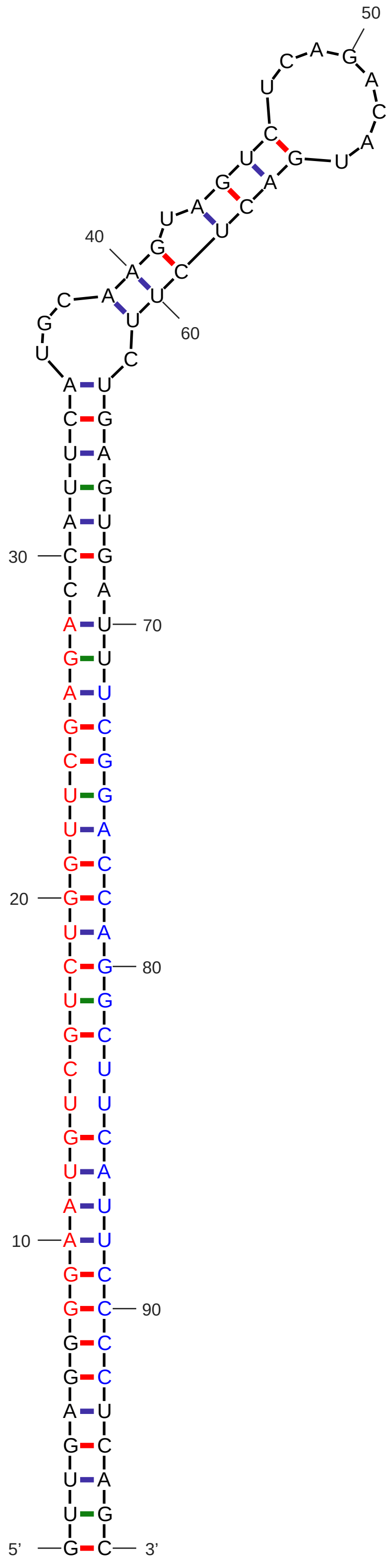

gma-miR166i

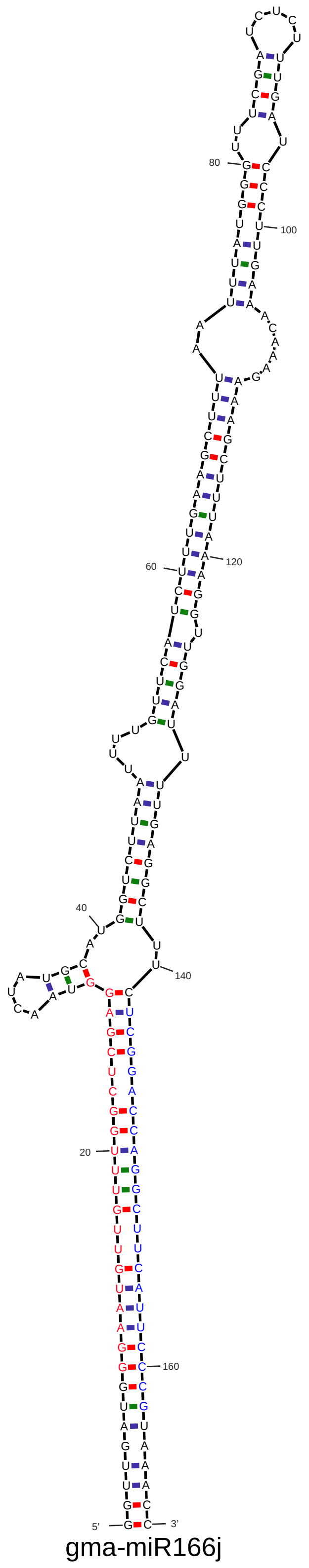

gma-miR166j

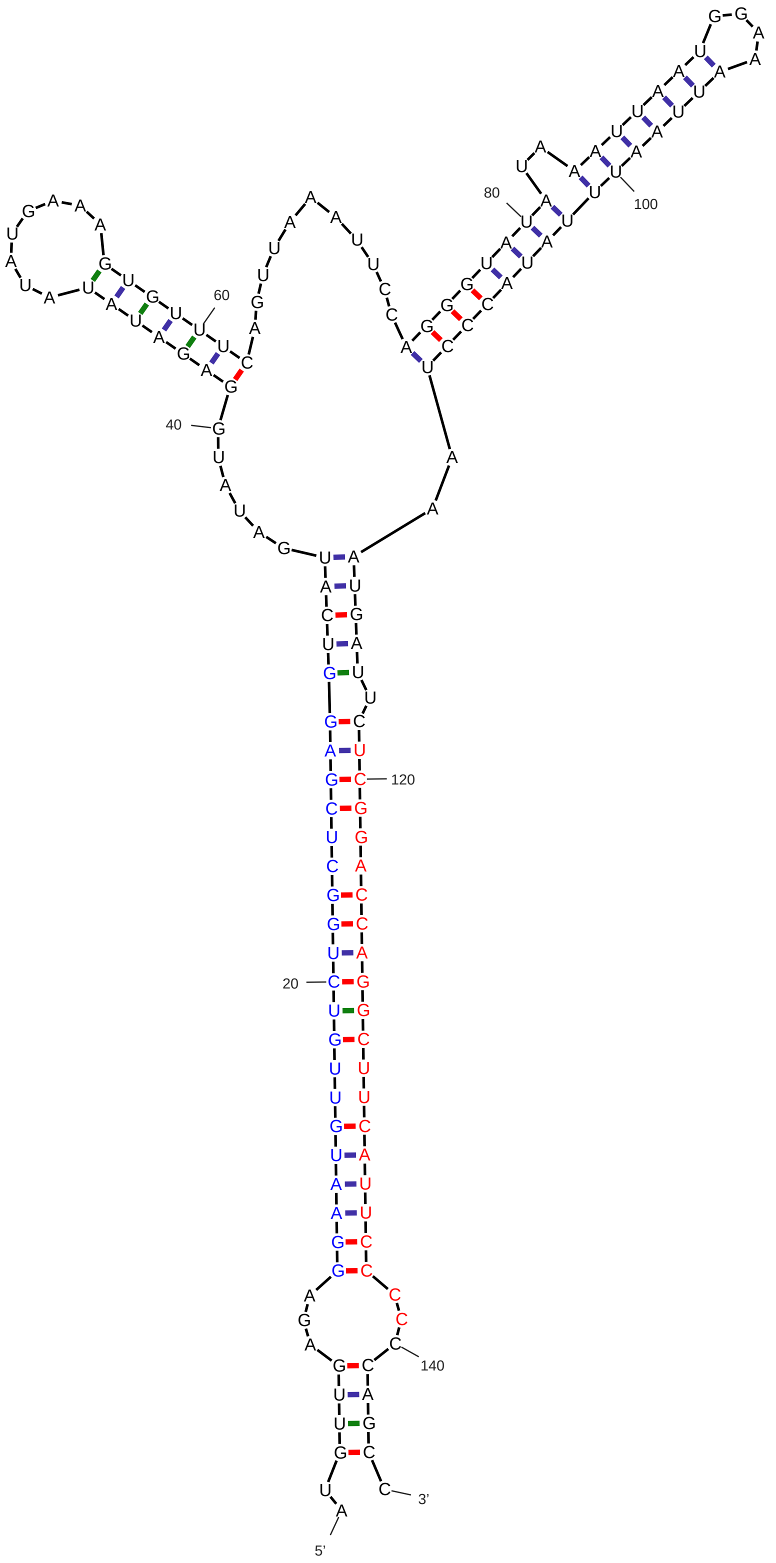

gma-miR166o

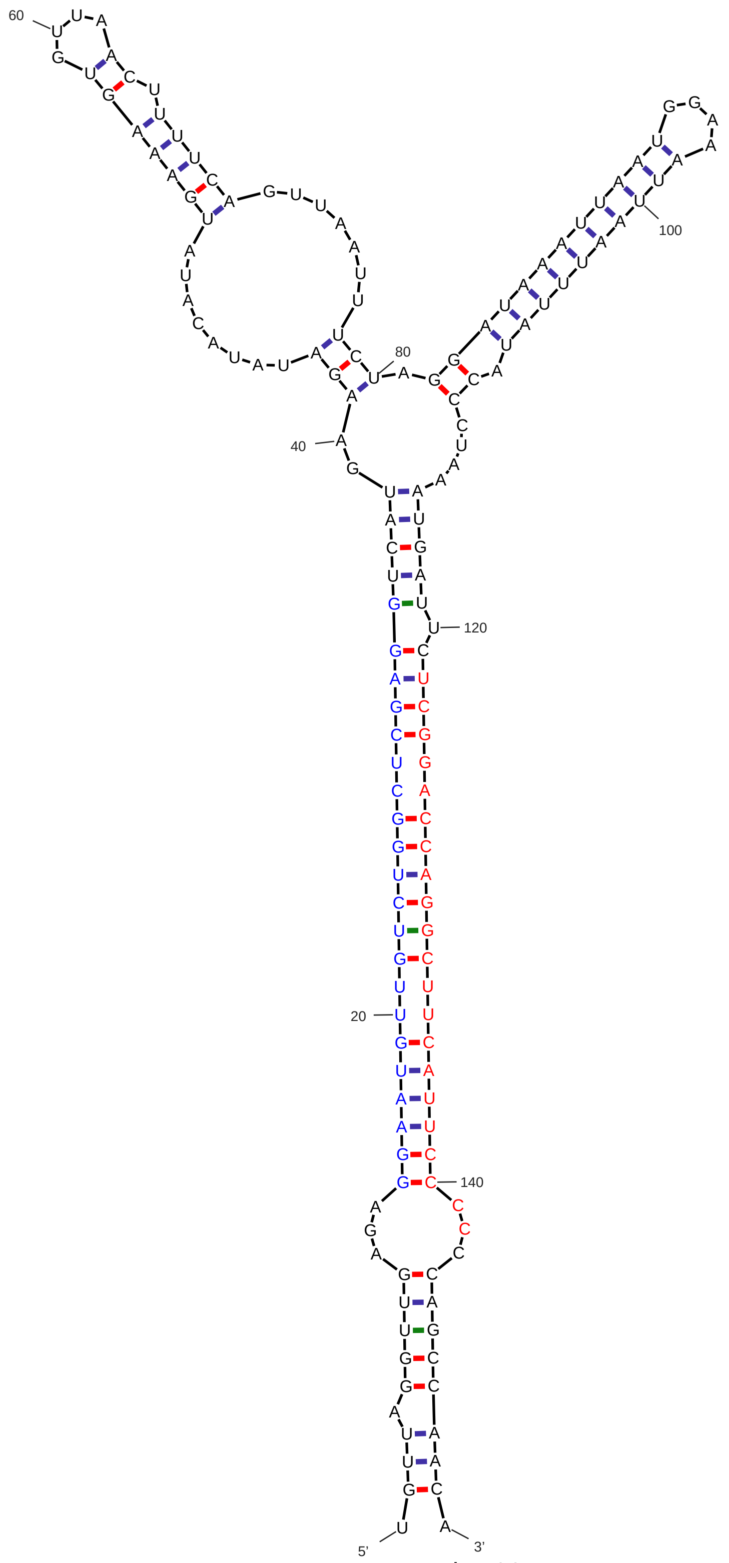

gma-miR166q

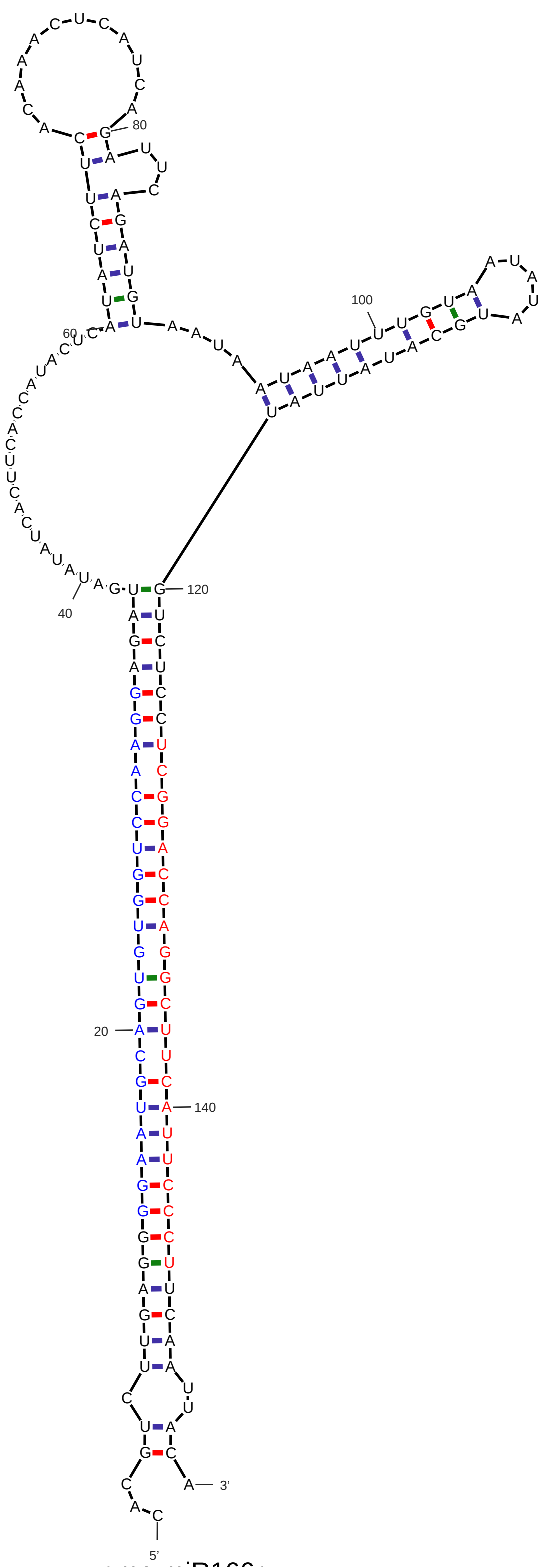

gma-miR166r

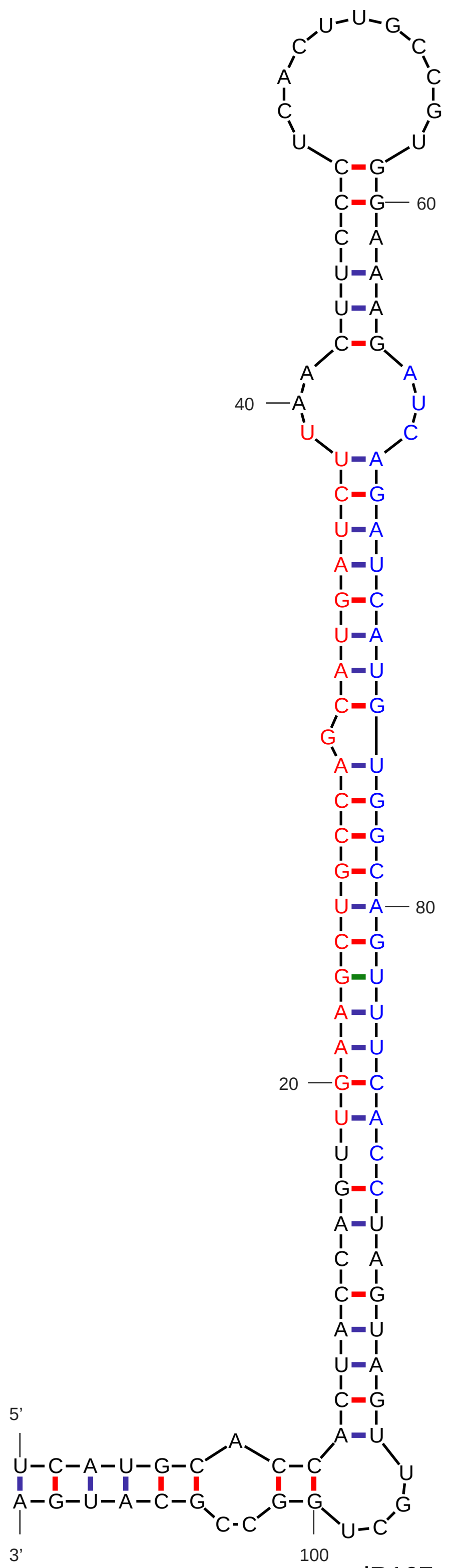

gma-miR167e

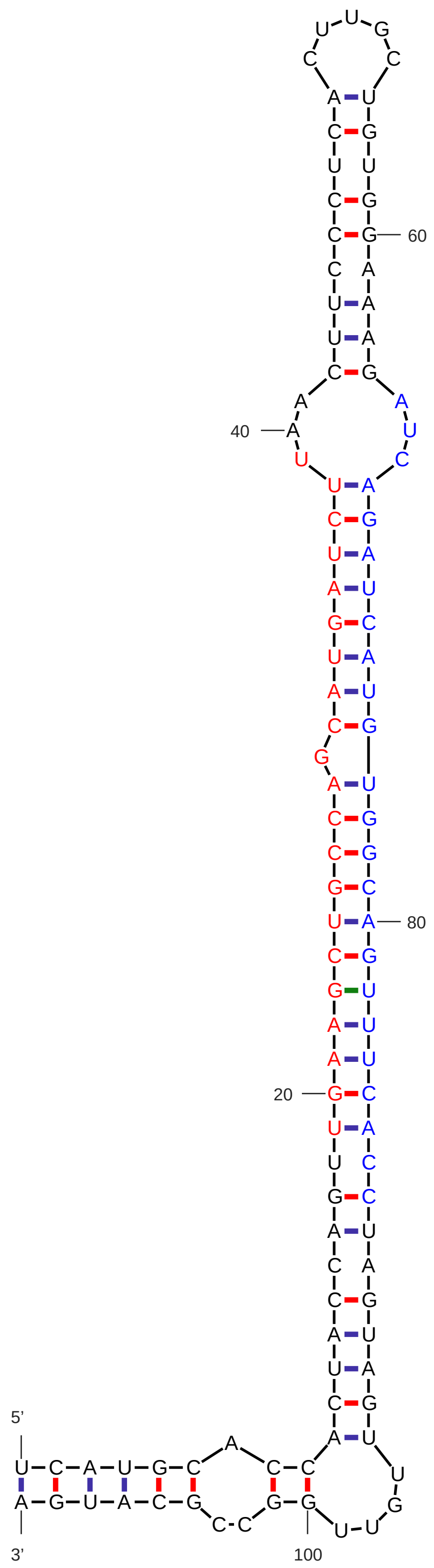

gma-miR167f

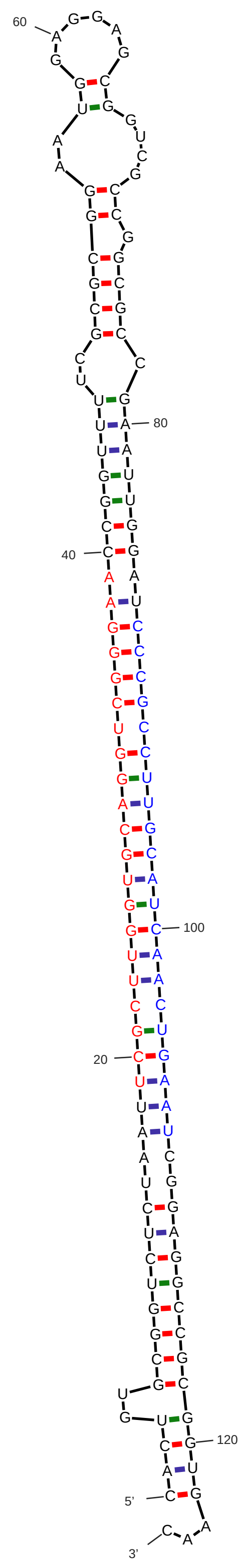

gma-miR168a

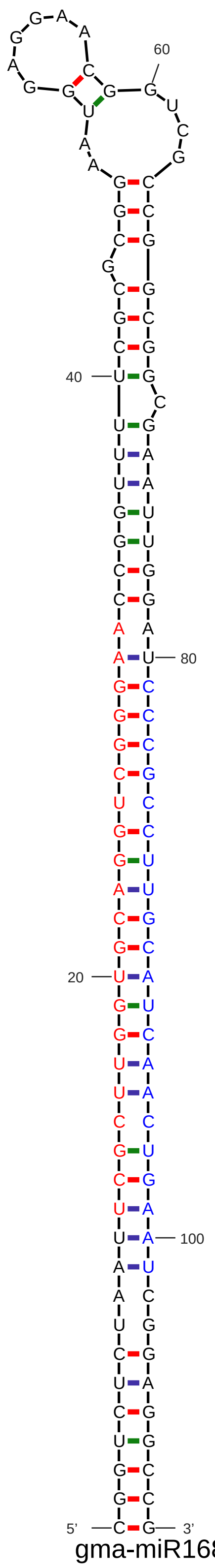

gma-miR168b

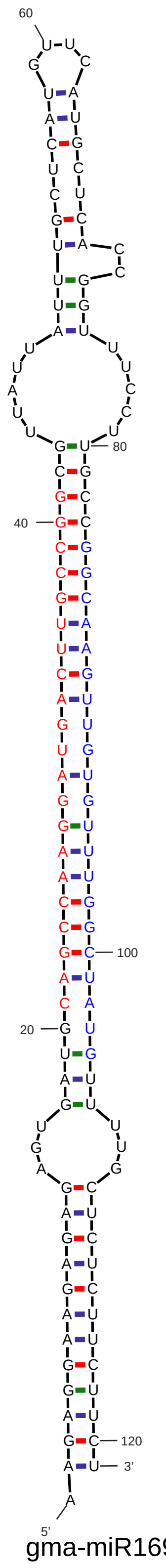

gma-miR169a

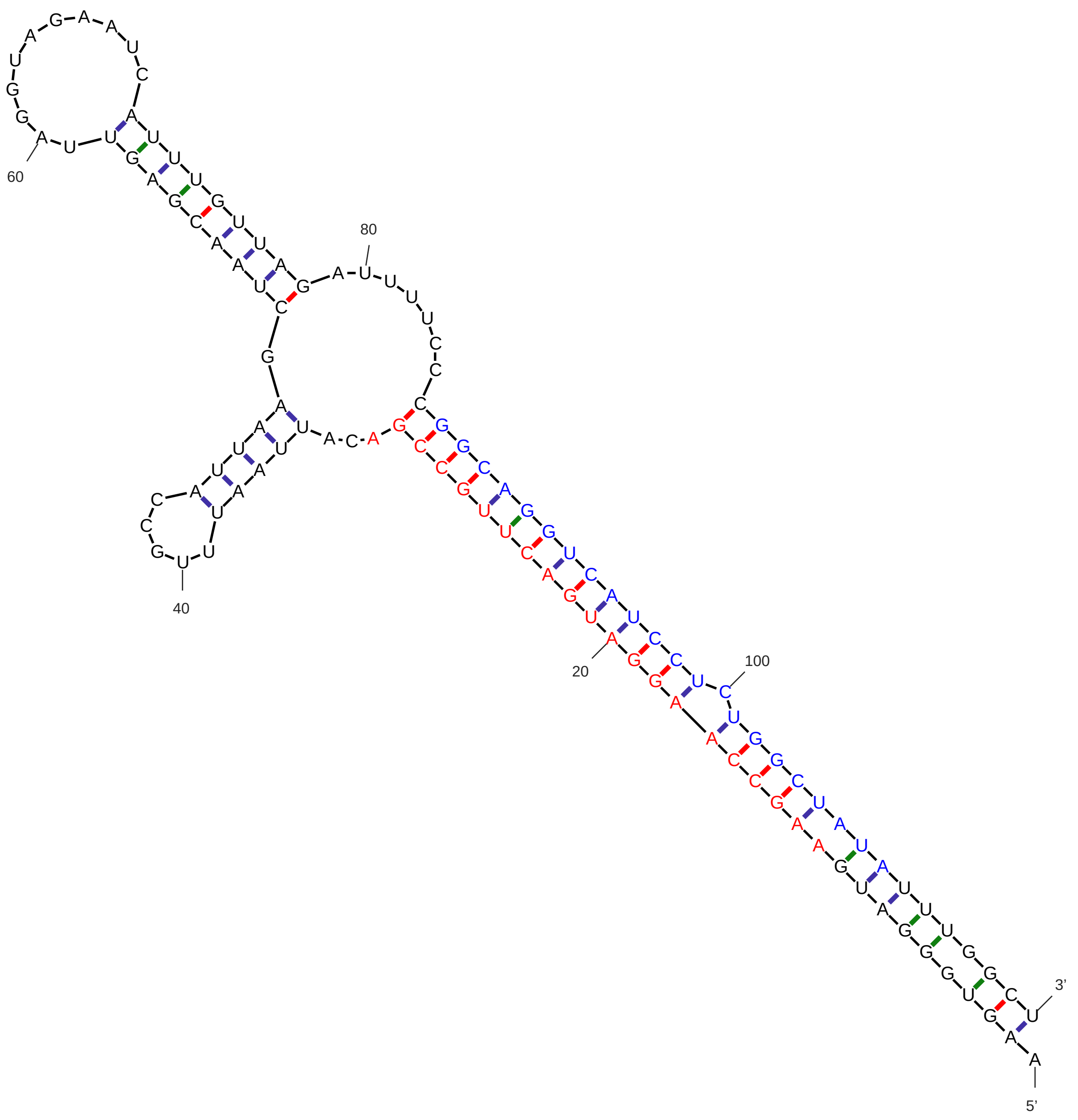

gma-miR169c

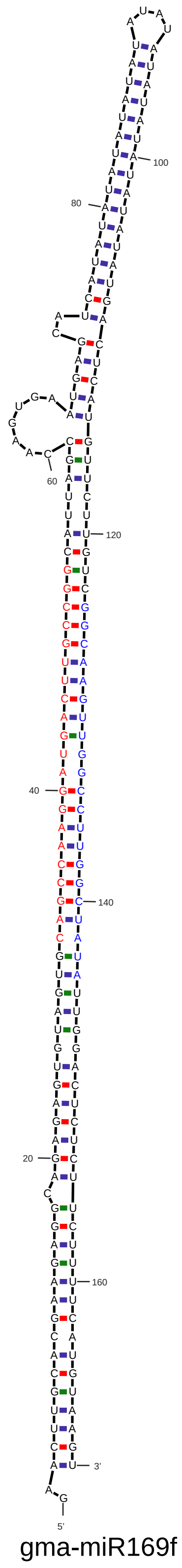

gma-miR169f

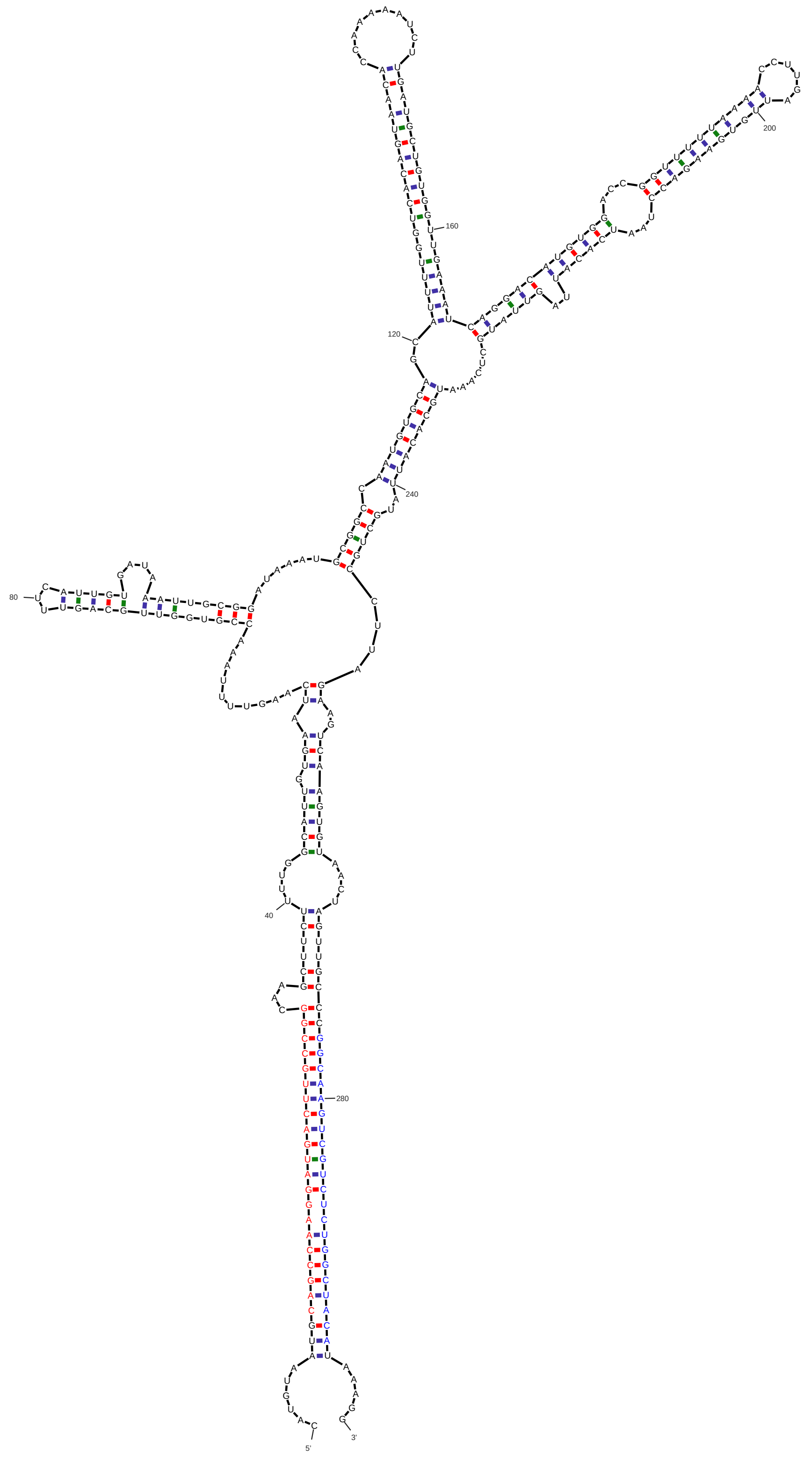

gma-miR169v

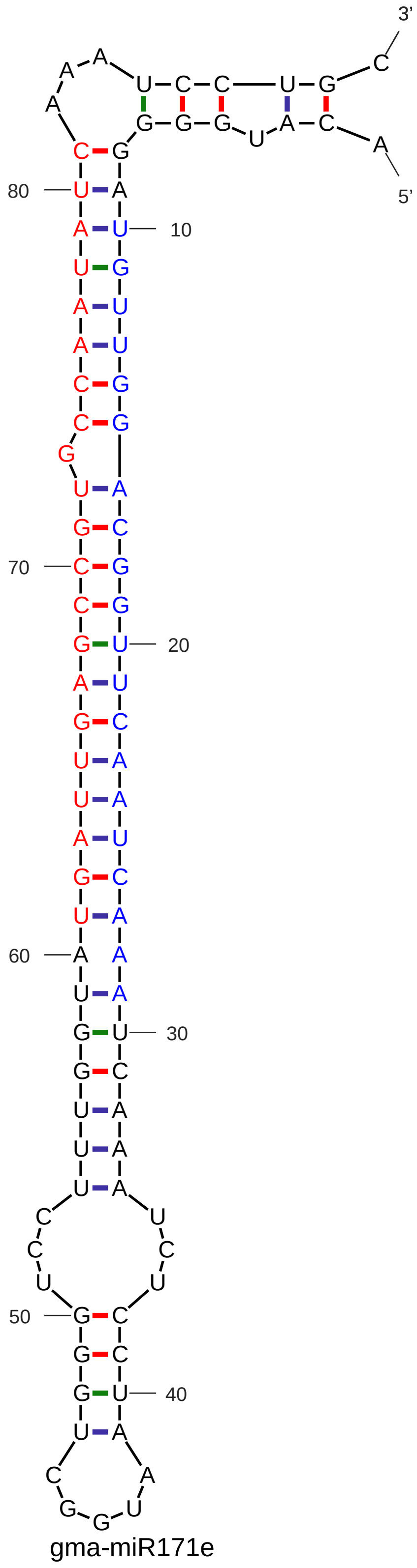

gma-miR171e

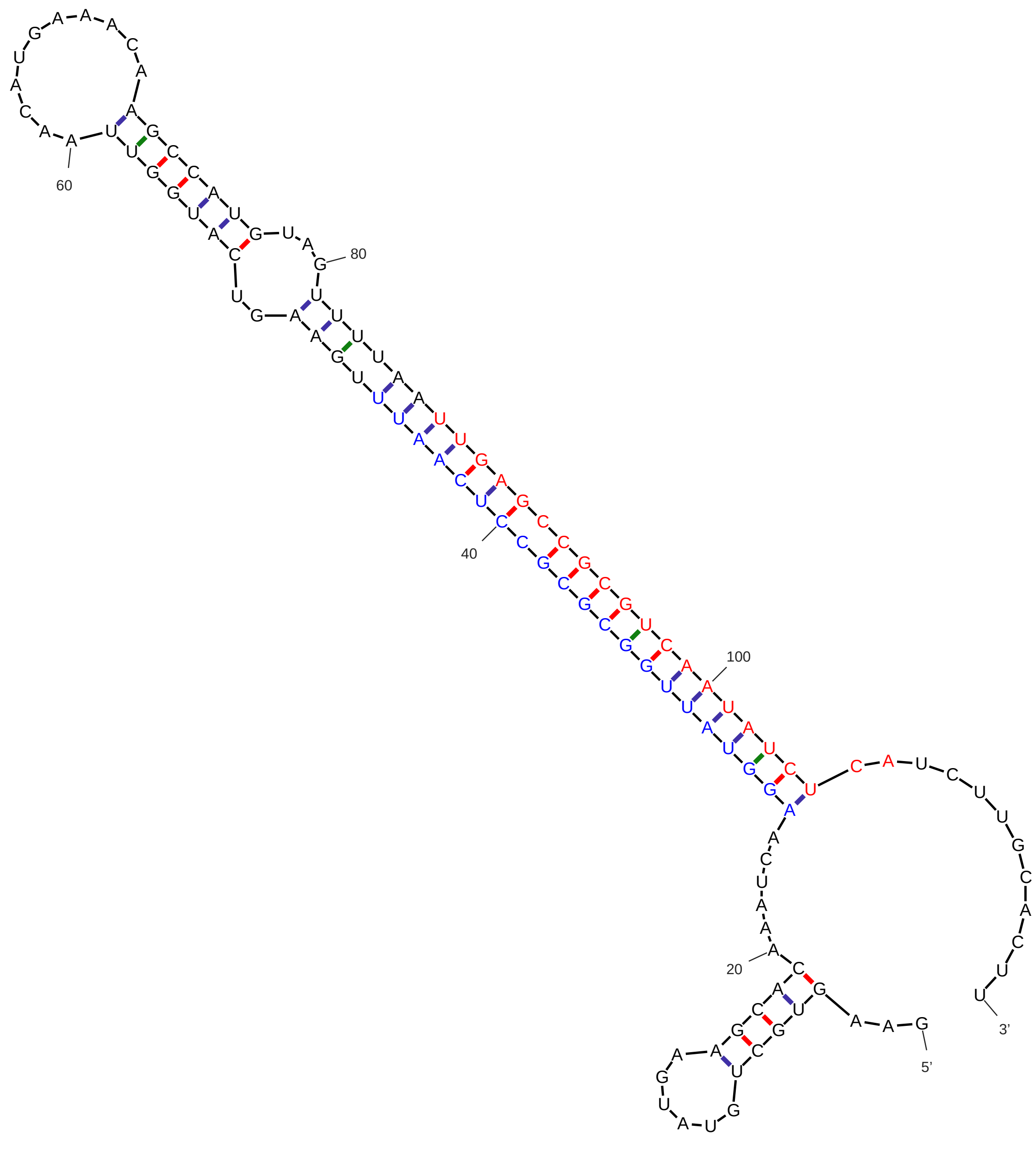

gma-miR171t

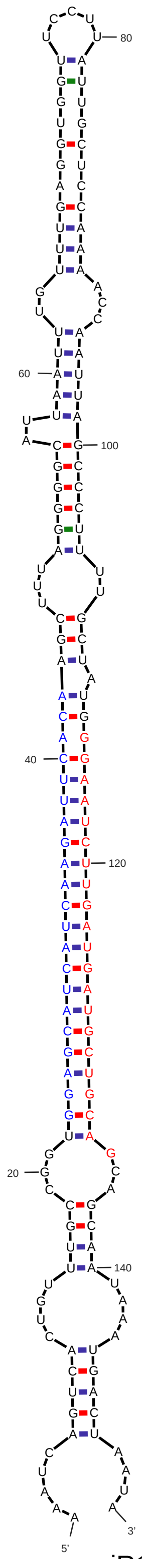

gma-miR172c

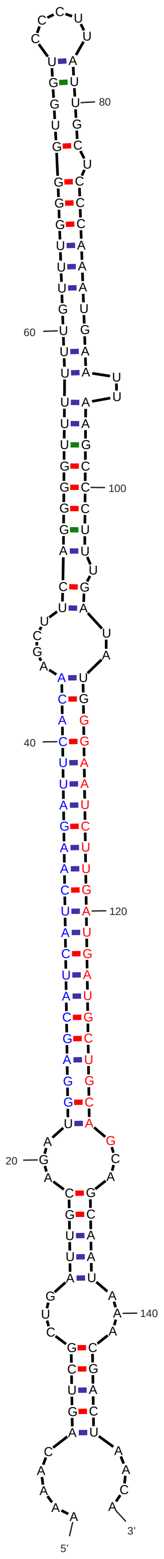

gma-miR172d

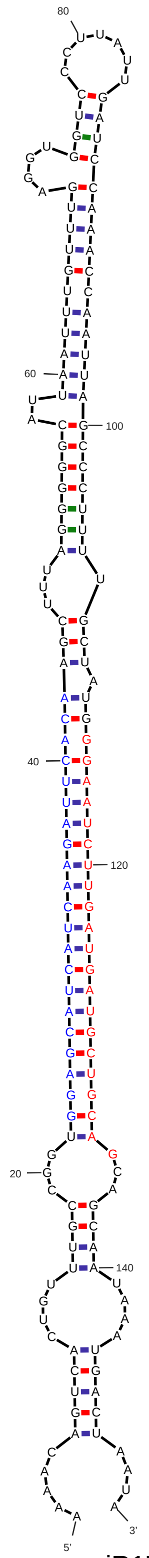

gma-miR172e

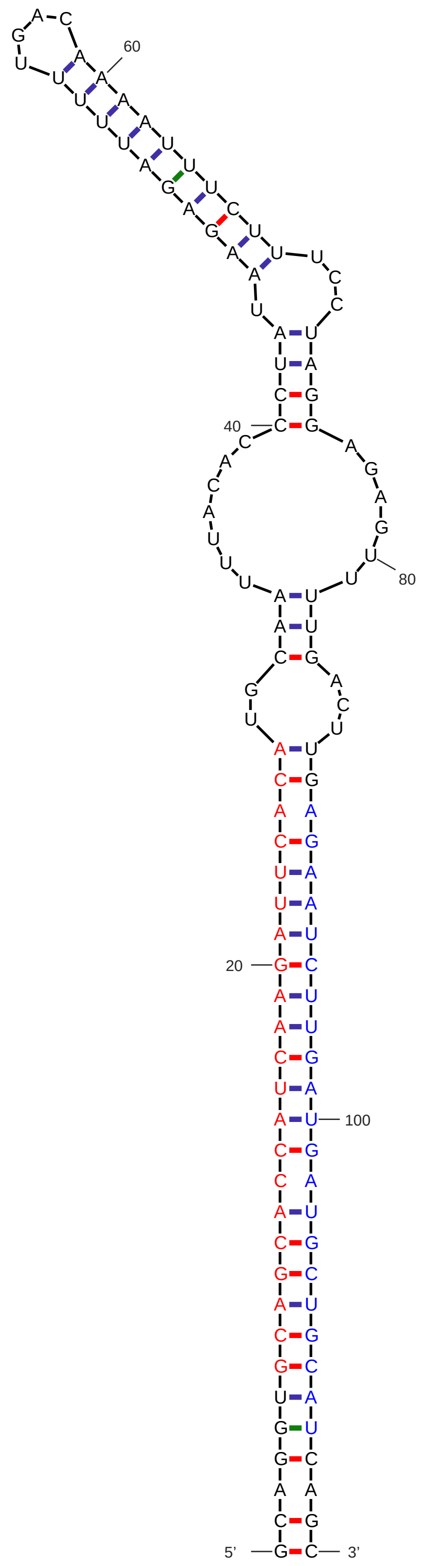

gma-miR172g

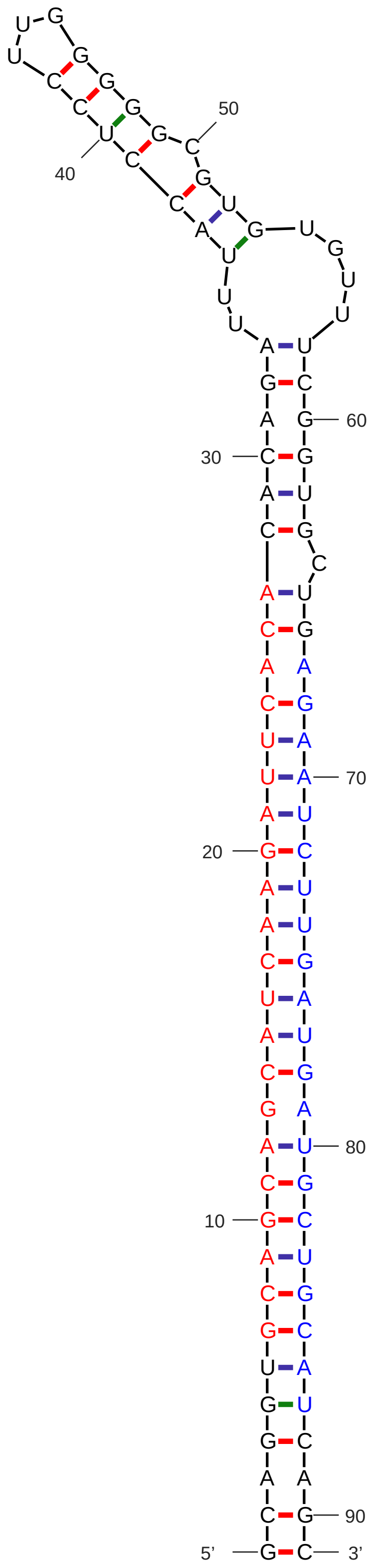

gma-miR172h

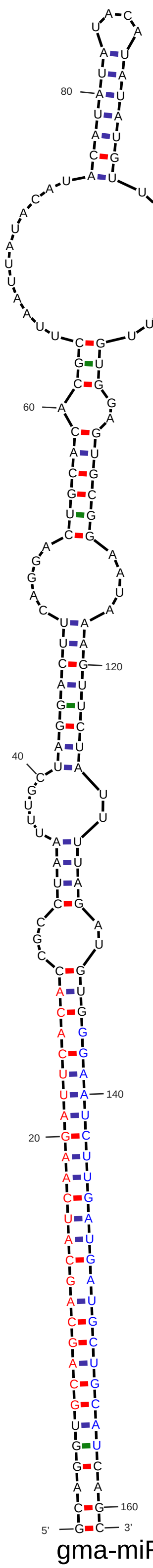

gma-miR172i

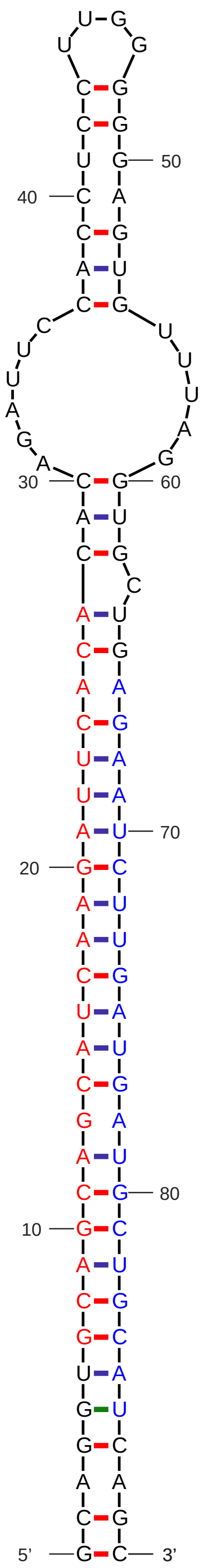
gma-miR172j

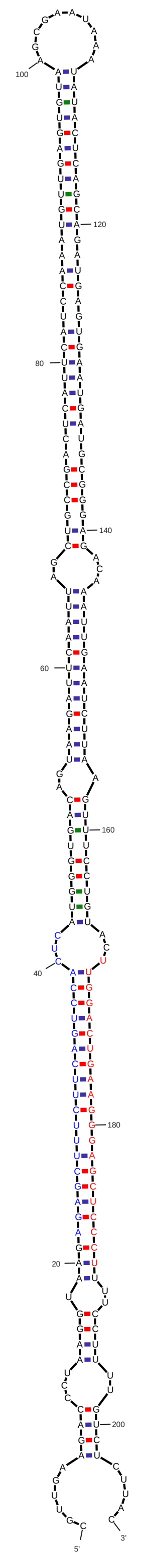
gma-miR319a

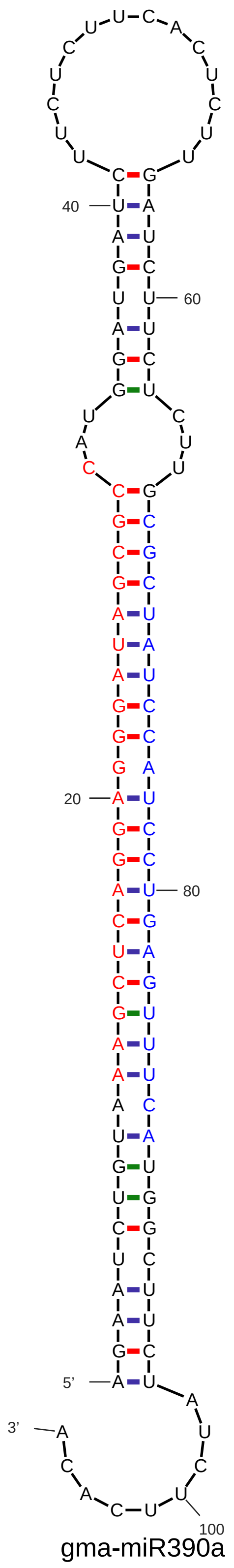
gma-miR390a

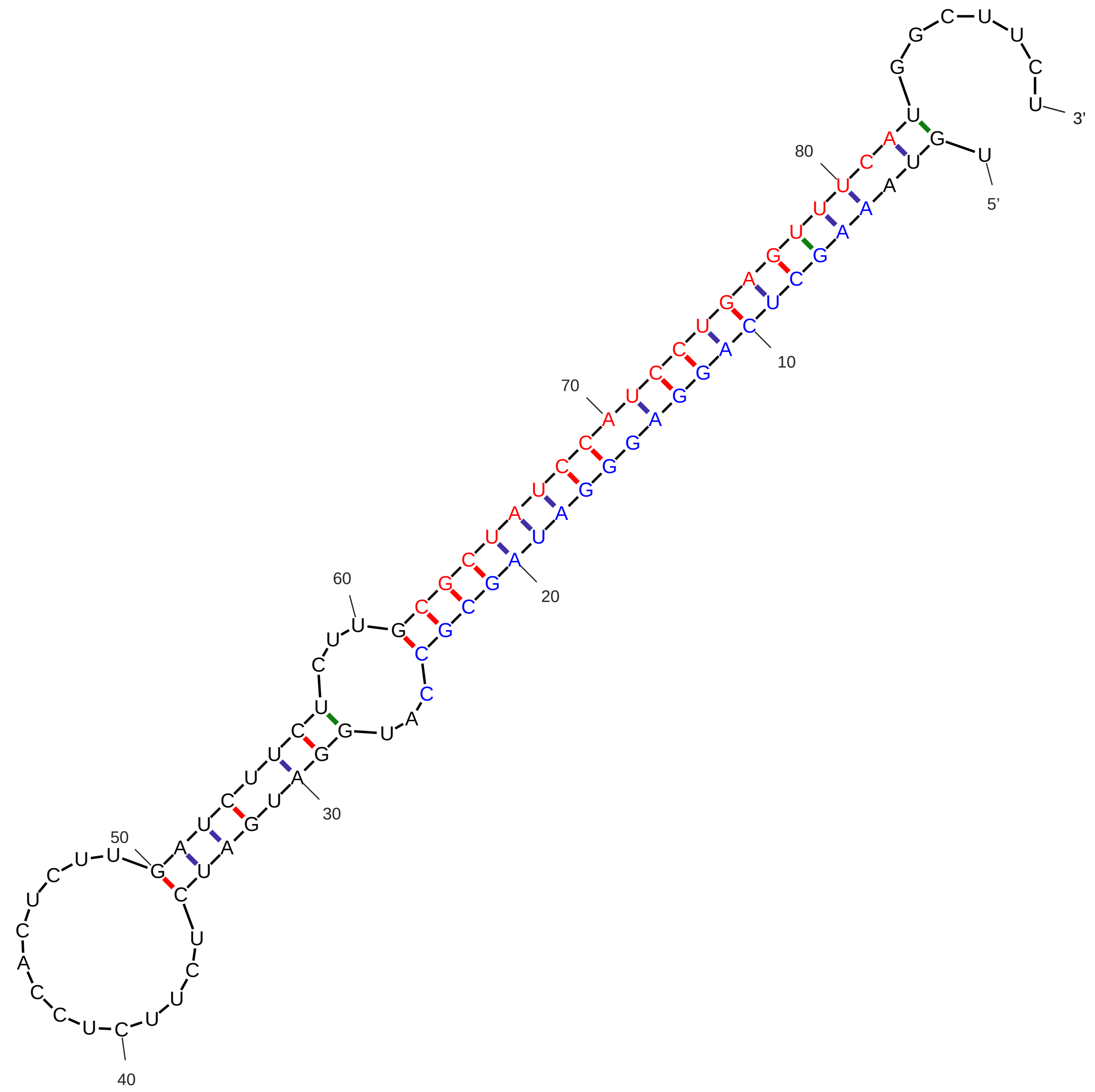
gma-miR390c

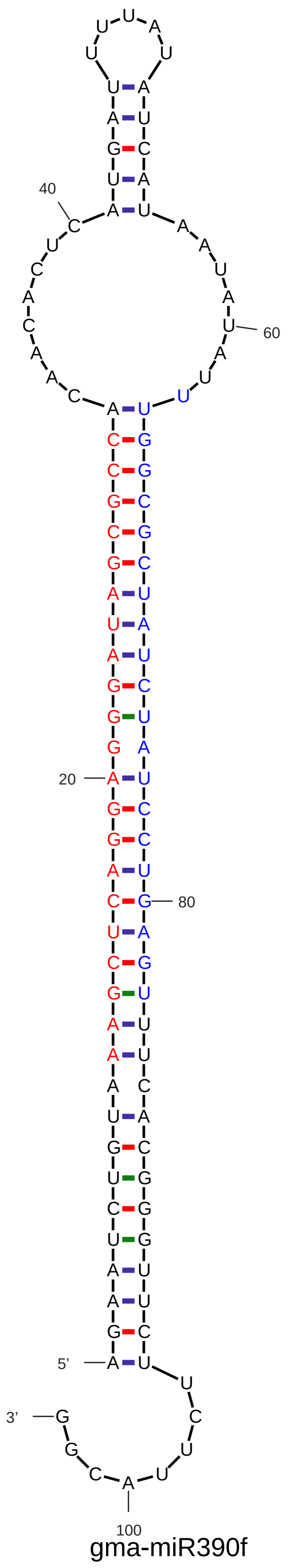

gma-miR390f

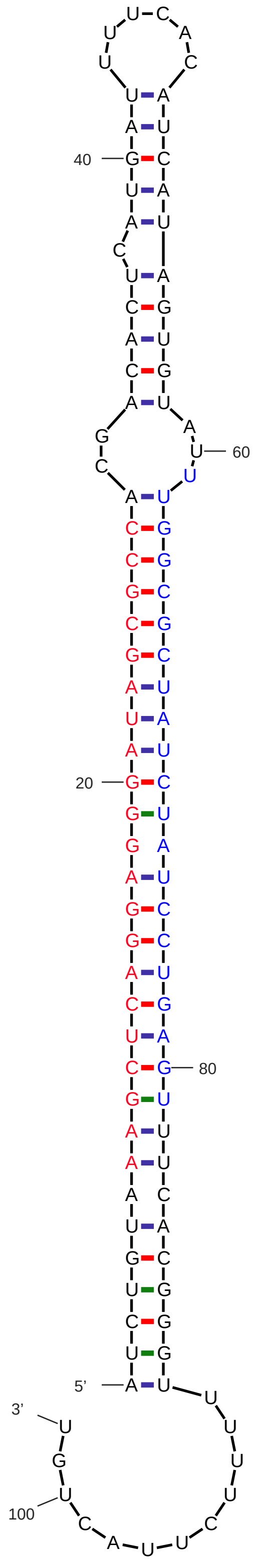

gma-miR390g

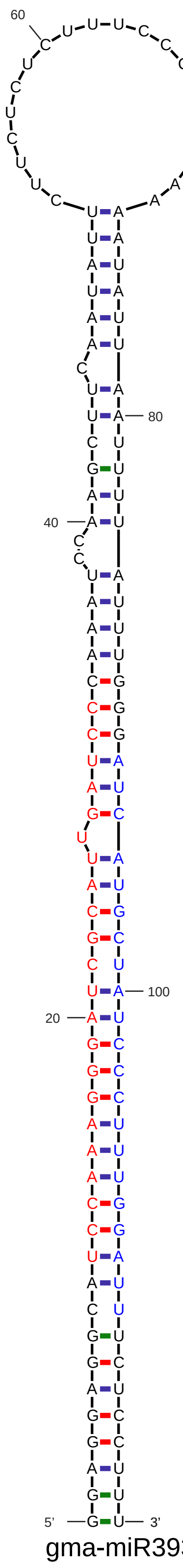

gma-miR393a

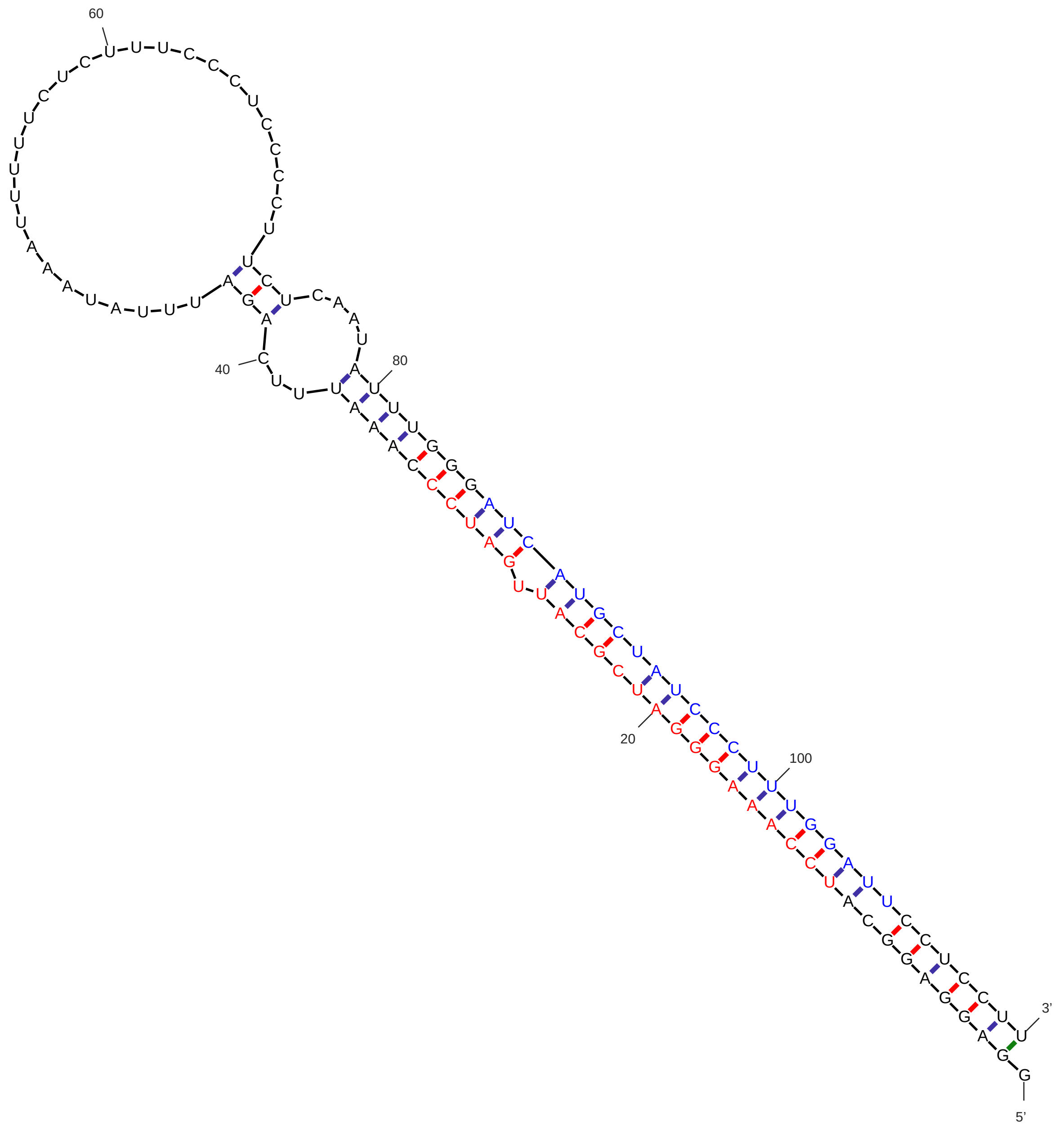

gma-miR393c

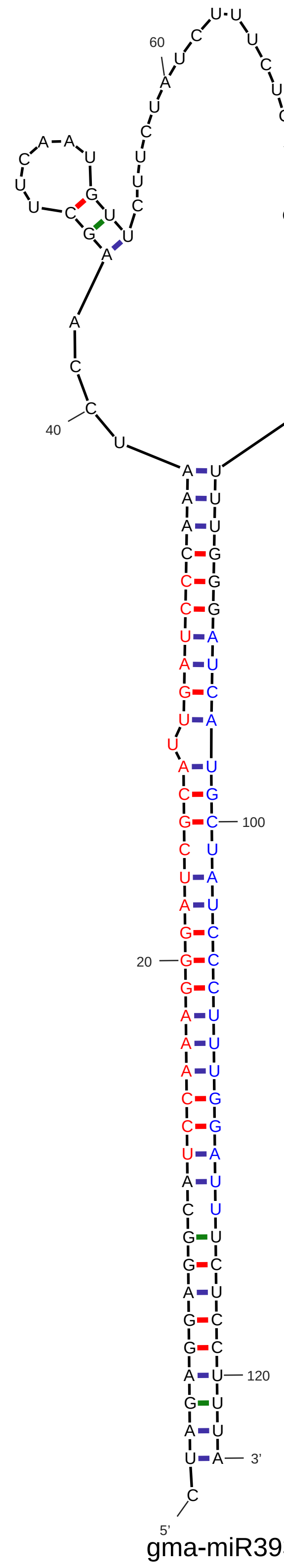

gma-miR393d

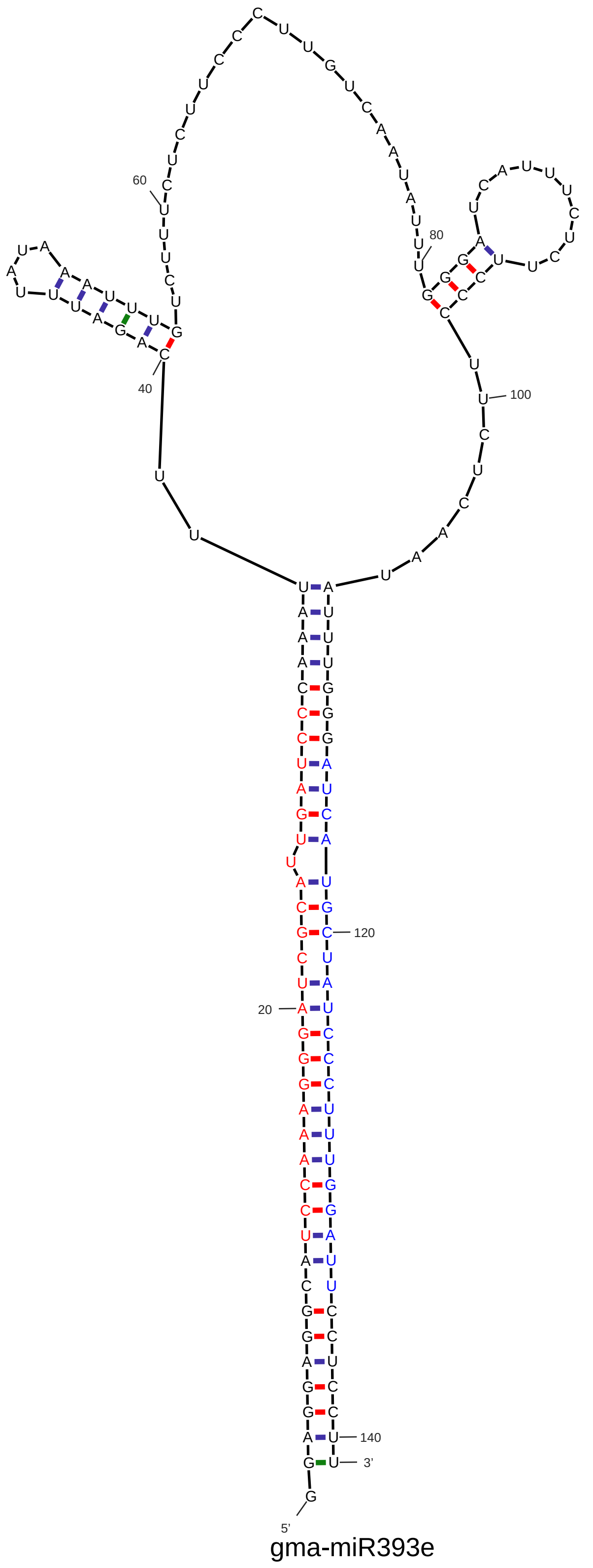

gma-miR393e

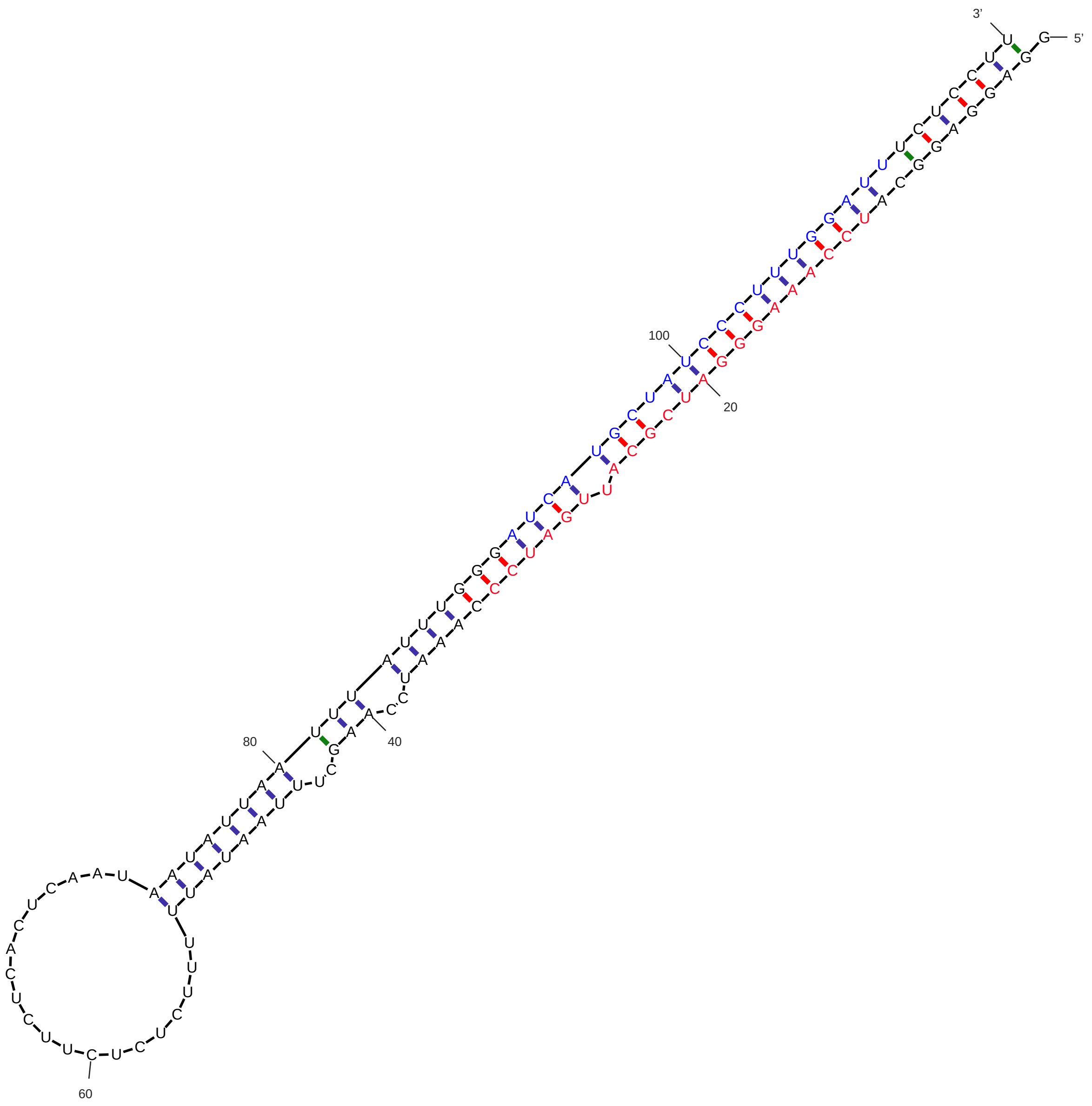

gma-miR393f

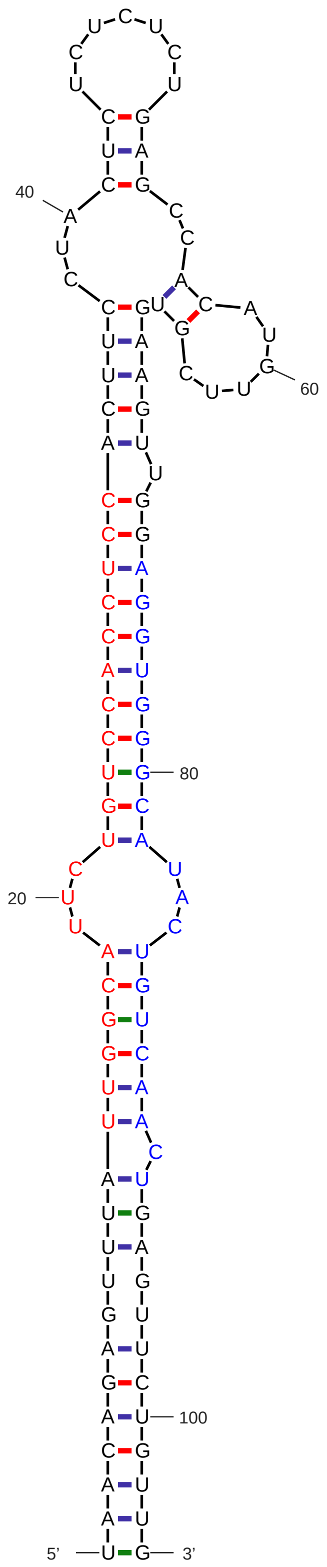

gma-miR394b

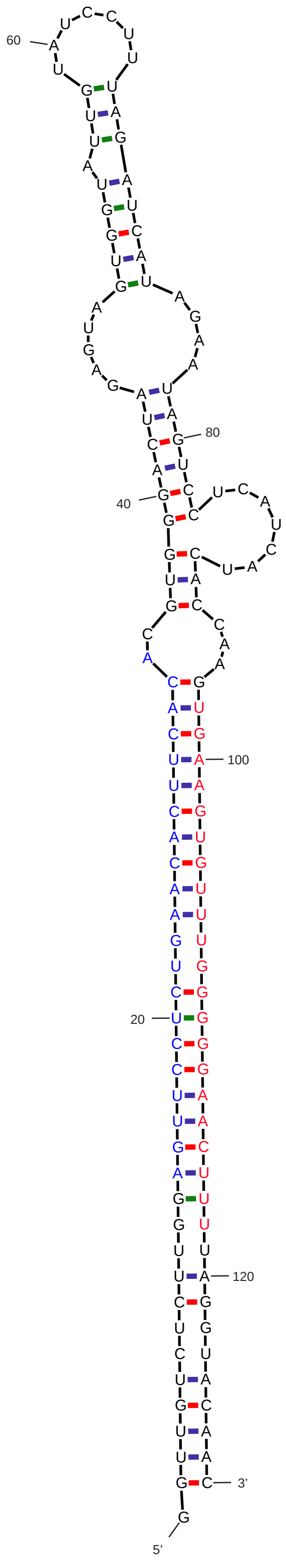
gma-miR395g

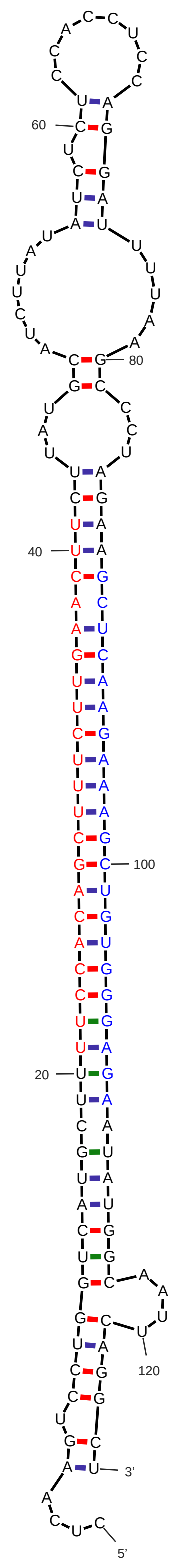
gma-miR396b

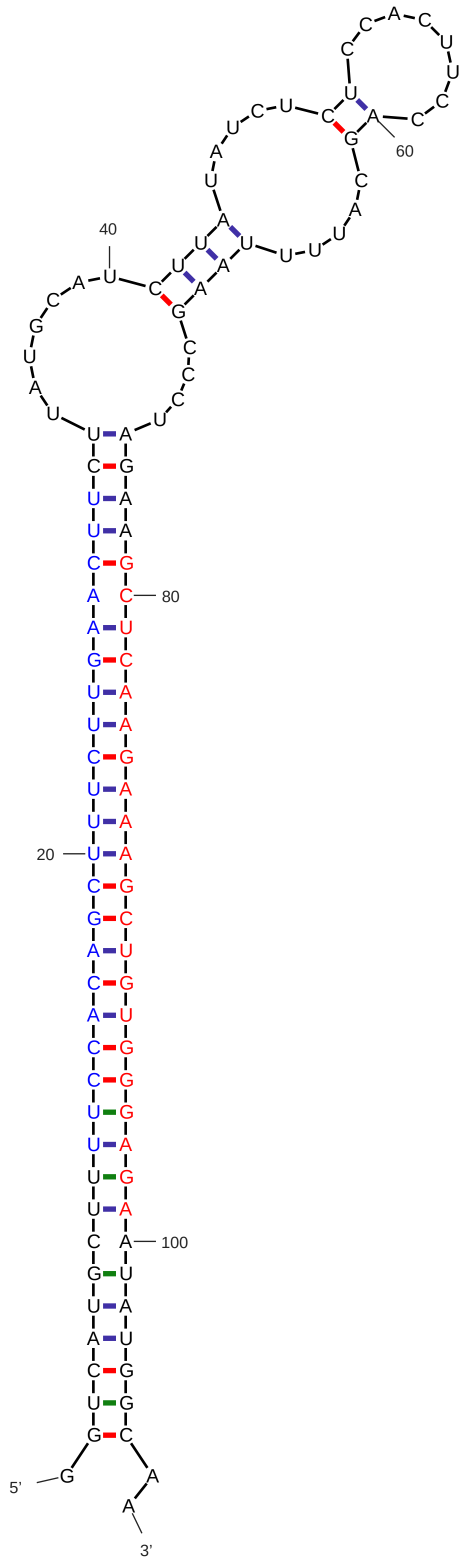
gma-miR396d

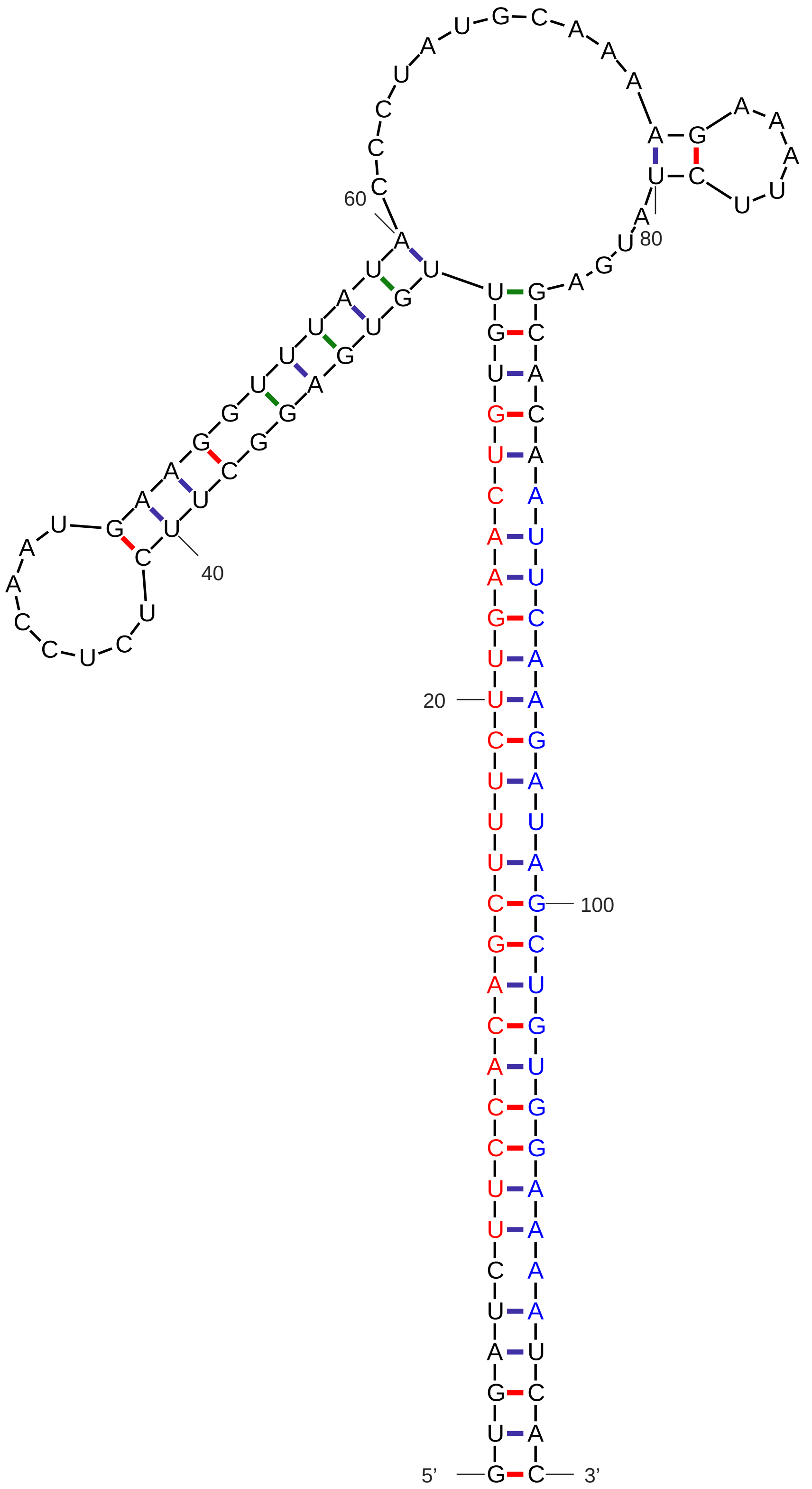
gma-miR396e

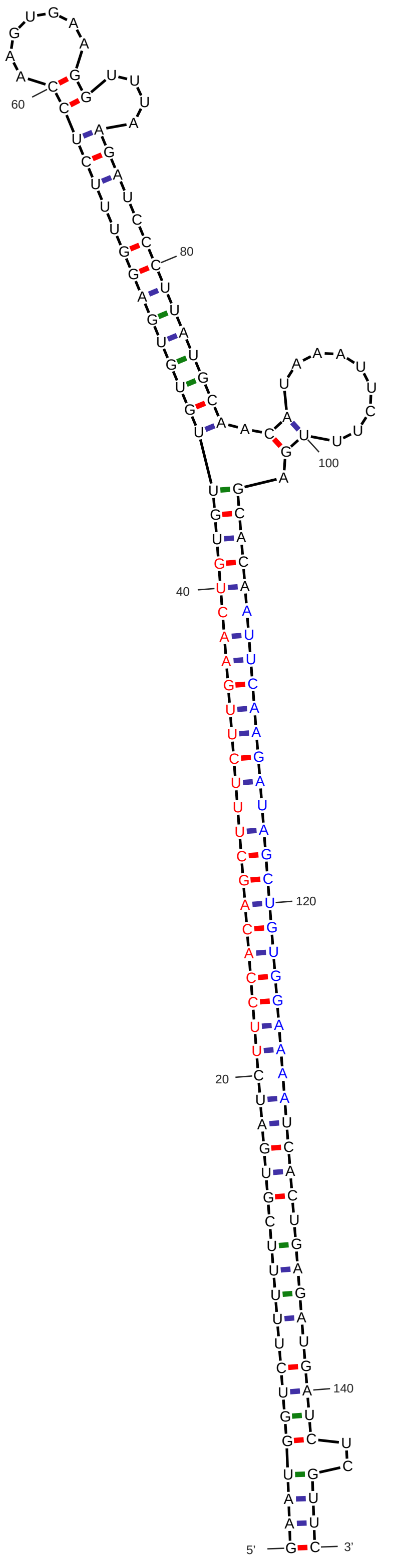

gma-miR396h

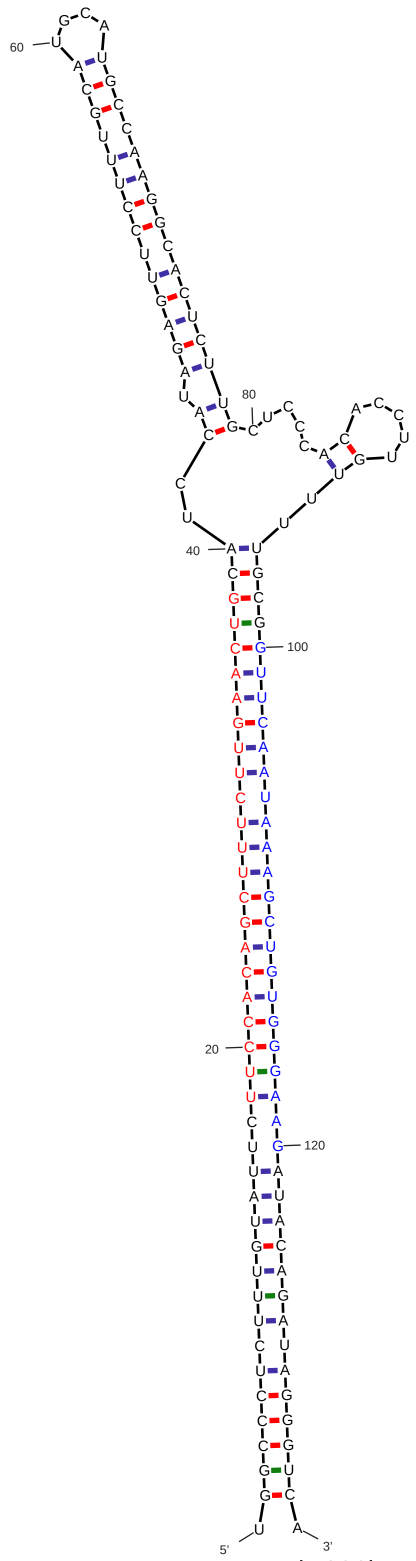

gma-miR396i

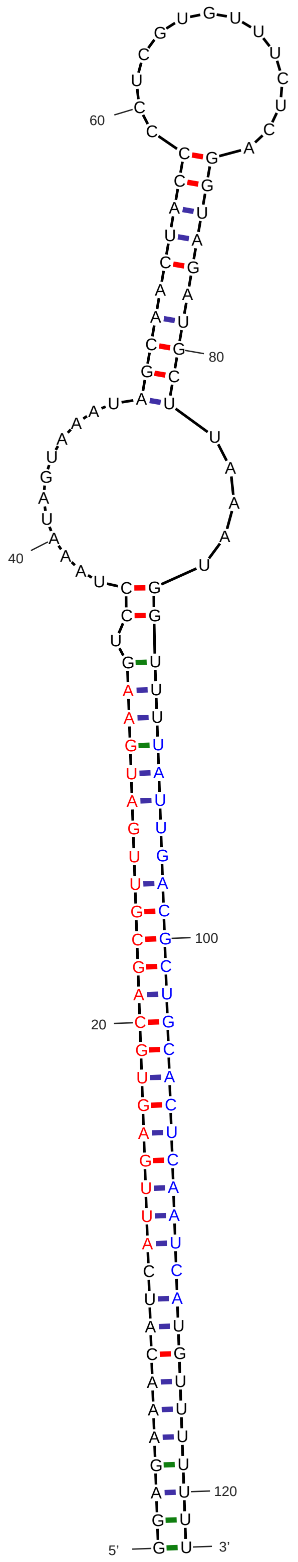

gma-miR397b

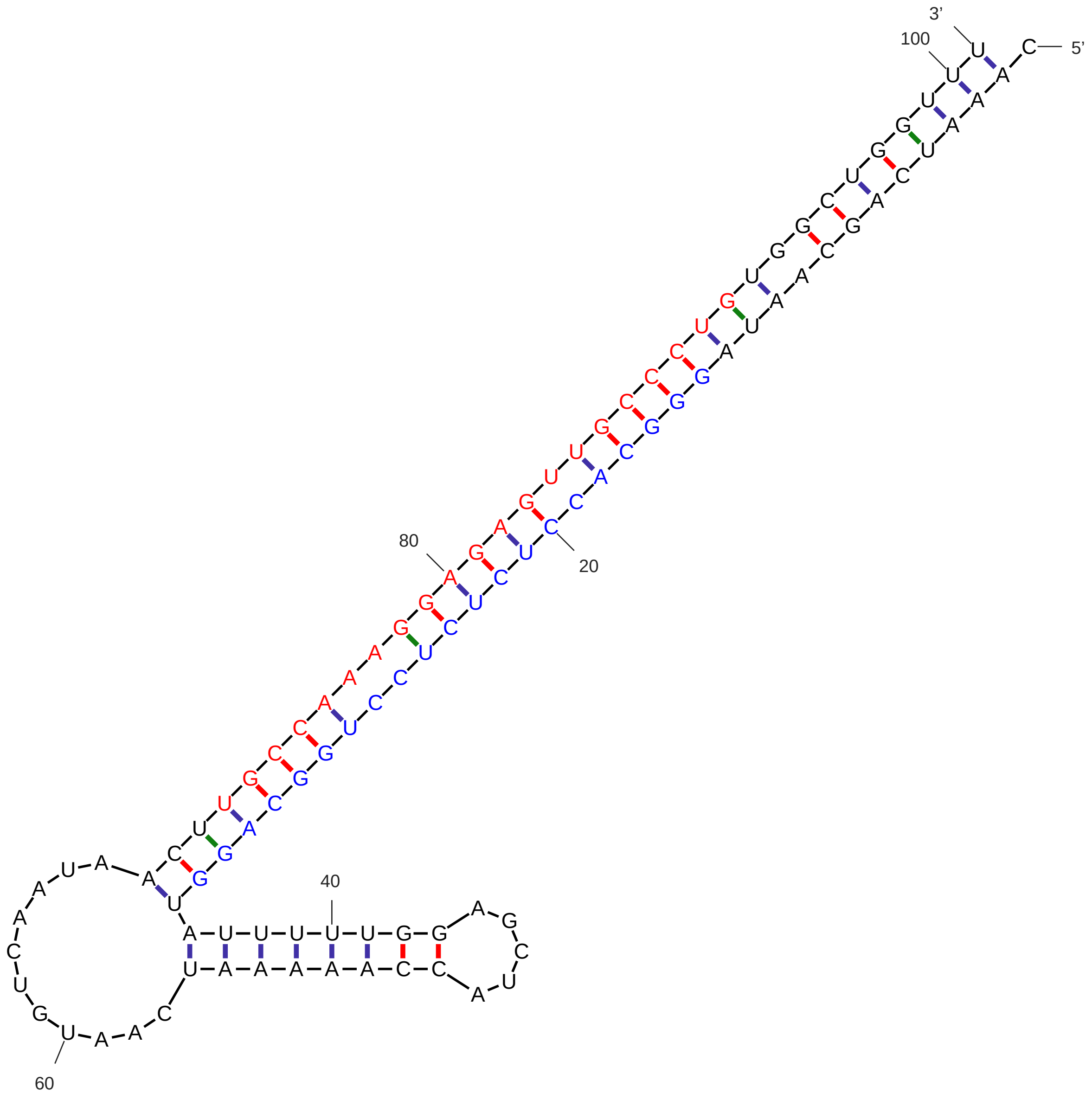

gma-miR399b

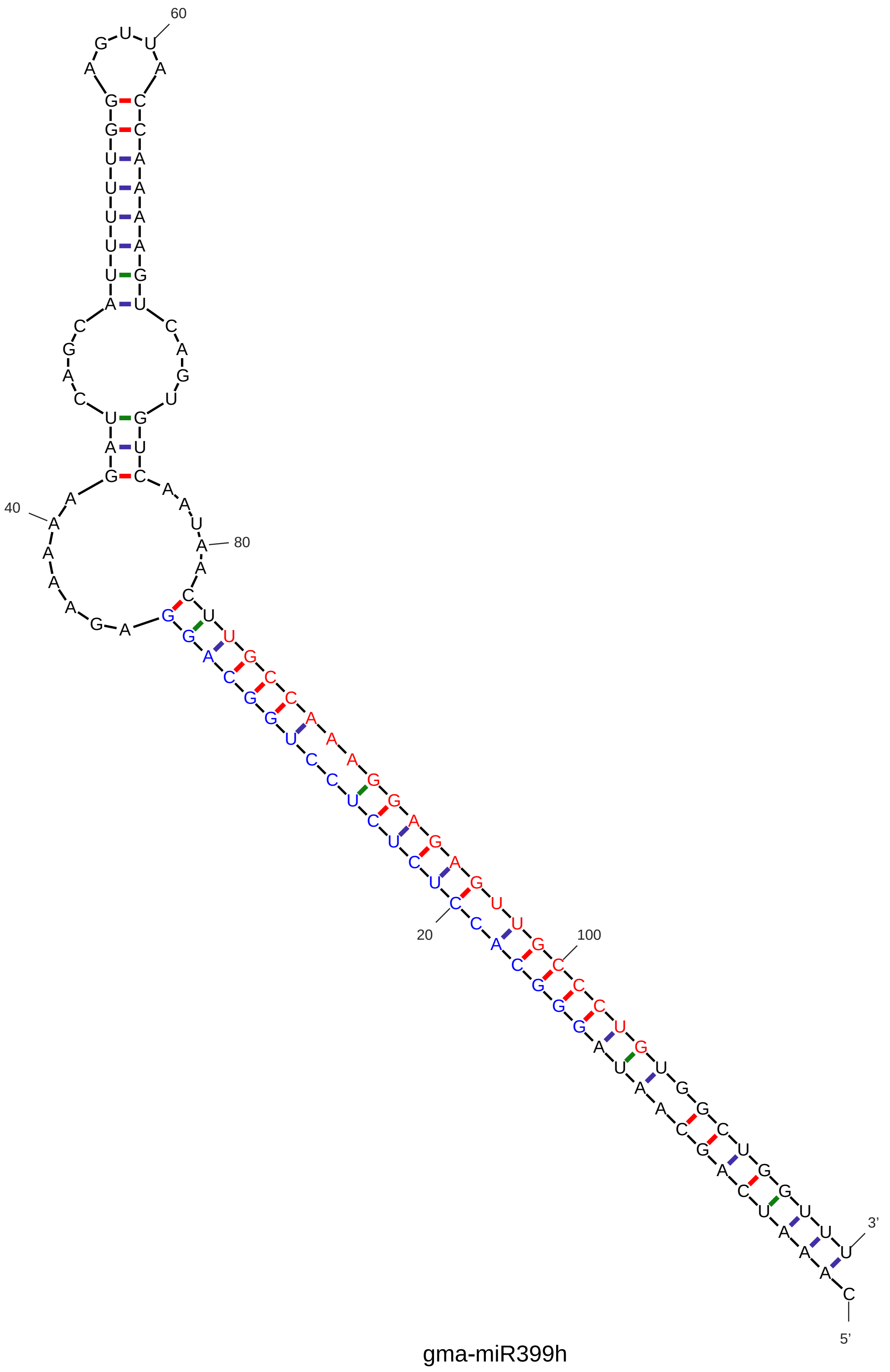

gma-miR399h

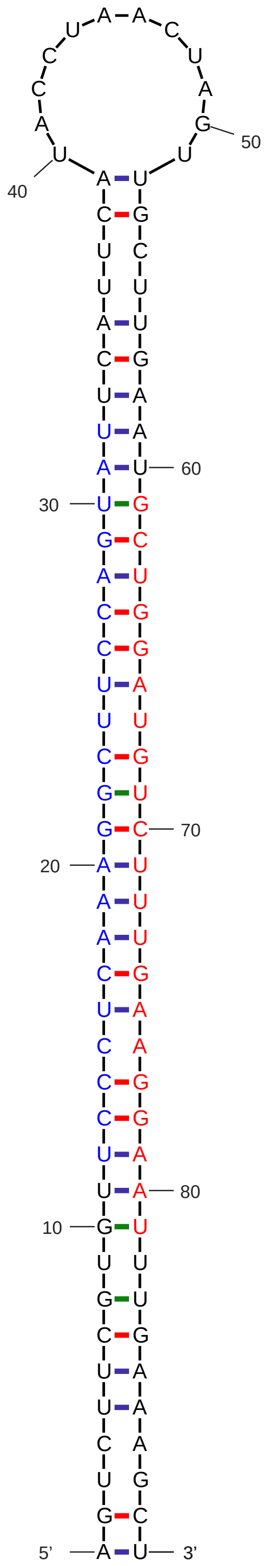

gma-miR862a

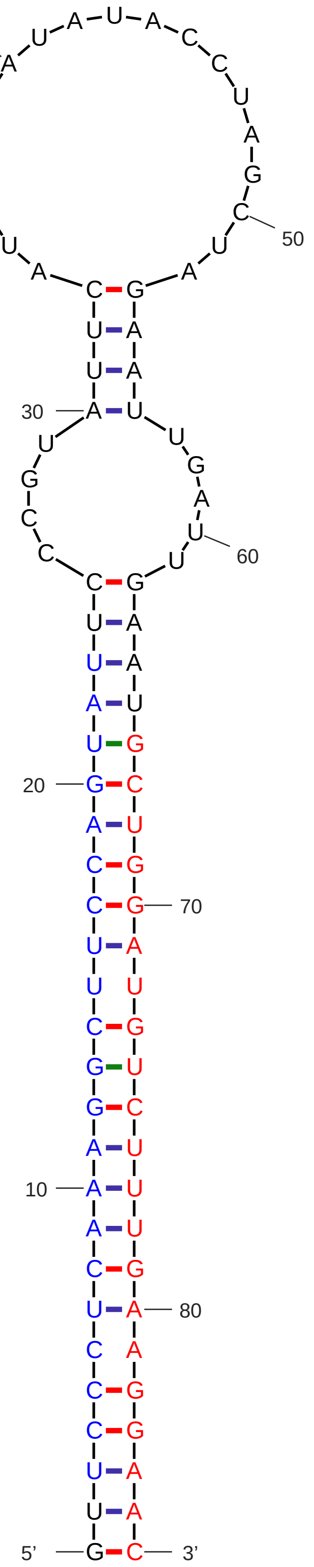

gma-miR862b

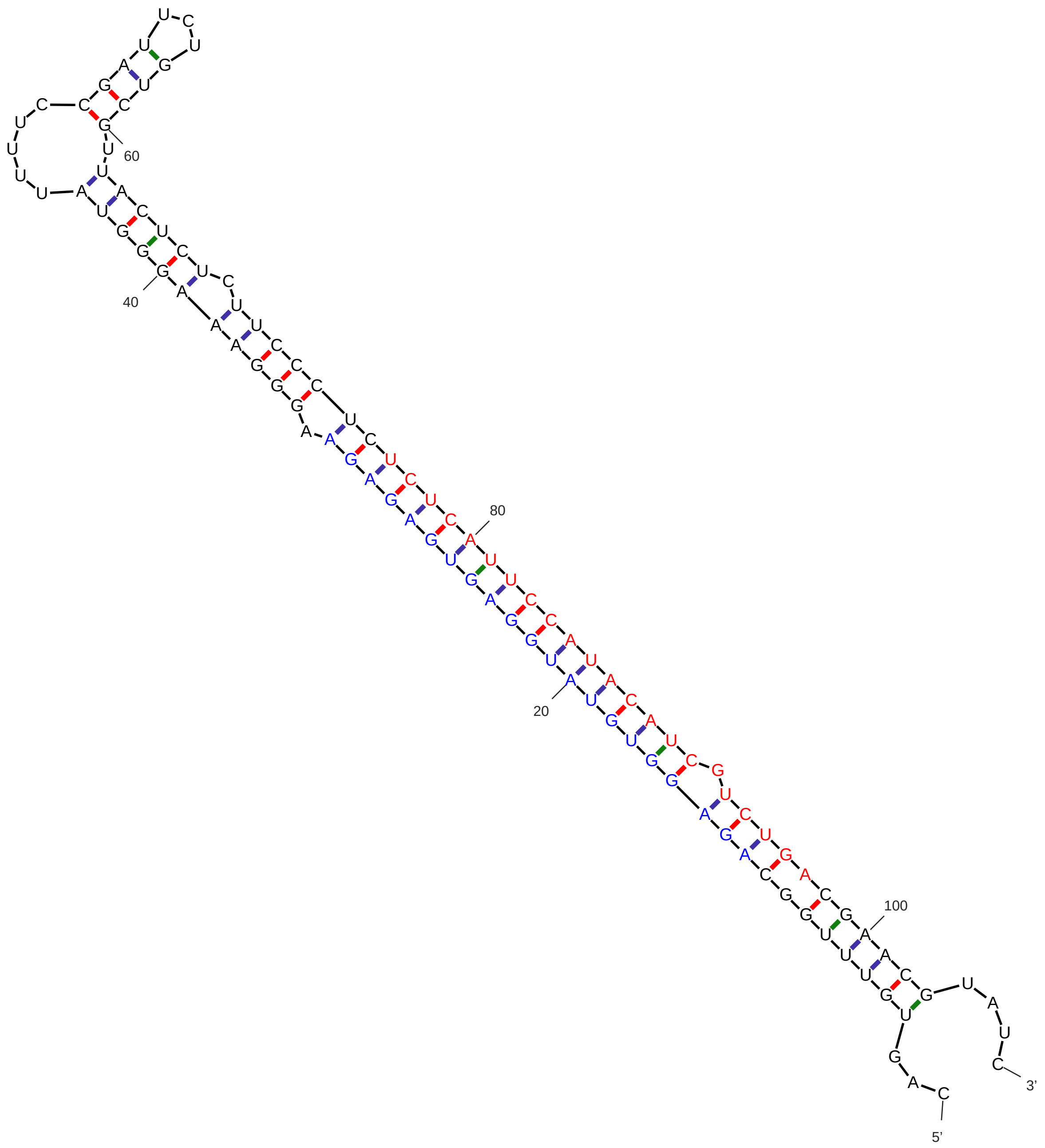

gma-miR1507a

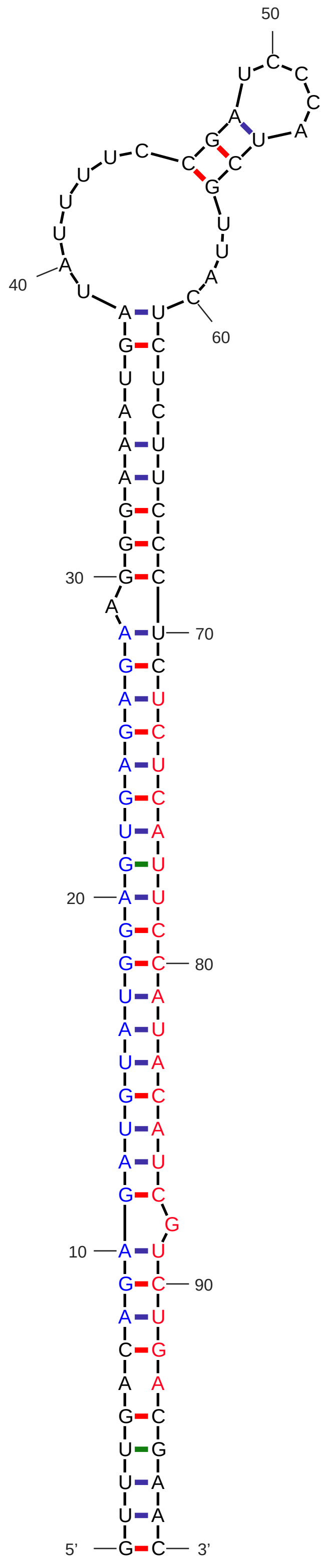

gma-miR1507b

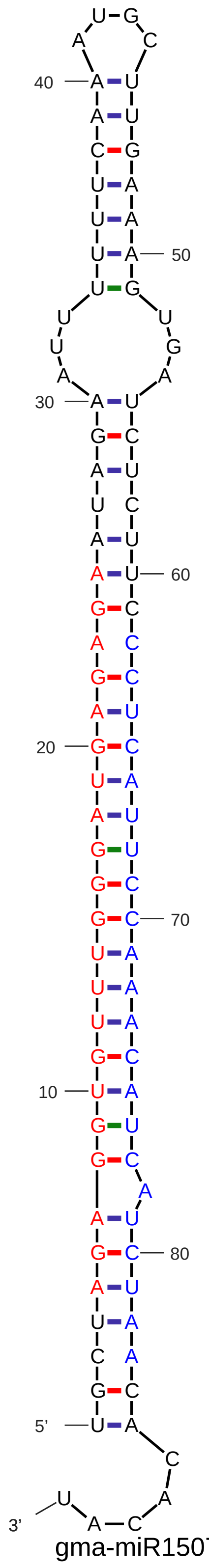

gma-miR1507c

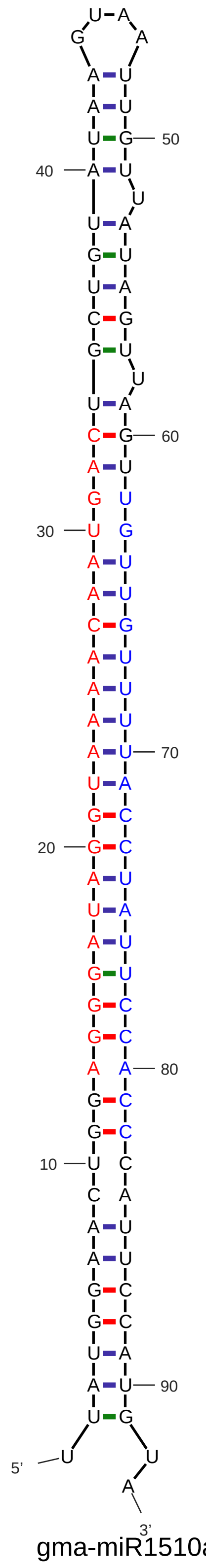

gma-miR1510a

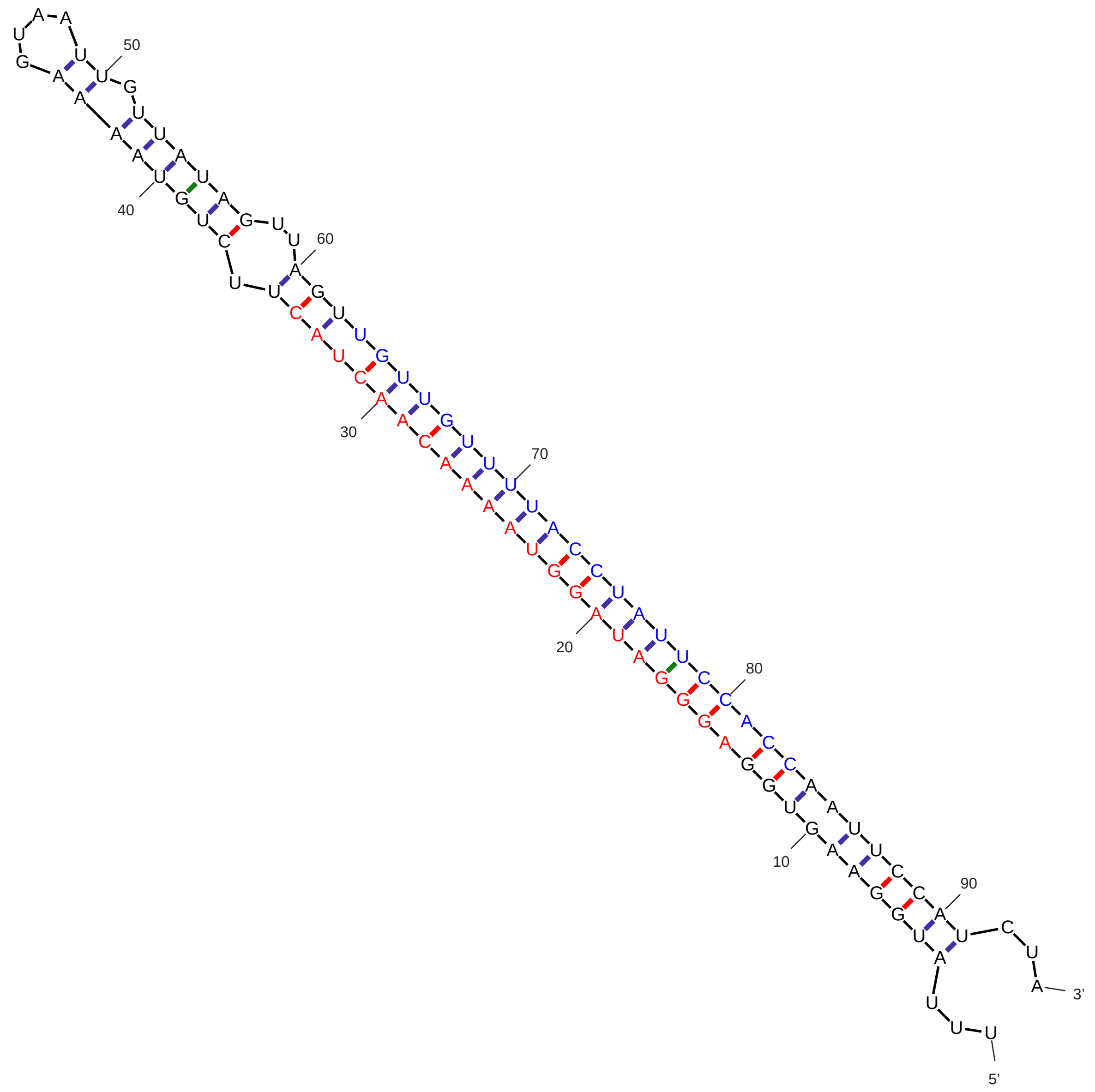

gma-miR1510b

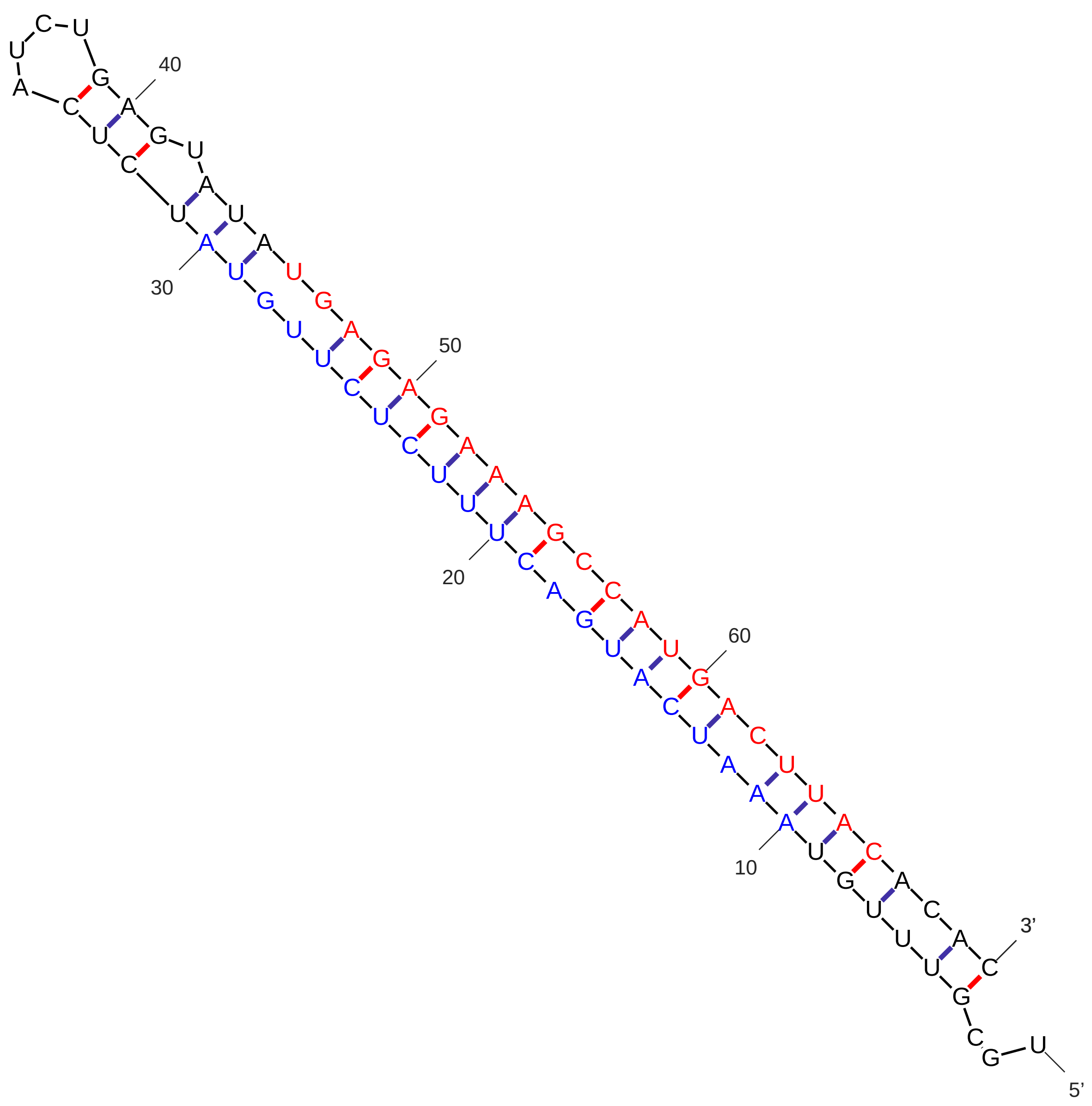

gma-miR1513b

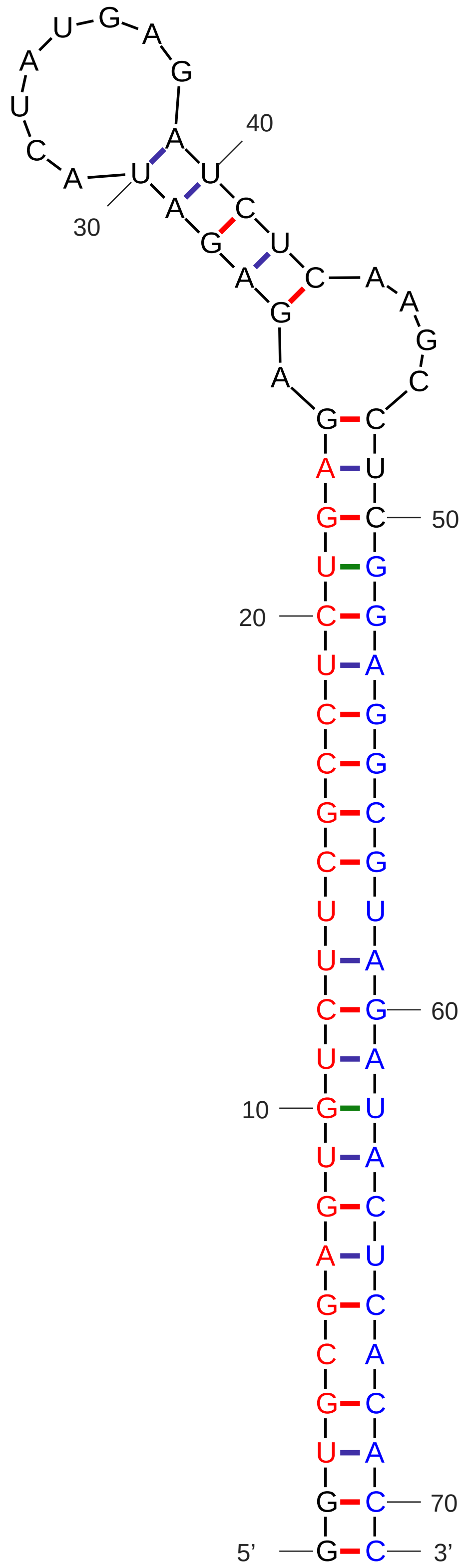

gma-miR2109

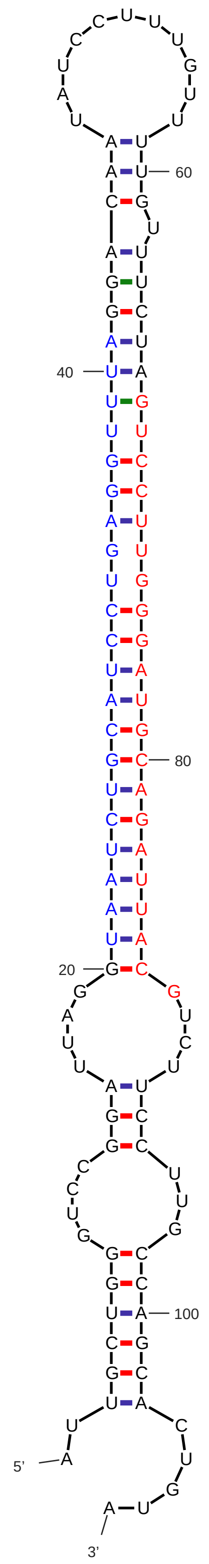

gma-miR2111a

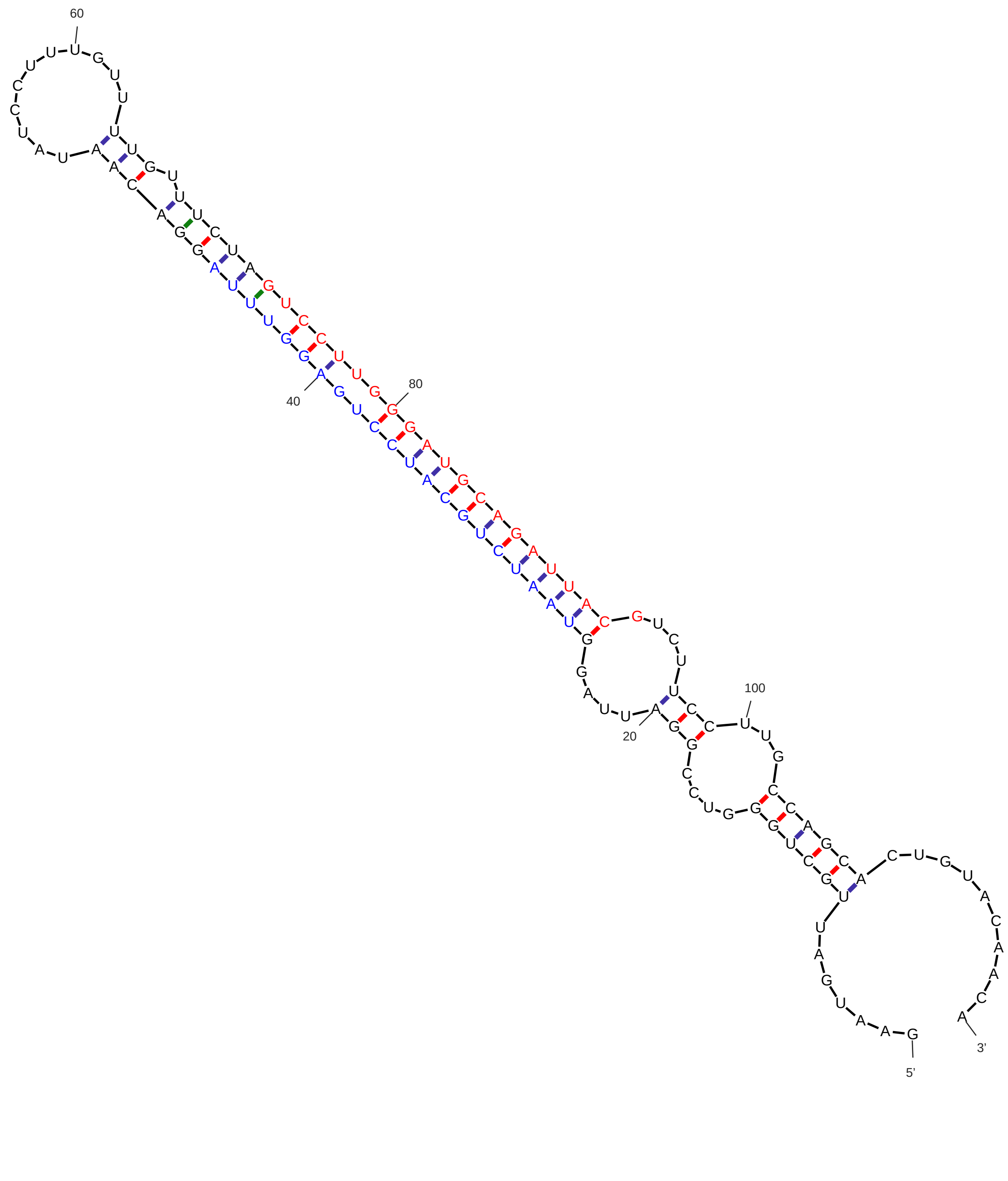

gma-miR2111d

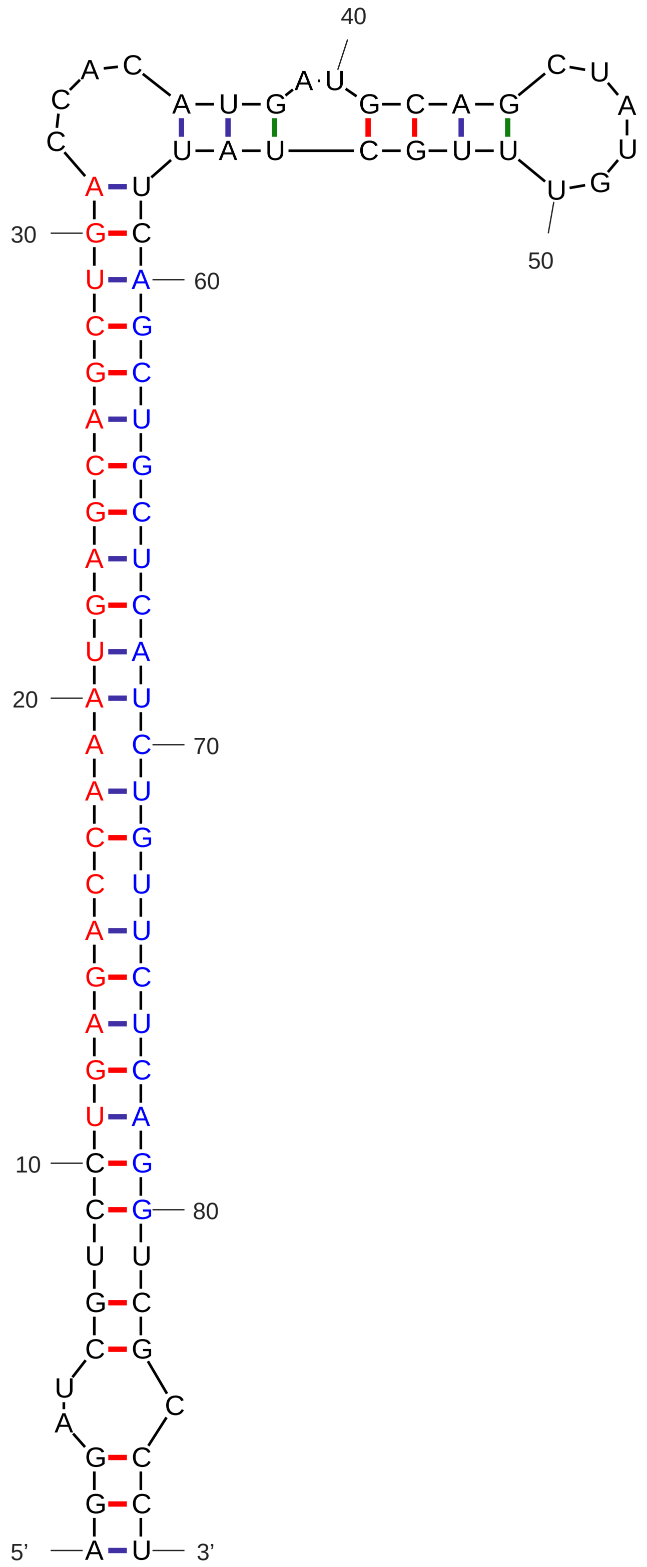
gma-miR3522

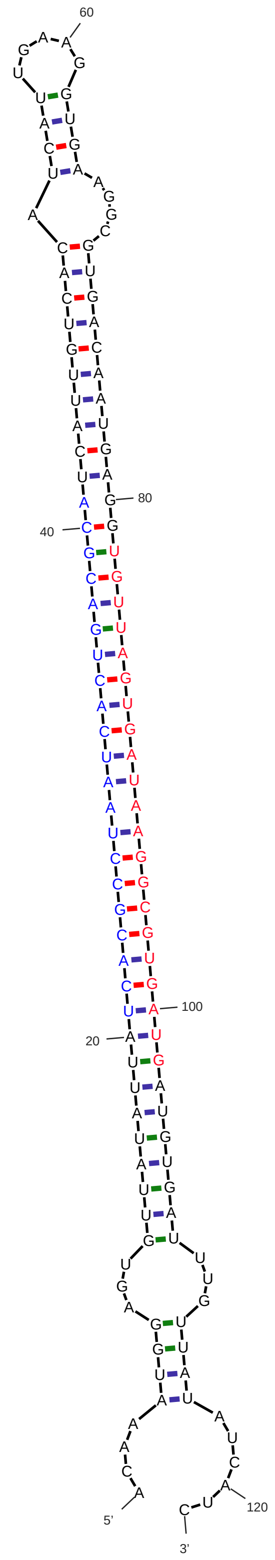
gma-miR4387e

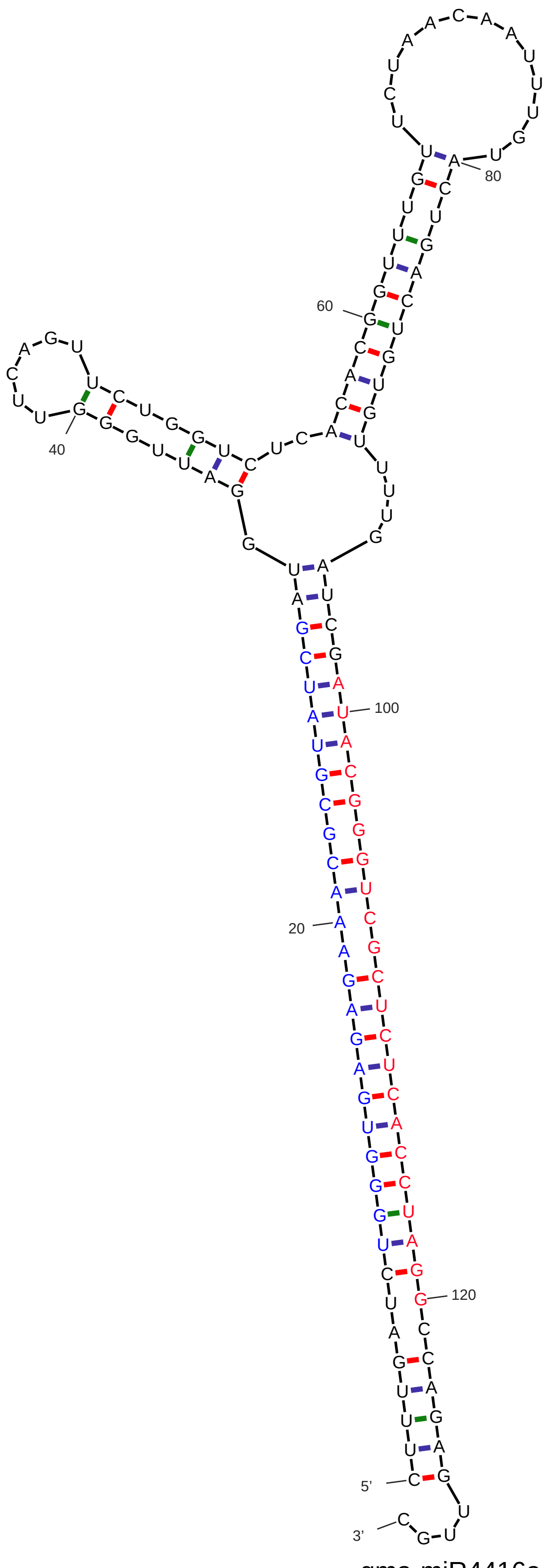
gma-miR4416a

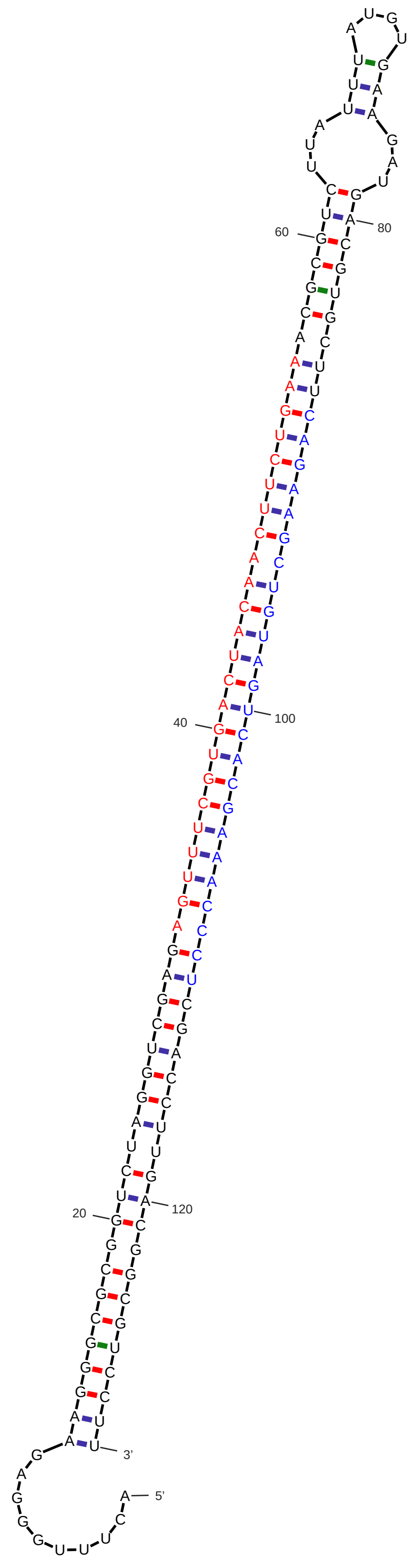
gma-miR4998

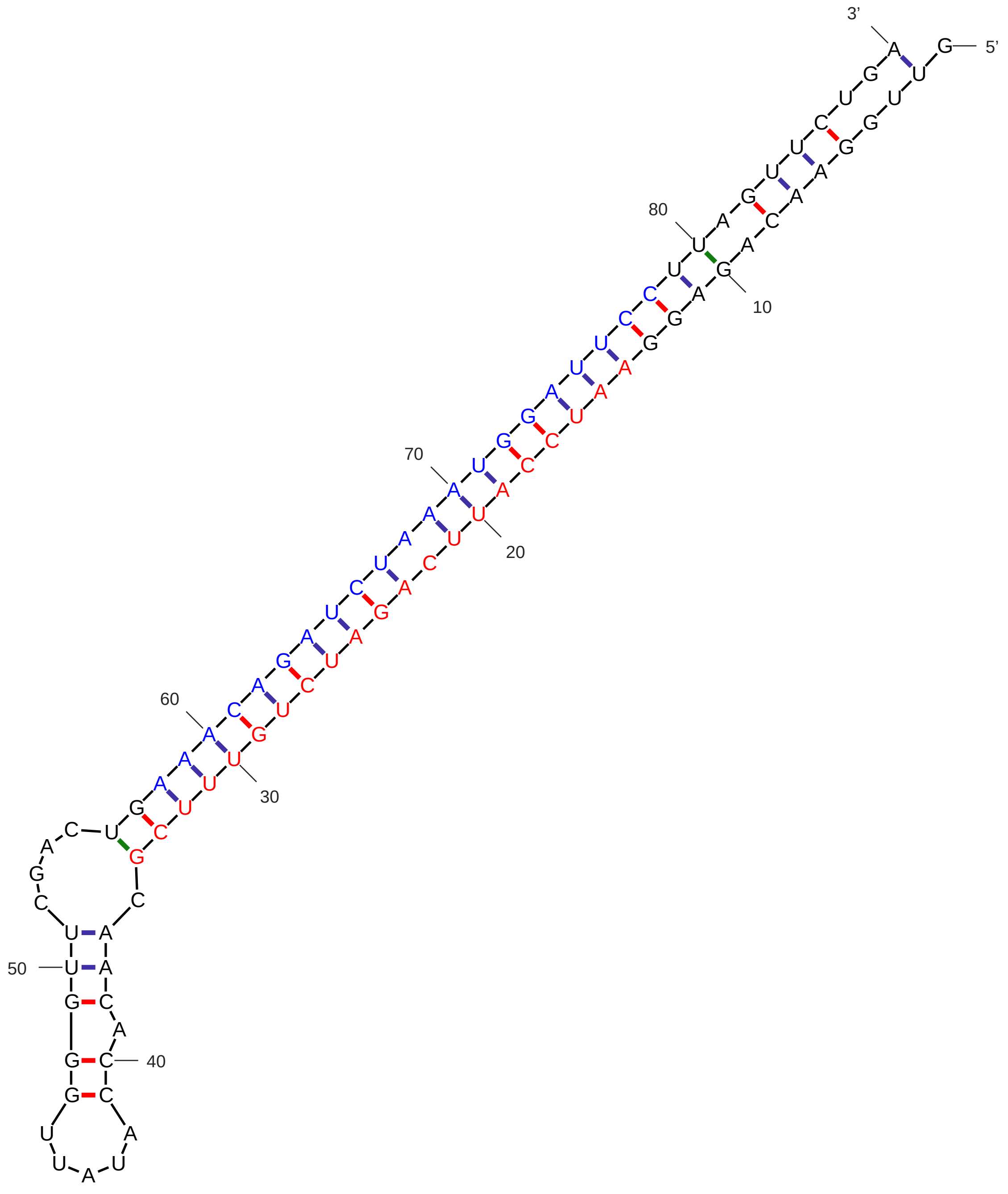

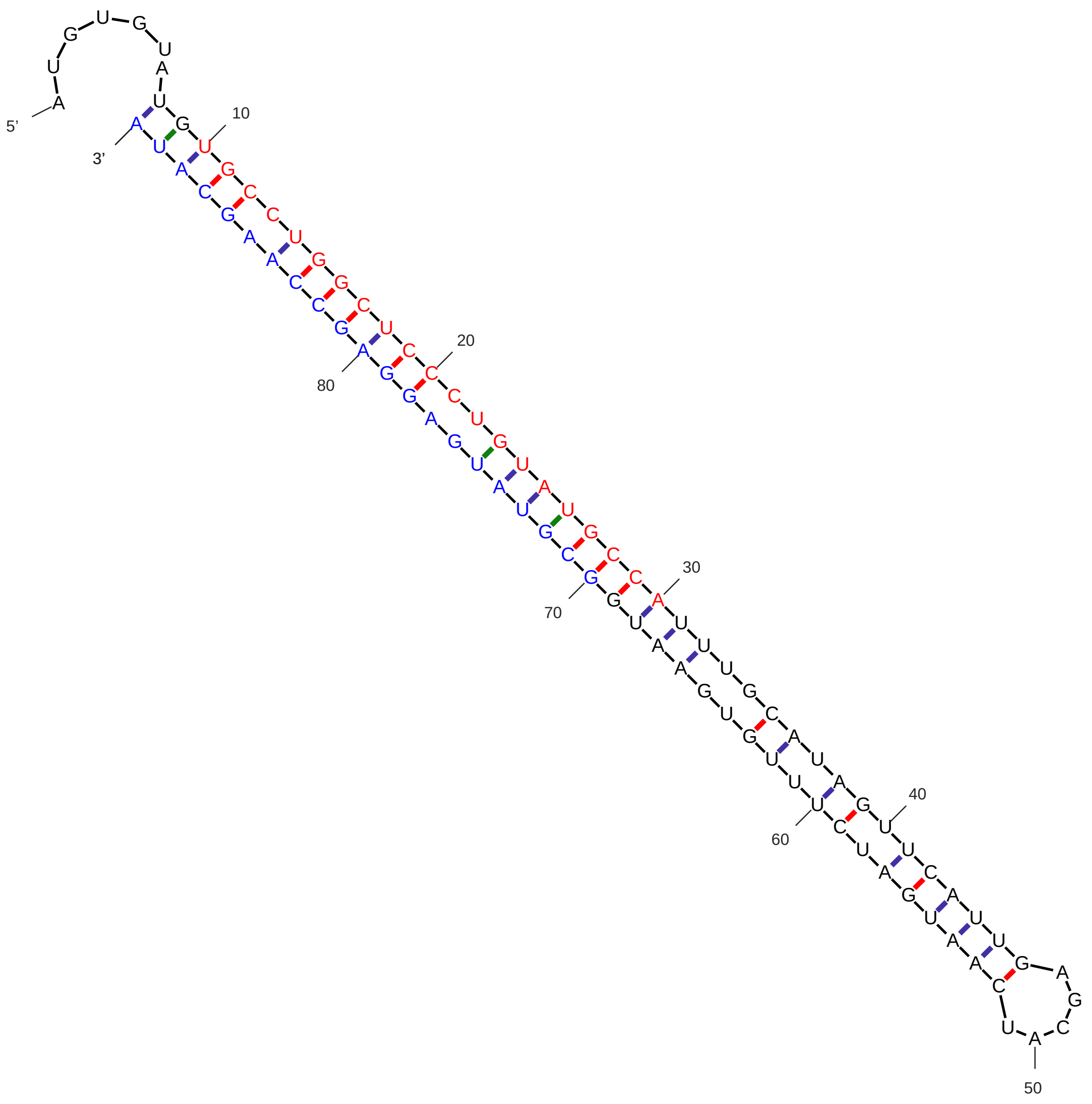

gma-miR5667

gma-miR160b

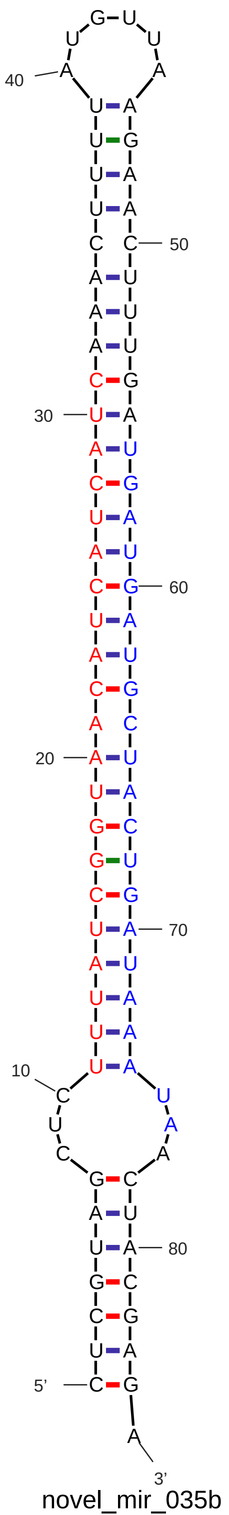

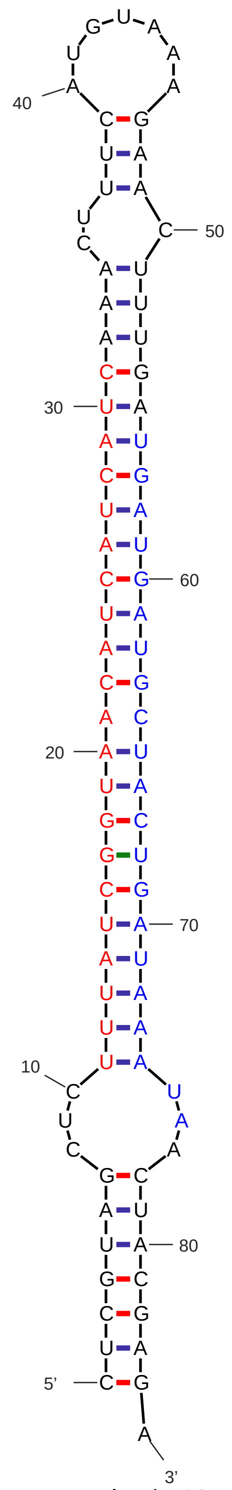

novel_mir_035b

novel_mir_035d

Figure S6. Predicted secondary structures of high-confidence miRNAs. Red font represent the miRNA-5p and blue font represent the miRNA-3p.
